# Circular RNA PTPN4 Contributes to Blood‐Brain Barrier Disruption during Early Epileptogenesis

**DOI:** 10.1002/advs.202502250

**Published:** 2025-12-14

**Authors:** Jiurong Yang, Yang Hu, Feiyu Wang, Xintao Peng, Honggang Qi, Yuanyuan Yao, Canyu Zhang, Lijie Zhou, Xuemei Liang, Kang Xu, Cong Zhang, Aifeng Zhang, Chen Chen, Yu Zeng, Chenchen Zhang, Guangming Gan, Xinjian Zhu

**Affiliations:** ^1^ Department of Pharmacology Medical School of Southeast University Nanjing 210009 China; ^2^ Sanbo Brain Hospital Capital Medical University Beijing 100093 China; ^3^ Department of Pathology Medical School of Southeast University Nanjing 210009 China; ^4^ Functional Experimental Center Medical School of Southeast University Nanjing 210009 China; ^5^ National Residents Clinical Skills Training Center Medical School of Southeast University Nanjing 210009 China; ^6^ Transmission Electron Microscopy Center Medical School of Southeast University Nanjing 210009 China; ^7^ Department of Genetics and Developmental Biology Medical School of Southeast University Nanjing 210009 China

**Keywords:** blood‐brain barrier, circPTPN4, endothelin converting enzyme‐1, epilepsy, miR‐145

## Abstract

Circular RNAs (circRNAs) are crucial regulators of gene expression, exhibiting dynamic expression patterns during both normal brain development and diverse pathological states. This study reports the elevated expression of CircPTPN4 in the brain tissue and plasma during early epileptogenesis in a murine temporal lobe epilepsy model. Overexpression of CircPTPN4 disrupts the tight junctions of brain microvascular endothelial cells (BMECs), compromising blood‐brain barrier (BBB) integrity. Mechanistically, CircPTPN4 functions as a competitive endogenous RNA by sequestering miR‐145a‐5p, thereby upregulating endothelin‐converting enzyme‐1 (ECE‐1). Subsequent ECE‐1‐mediated endothelin‐1 (ET‐1) production activates the p38/MAPK pathway, downregulating tight junction protein expression. Knockdown of CircPTPN4 attenuated tight junction disruption and BBB impairment during acute epileptogenesis while reducing spontaneous seizure frequency in chronic epilepsy. These findings establish the CircPTPN4/miR‐145a‐5p/ECE‐1 axis as both a diagnostic biomarker and therapeutic target for BBB preservation in early‐stage epilepsy.

## Graphical abstract

Epileptic condition induces CircPTPN4 upregulation, which promotes ECE‐1 expression through competitive sequestration of miR‐145a‐5p. The elevated ECE‐1 catalyzes the ET‐1 production, leading to p38/MAPK pathway activation and subsequent downregulation of tight junction protein expression. This cascade results in increased BBB permeability and enhanced infiltration of inflammatory cytokines into brain parenchyma, both of which collectively contribute to heightened seizure susceptibility.

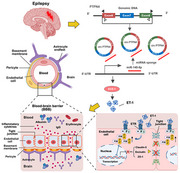

## 1 Introduction

Epilepsy is a chronic neurological disorder characterized by the sudden, abnormal neuronal hyperexcitability that disrupts normal brain function. The condition manifests through diverse clinical presentations, including motor disturbances, sensory abnormalities, altered consciousness, and psychiatric comorbidities, collectively imposing substantial burdens on patient health and quality of life. A growing body of evidence indicates that epileptic seizure induces neuropathological changes, which involve complex structural and functional alterations of neurons, glial cells, and cerebral microvessels.^[^ ^1^, ^2^, ^3^, ^4^ ^]^ The BBB‐ a specialized neurovascular unit comprising microvascular endothelial cells, basement membrane, astrocyte end‐feet, and pericytes‐ plays a crucial role in maintaining cerebral homeostasis.^[^ ^5^, ^6^, ^7^ ^]^ Endothelial tight junctions (TJs) are critical for BBB integrity,^[^ ^6^, ^7^ ^]^ mediating essential neurovascular interactions that preserve the brain's microenvironmental stability.^[^ ^8^, ^9^, ^10^ ^]^ Both animal and clinical studies have demonstrated epilepsy‐associated ultrastructural BBB abnormalities, including TJ disruption and increased vascular permeability.^[^ ^11^, ^12^, ^13^, ^14^ ^]^ Emerging evidence indicates that BBB dysfunction may not merely represent a consequence of epileptic seizures, but rather actively contributes to both epileptogenesis and disease progression.^[^ ^15^, ^16^ ^]^ However, the precise molecular mechanisms underlying seizure‐induced BBB impairment remain incompletely understood. Elucidating these pathophysiological processes represents a crucial research priority with important therapeutic implications for epilepsy management.

CircRNAs are a recently discovered class of endogenous non‐coding RNAs that form covalently closed loops through back‐splicing mechanisms. This unique circular conformation confers exceptional stability and resistance to exonuclease degradation compared to linear RNAs.^[^ ^17^, ^18^, ^19^ ^]^ Advances in bioinformatics and high‐throughput sequencing technologies^[^ ^20^ ^]^ have enabled the identification of numerous endogenous circRNAs in eukaryotic cells, many of which exhibit tissue‐specific and spatiotemporally regulated expression patterns indicative of their gene regulatory functions.^[^ ^21^, ^22^ ^]^ Notably, circRNAs are particularly abundant in mammalian brain tissues, displaying remarkable diversity across different neural regions.^[^ ^23^, ^24^, ^25^ ^]^ Their expression undergoes dynamic modulation during both brain development and pathological states,^[^ ^26^, ^27^ ^]^ suggesting critical roles in the central nervous system (CNS) physiology and disease. Emerging evidence implicates circRNAs in maintaining normal brain function and potentially regulating the pathogenesis of neurological disorders. The involvement of circRNAs in CNS pathologies has become a major research focus, with demonstrated roles in stroke, Alzheimer's disease, and Parkinson's disease.^[^ ^28^, ^29^ ^]^ While recent studies have identified epilepsy‐associated circRNA expression alterations,^[^ ^30^, ^31^, ^32^ ^]^ their precise contributions to epileptogenesis and seizure‐related brain injury remain poorly understood. Particularly, their involvement in post‐epileptic BBB dysfunction has not been explored. Elucidating the mechanisms by which circRNAs mediate BBB injury following seizures could yield crucial insights into epilepsy pathogenesis and reveal novel diagnostic biomarkers and therapeutic targets for seizure‐induced neurological damage.

## 2 Results

### 2.1 BBB Integrity was Disrupted during Early Epileptogenesis

**Figure 1:**
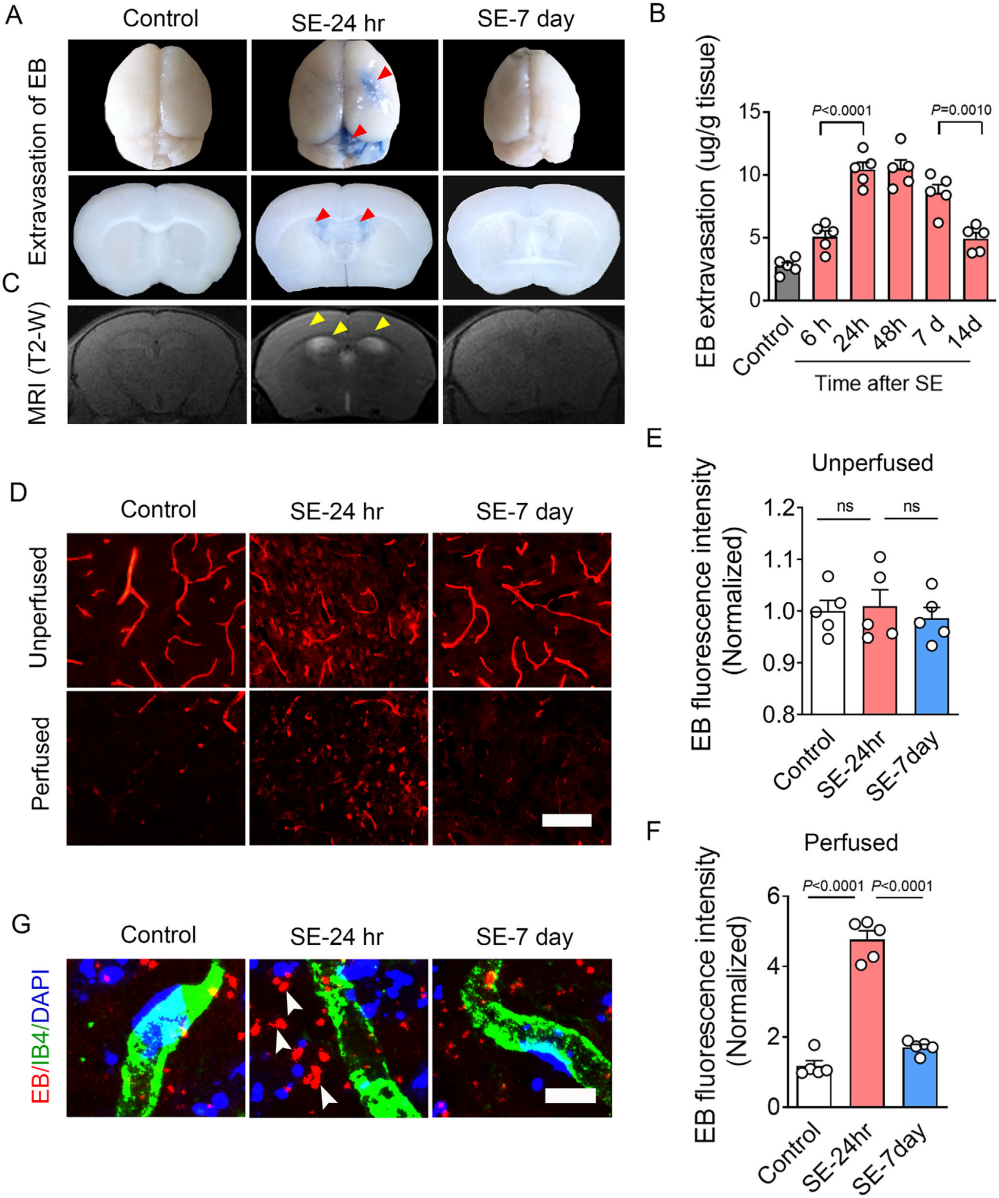
BBB integrity was compromised during early epileptogenesis. A) Representative images of the whole brain and coronal sections showing evans blue (EB) extravasation into the brain parenchyma in Control, 24‐h, and 7‐day post‐status epilepticus (SE) mice (Red arrowheads indicate EB extravasation sites). B) EB extravasation was quantified in brain parenchyma at 6 h, 24 h, 48 h, 7 days, and 14 days post‐SE (*n =* 5; *p <* 0.0001, SE‐24 h versus SE‐6 h; *p =* 0.001, SE‐14 day versus SE‐7 day). C) In vivo T2‐weighted MRI images showing region‐dependent cerebral edema of 24 h post‐SE mice (Yellow arrowheads indicate regions of severe edema). D) Representative images of EB autofluorescence in the cortical region of Control, 24‐h, and 7‐day post‐SE mice, before and after perfusion. E) Bar graph showing the quantification of EB fluorescence intensity at 24 h and 7 days post‐SE of unperfused mice (*n =* 5; ns, SE‐24 h versus Control; ns, SE‐7 day versus SE‐24 h). F) Bar graph showing the quantification of EB fluorescence intensity at 24 h and 7 days post‐SE of perfused mice (*n =* 5; *p <* 0.0001, SE‐24 h versus Control; *p <* 0.0001, SE‐7 day versus SE‐24 h). G) Representative images of EB/IB4 co‐immunostaining in the cortex of Control, 24‐h, and 7‐day post‐SE mice after perfusion (White arrowheads indicate severe EB infiltration in the extravascular region of the cortex at 24 h post‐SE). Values are expressed as the mean ± S.E.M. Statistical analyses were performed using one‐way ANOVA followed by Tukey's post hoc test. Scale bar = 25 µm in (D) and 10 µm in (G).

**Figure 2:**
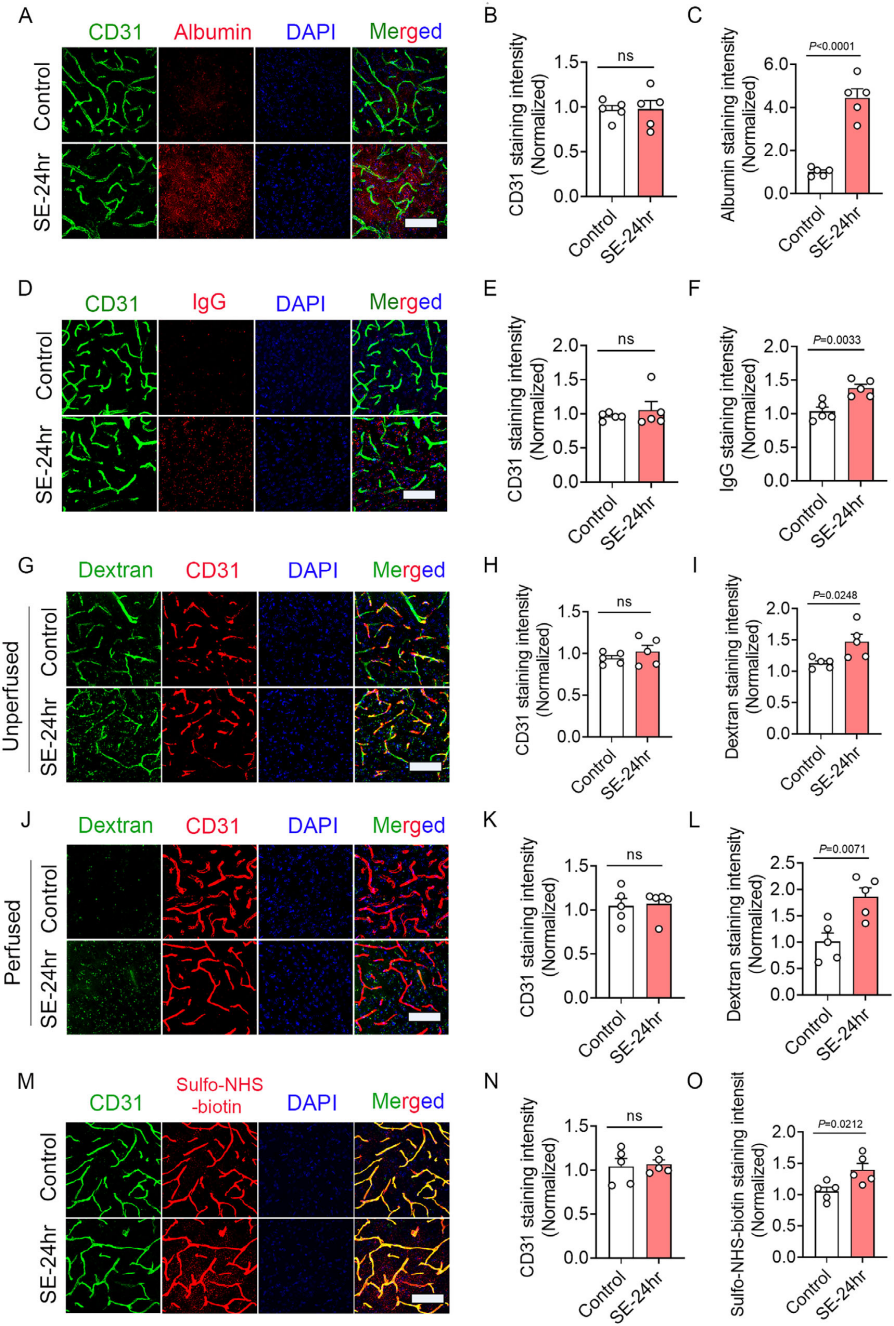
Increased BBB permeability to endogenous proteins and exogenous tracers in the cortical region of SE‐24 h mice. A) Representative images of CD31/Albumin co‐immunostaining in the cortex of Control and SE‐24 h mice. B, C) Bar graph quantifying the mean fluorescence intensity of CD31 (*n =* 5; ns; SE‐24 h versus Control) and albumin (*n =* 5, *p <* 0.0001, SE‐24 h versus Control). D) Representative images of CD31/IgG co‐immunostaining in the cortex of Control and SE‐24 h mice. E, F) Bar graph quantifying the mean fluorescence intensity of CD31 (*n =* 5, ns, SE‐24 h versus Control) and IgG (*n =* 5, *p =* 0.0033, SE‐24 h versus Control). G) Representative images of CD31/Dextran co‐immunostaining in the cortex of Control and SE‐24 h unperfused mice. H, I) Bar graph quantifying the mean fluorescence intensity of CD31 (*n =* 5, ns, SE‐24 h versus Control) and Dextran (*n =* 5, *p =* 0.0248, SE‐24 h versus Control). J) Representative images of CD31/Dextran co‐immunostaining in the cortex of Control and SE‐24 h perfused mice. K, L) Bar graph quantifying the mean fluorescence intensity of CD31 (*n =* 5; ns; SE‐24 h versus Control) and Dextran (*n =* 5, *p =* 0.0071, SE‐24 h versus Control). M) Representative images of CD31/Sulfo‐NHS‐Biotin co‐immunostaining in the cortex of Control and SE‐24 h perfused mice. N, O) Bar graph quantifying the mean fluorescence intensity of CD31 (*n =* 5, ns, SE‐24 h versus Control) and Sulfo‐NHS‐Biotin (*n =* 5, *p =* 0.0212, SE‐24 h versus Control). Data are presented as means ± S.E.M. Statistical analyses were performed using unpaired two‐tailed Student's *t*‐test. Scale bar = 50 µm.

Evans Blue (EB) is an azo dye that binds strongly to plasma albumin and can cross the BBB when there is vascular injury, as seen in various brain disorders. To assess EB leakage in the brain parenchyma of epileptic mice, we first performed cardiac perfusion to remove intravascular EB and to examine EB extravasation into the parenchyma. Our results revealed prominent EB staining in the cortical parenchyma 24 h after Status Epilepticus (SE‐24 h) in the pilocarpine‐induced temporal lobe epilepsy model, indicating BBB leakage in these brain regions. By day 7 post‐SE (SE‐7 day), EB staining intensity was reduced, suggesting recovery of BBB integrity (**Figure** **1**A). Quantification of EB content at different time points showed peak EB extravasation in the cortex at 24 and 48 h after SE, followed by a progressive decline at 7 and 14 days post‐SE (Figure 1B). To further investigate BBB disruption during epileptogenesis, we performed T2‐weighted MRI scans and revealed an increase in T2‐weighted signal of edema at 24 h post‐SE, with a subsequent decrease at 7 days post‐SE, correlating with BBB recovery (Figure 1C). To further assess BBB integrity, EB autofluorescence was examined in both the vasculature and the parenchyma. In Control mice prior to perfusion, EB fluorescence was primarily localized to the vasculature. In contrast, epileptic mice at 24 h post‐SE exhibited EB extravasation into the cortical parenchyma. By 7 days post‐SE, parenchymal EB fluorescence had diminished, although total EB fluorescence intensity remained comparable across groups before perfusion (Figure 1D upper panel; Figure 1E). Following perfusion, EB was largely cleared from the vasculature, while parenchymal fluorescence intensity was remarkably increased at 24 h post‐SE but reduced by 7 days post‐SE (Figure 1D bottom panel; Figure 1F,G). To further evaluate BBB permeability, immunofluorescence staining was performed for endogenous albumin and IgG. Increased extravasation of both albumin (**Figure** **2**A–C) and IgG (Figure 2D–F) was observed in epileptic mice, indicating compromised BBB integrity. We next quantified BBB permeability by intravenously administration of exogenous FITC‐Dextran (40 kDa) conjugates. Prior to perfusion, dextran was restricted to the vasculature in Control mice, whereas obvious parenchymal leakage (evident as extravascular green fluorescence) was detected in SE‐24 h mice (Figure 2G–I). Post perfusion, SE‐24 h mice exhibited pronounced dextran accumulation in the parenchyma compared to Control mice (Figure 2J–L). To examine BBB permeability to small molecules, Sulfo‐NHS‐biotin (557 g mole^−1^) was injected, followed by staining for covalent biotin adducts. As a membrane‐impermeable tracer, Sulfo‐NHS‐biotin binds to surface‐exposed proteins and primary amines.^[^ ^33^ ^]^ In Control mice, Sulfo‐NHS‐biotin was strictly confined to the vasculature, whereas SE‐24 h mice displayed extensive parenchymal extravasation (Figure 2M–O).

**Figure 3:**
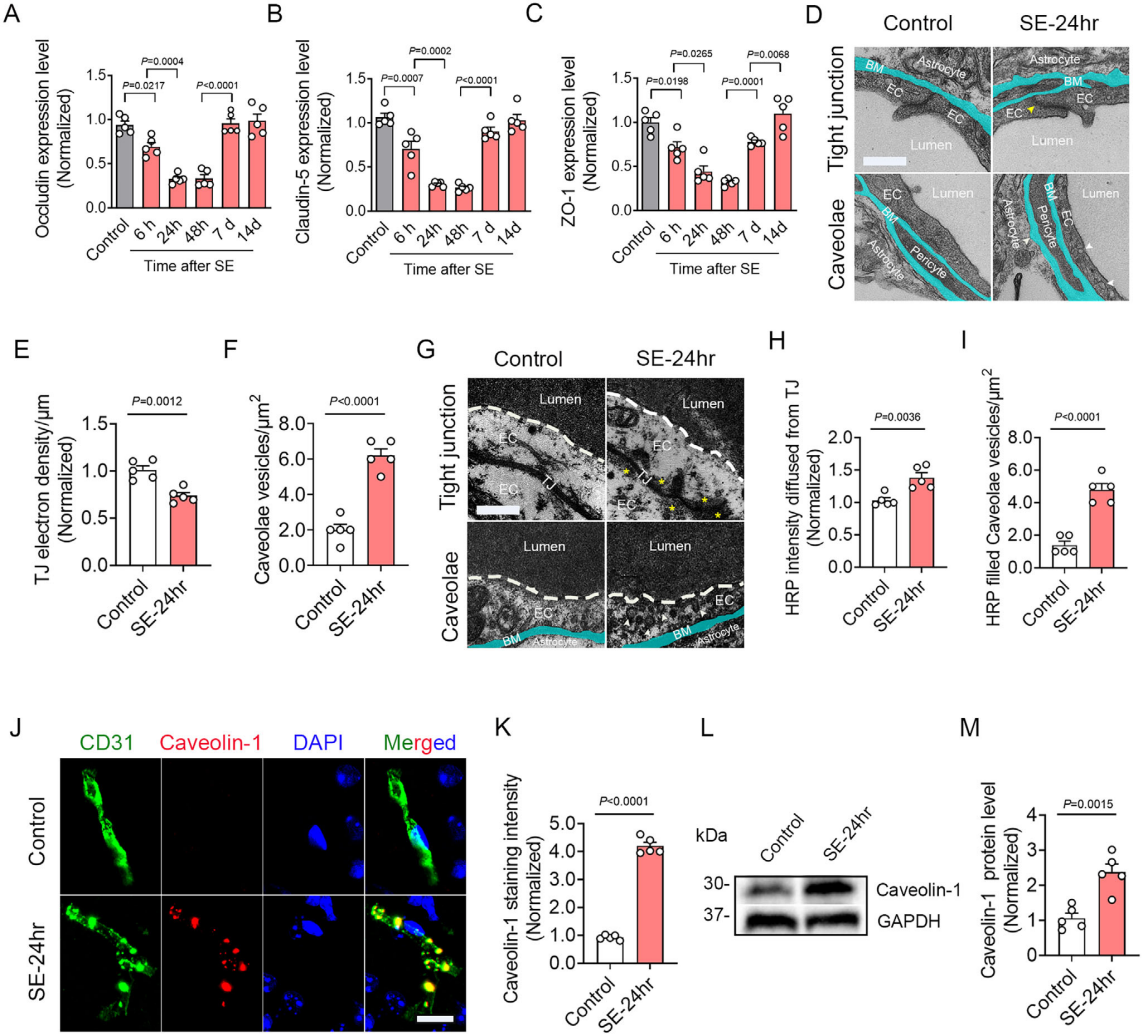
Downregulation of tight junction protein expression and increasement of caveolae formation in SE‐24 h mice. A–C) Quantitative RT‐PCR analyses of the expression of tight junction proteins of Occludin (*n =* 5; *p =* 0.0217, SE‐6 h versus Control; *p =* 0.0004, SE‐24 h versus SE‐6 h; *p <* 0.0001, SE‐7 day versus SE‐48 h), claudin‐5 (*n =* 5; *p =* 0.0007, SE‐6 h versus Control; *p =* 0.0002, SE‐24 h versus SE‐6 h; *p <* 0.0001, SE‐7 day versus SE‐48 h), and ZO‐1 (*n =* 5; *p =* 0.0198, SE‐6 h versus Control; *p =* 0.0265, SE‐24 h versus SE‐6 h; *p =* 0.0001, SE‐7 day versus SE‐48 h; *p =* 0.0068, SE‐14 day versus SE‐7 day) at different time points following SE. D) TEM examination of tight junction and caveolae of perfused mice (Yellow arrowhead indicates tight junction with decreased electron density. White arrowheads indicate caveolae vesicles). E, F) Bar graph quantifying TJ electron density (*n =* 5, *p =* 0.0012, SE‐24 h versus Control) and caveolae vesicles counts (*n =* 5, *p <* 0.0001, SE‐24 h versus Control) of Control and SE‐24 h mice. G) TEM examination of tight junction and caveolae of HRP‐injected mice (Asterisks indicate HRP reaction products diffused into endothelials. White arrowheads indicate HRP‐filled caveolae vesicles). H, I) Bar graph quantifying HRP intensity diffused from TJ (*n =* 5, *p =* 0.0036, SE‐24 h versus Control) and HRP‐filled caveolae vesicles counts (*n =* 5, *p <* 0.0001, SE‐24 h versus Control) of Control and SE‐24 h mice. J) Representative images of CD31/Caveolin‐1 co‐immunostaining in the cortex of Control and SE‐24 h mice. K) Bar graph quantifying the mean fluorescence intensity of Caveolin‐1 (*n =* 5, *p <* 0.0001, SE‐24 h versus Control). L) Western blot analyses showing Caveolin‐1 protein levels in the cortex of Control and SE‐24 h mice. M) Bar graph quantifying Caveolin‐1 protein levels, expressed as the intensity ratio of Caveolin‐1 to GAPDH (*n =* 5, *p =* 0.0015, SE‐24 h versus Control). Data are presented as means ± S.E.M. Statistical analyses were performed using one‐way ANOVA followed by Tukey's post hoc test (A–C) and unpaired two‐tailed Student's *t*‐test (E, F, H, I, K, M). Scale bar = 0.2 µm in (D, G) and 15 µm in (J).

The BBB is principally composed of cerebral microvascular endothelial cells interconnected by tight junction proteins (TJPs), including Occludin, claudin‐5, and ZO‐1, which are essential for maintaining BBB integrity. Quantitative real ‐ time PCR analyses revealed a dramatic reduction in the mRNA expression of these TJPs in the cortex at 24 and 48 h post‐SE, followed by a gradual recovery at 7 and 14 days post‐SE (**Figure** **3**A–C). Consistent with these findings, quantitative real‐time PCR analyses demonstrated similar temporal patterns for ZO‐1 and Claudin‐5 expression (Figure S1A–F, Supporting Information). To further evaluate tight junction integrity, immunofluorescence staining of Occludin was performed. Our results showed that Occludin staining intensity was remarkably reduced at 24 h post‐SE across the cortex, hippocampus, and striatum, with partial recovery by 7 days post‐SE (Figure S2A–E, Supporting Information). Ultrastructural analyses via transmission electron microscopy (TEM) corroborated these observations. At 24 h post SE, tight junctions exhibited markedly reduced electron density (Figure 3D upper panel and Figure 3E), accompanied by extensive leakage of horseradish peroxidase (HRP) tracers into endothelial cells (Figure 3G upper panel and Figure 3H), confirming structural and functional impairment of the BBB.

In addition to tight junctions, caveolae‐mediated transcytosis is also essential to BBB permeability. Our TEM results revealed a significant increase in caveolae vesicles in SE‐24 h mice (Figure 3D bottom panel and Figure 3F), many of which contained HRP reaction products (Figure 3G bottom panel and Figure 3I), suggesting elevated transcytotic activity. Furthermore, immunofluorescence and immunoblotting analyses demonstrated upregulated expression of caveolin‐1, the principal cytoskeletal protein of caveolae, in endothelial cells of SE‐24 h mice (Figure 3J–M).

Taken together, these findings suggest that BBB integrity is compromised in the early stages of epileptogenesis.

### 2.2 BMEC Tight Junction was Disrupted under Epileptic Microenvironment

**Figure 4:**
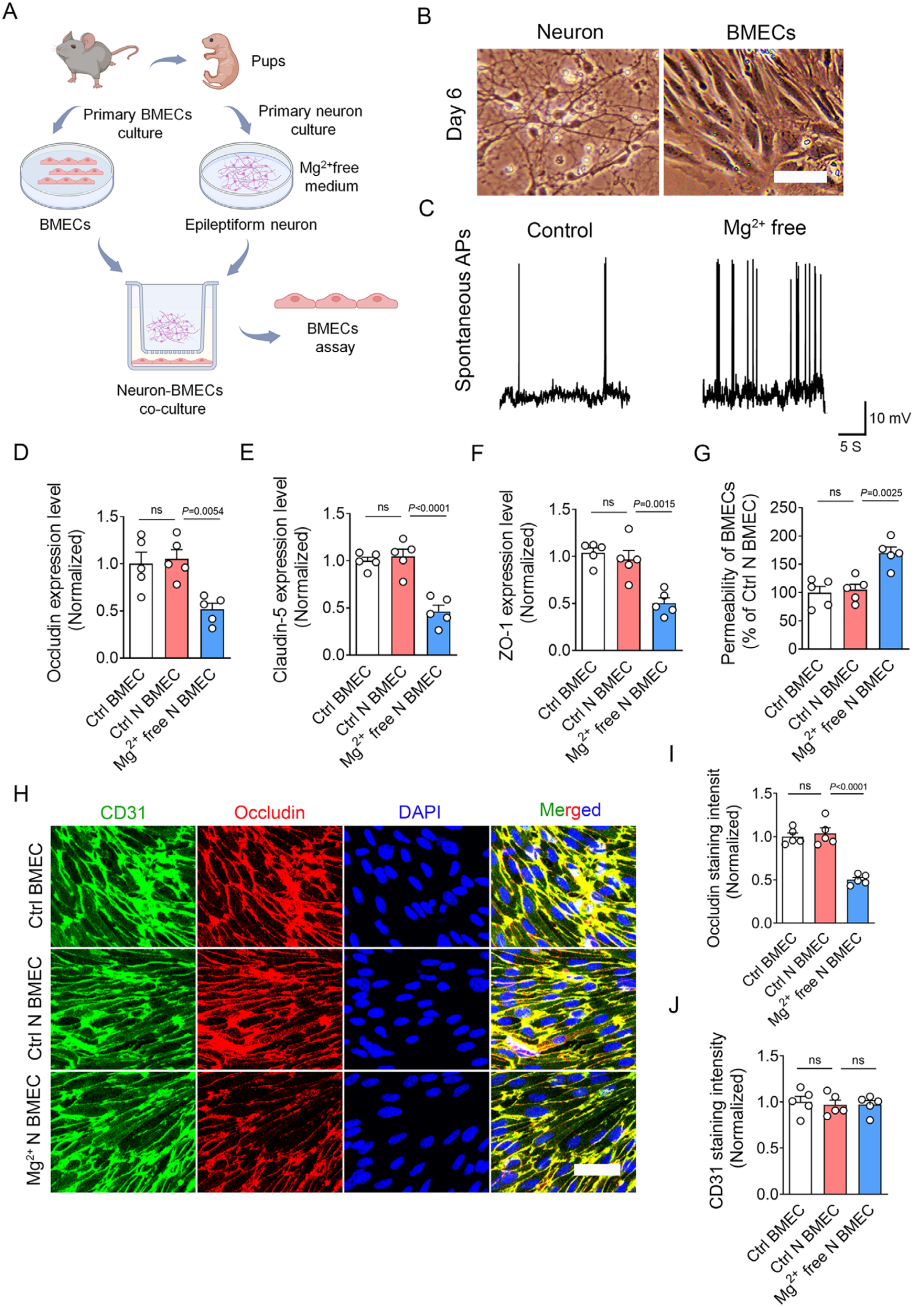
Epileptic microenvironment induces tight junction disruptions in BMECs. A) Schematic diagram illustrating the BMECs‐epileptic neuron co‐culture system, which mimics the BMECs exposed to epileptic microenvironment. BMECs are co‐cultured with primary neurons, which are induced to exhibit epileptic discharges through a magnesium (Mg^2^⁺)‐free medium. These BMECs co‐cultured with epileptic neurons are subject to tight junction integrity assay. B) Bright‐field microscopy images showing the morphology of primary BMECs and neurons at day 6 in vitro. C) Whole‐cell patch‐clamp recordings displaying the spontaneous action potential frequency in Control and Mg^2^⁺‐free medium‐treated neurons. D–F) Quantitative RT‐PCR analyses of tight junctions in Control BMECs (Ctrl BMEC), Control neuron co‐cultured BMECs (Ctrl N BMEC), and Mg^2^⁺‐free treated neuron co‐cultured BMECs (Mg^2^⁺ free N BMEC). Occludin (*n =* 5; ns, Ctrl N BMEC versus Ctrl BMEC; *p* = 0.0054, Mg^2^⁺ free N BMEC versus Ctrl N BMEC), claudin‐5 (*n =* 5; ns, Ctrl N BMEC versus Ctrl BMEC; *p <* 0.0001, Mg^2^⁺ free N BMEC versus Ctrl N BMEC), and ZO‐1 expression (*n =* 5; ns, Ctrl N BMEC versus Ctrl BMEC; *p* = 0.0015, Mg^2^⁺ free N BMEC versus Ctrl N BMEC). G) Permeability analyses using Na‐F assay in Ctrl BMEC, Ctrl N BMEC and Mg^2^^+^‐free N BMEC (*n =* 5; ns, Ctrl N BMEC versus Ctrl BMEC; *p* = 0.0025, Mg^2^⁺ free N BMEC versus Ctrl N BMEC). H) Representative images of CD31/Occludin co‐immunostaining in Ctrl BMEC, Ctrl N BMEC, and Mg^2^⁺ free N BMEC. I,J) Bar graph quantifying the mean fluorescence intensity of Occludin (*n =* 5; ns, Ctrl N BMEC versus Ctrl BMEC; *p <* 0.0001, Mg^2^⁺ free N BMEC versus Ctrl N BMEC) and CD31 (*n =* 5; ns, Ctrl N BMEC versus Ctrl BMEC; ns, Mg^2^⁺ free N BMEC versus Ctrl N BMEC) staining. Data are presented as means ± S.E.M. Statistical analyses were performed using one‐way ANOVA followed by Tukey's post hoc test. Scale bar = 25 µm.

Primary brain microvascular endothelial cells (BMECs) are widely utilized as in vitro models of the BBB. In this study, we established a BMEC‐epileptic neuron co‐culture system to simulate the BMECs in an epileptic microenvironment (**Figure** **4**A). Primary cultured neurons developed extensive dendritic arbors and formed functional synaptic connections, while BMECs formed confluent monolayers by day 6 after seeding (Figure 4B). Epileptiform activity in neurons was induced by maintaining cultures in a magnesium (Mg^2^⁺)‐free extracellular medium. The purity of the neuronal population exceeded 95%, and viability assays confirmed no significant differences between Mg^2^⁺‐free‐treated and Control neurons (data not shown). Whole‐cell patch‐clamp recordings demonstrated a significant increase in the spontaneous action potential frequency in Mg^2^⁺‐free‐treated neurons, confirming successful induction of epileptiform activity (Figure 4C). Neurotransmitter analyses revealed that glutamate release from epileptic neurons began to increase at 6 h ’ post‐treatment, peaking at 24 and 72 h (Figure S3A,B, Supporting Information).

Following co‐culture with Mg^2^⁺‐free‐treated epileptic neurons, BMECs were assessed for tight junction integrity (Figure 4A). Quantitative RT‐PCR analyses demonstrated significant downregulation of Occludin, claudin‐5 and ZO‐1 expression in BMECs co‐cultured with epileptic neurons (Mg^2^⁺ free N BMEC) compared to both BMECs co‐cultured with Control neurons (Ctrl N BMEC) and Control BMECs (Ctrl BMEC) (Figure 4D–F). Sodium fluorescein (Na‐F) permeability assays revealed markedly increased paracellular permeability in Mg^2+^ free N‐BMECs monolayers compared to Controls (Figure 4G). Immunofluorescence analyses confirmed a pronounced reduction in Occludin staining intensity in Mg^2^⁺ free N‐BMEC (Figure 4H,I), while CD31 immunostaining demonstrated comparable endothelial cell density across all groups (Figure 4H,J). To investigate whether glutamate affects BMEC tight junctions, we treated primary BMEC cultures with exogenous glutamate and performed tight junction analyses. Immunofluorescence staining revealed remarkable reduction of Occludin staining intensity in glutamate‐treated BMECs compared to vehicle Controls (Figure S3C,D, Supporting Information). Consistent with these findings, quantitative real‐time PCR analyses showed decreased expression of claudin‐5 and ZO‐1 in glutamate‐treated BMECs (Figure S3E,F, Supporting Information). Na‐F permeability assay confirmed increased BMEC monolayer permeability following glutamate treatment (Figure S3G, Supporting Information).

To further demonstrate that BMEC tight junction disruption in the epileptic microenvironment is glutamate‐dependent, we utilized glutamate pyruvate transaminase (GPT), also known as alanine aminotransferase, to degrade glutamate in the BMECs‐epileptic neuron co‐culture system. Our results showed that GPT treatment attenuated the epileptic neuron‐induced downregulation of tight junction proteins and reduced BMEC permeability (Figure S3H–K, Supporting Information). Taken together, these findings suggest that BMEC tight junction is compromised in an epileptic microenvironment, and that glutamate plays a critical role in mediating this disruption.

### 2.3 CircPTPN4 is Upregulated During Early Epileptogenesis in Temporal Lobe Epilepsy Mice

To identify potential dysregulated circRNAs in epileptic brains, we performed circRNA sequencing analyses on cortical tissue from mice 24 h after Status Epilepticus. Raw RNA sequencing data for both Control and epileptic mice were uploaded to the Gene Expression Omnibus (GEO) database, with accession number GSE273352. Bioinformatics analyses revealed that 86.2% of detected circRNAs were canonical exon‐derived circRNAs generated through back‐splicing (Figure S4A, Supporting Information). These circRNAs were further characterized by their exon composition, with single‐exon circRNAs typically longer than multiple‐exon circRNAs (Figure S4B, Supporting Information). Most circRNAs contained 2–5 exons (Figure S4C, Supporting Information), and their genomic distribution is illustrated in Figure S4D, Supporting Information. Gene Ontology (GO) annotation of the genes derived from circRNAs is presented in Figure S4E, Supporting Information.

**Figure 5:**
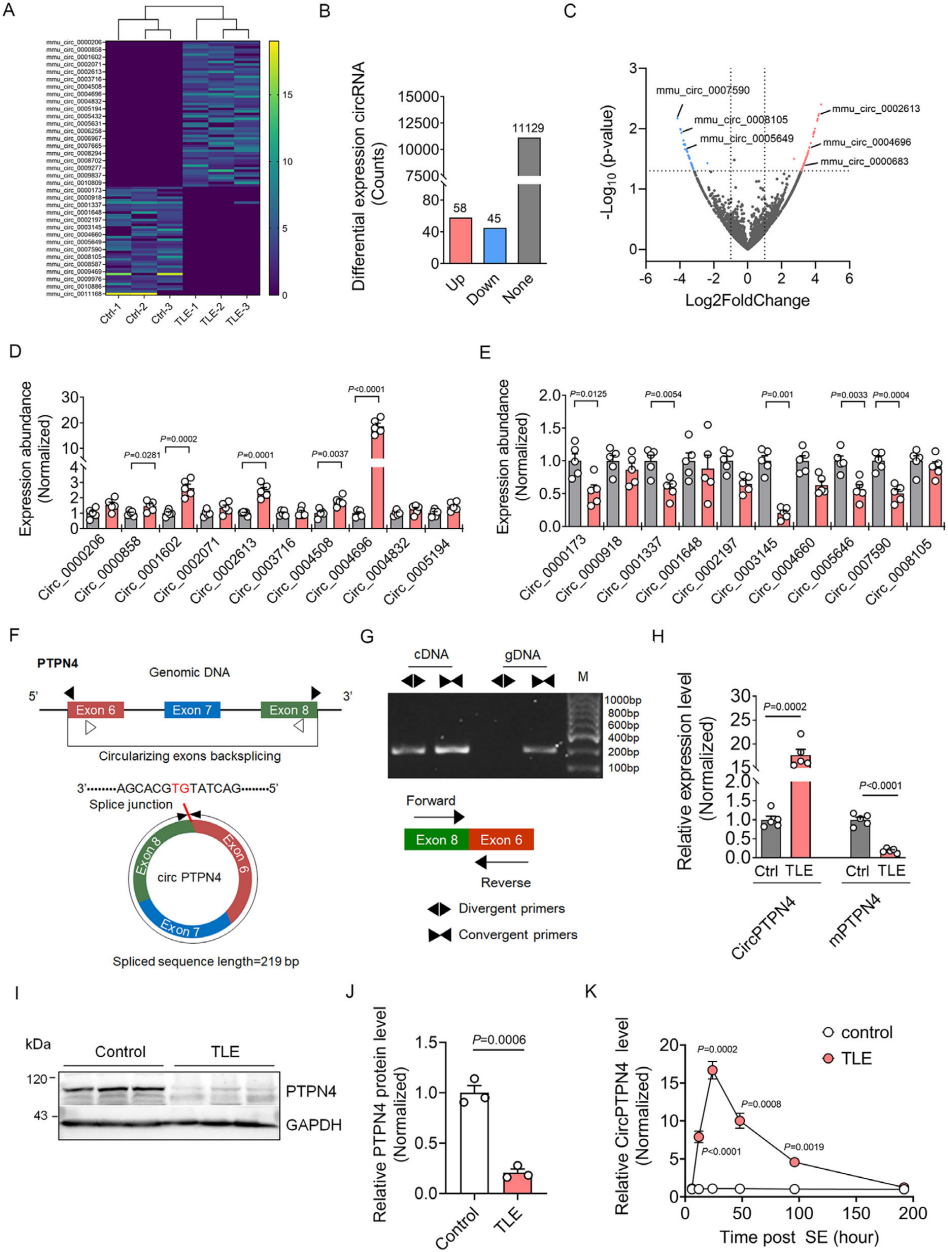
Upregulation of CircPTPN4 during early epileptogenesis in TLE mice. A) Heatmap showing hierarchical clustering of the top differentially expressed circRNA profiles in the cortex of Control and TLE mice. B) Bar graph illustrating the total number of 103 differentially expressed circRNAs, with 58 upregulated and 45 downregulated in TLE mice compared to Controls. C) Volcano plot displaying log2‐transformed fold changes in global circRNA expression in the cortex of Control and TLE mice (Orange, blue, and gray dots represent upregulated, downregulated, and unchanged circRNAs, respectively). D,E) Quantitative RT‐PCR validation of the top 10 upregulated and downregulated circRNAs in the cortex of Control and TLE mice (*n =* 5). F) Schematic diagram showing the circularization of exons 6, 7, and 8 of the host gene PTPN4 to form CircPTPN4. G) Divergent primers amplifying CircPTPN4 in cDNA, but not in genomic DNA (gDNA). H) Quantitative RT‐PCR analyses of CircPTPN4 and linear PTPN4 mRNA (mPTPN4) levels in Control and TLE mice (*n =* 5; CircPTPN4: *p =* 0.0002, TLE versus Control; mPTPN4: *p <* 0.0001, TLE versus Control). I) Western blot showing the protein level of PTPN4 in the cortex of Control and TLE mice. J) Bar graph quantifying the relative protein level of PTPN4 in the cortex of Control and TLE mice (*n =* 3, *p =* 0.0006, TLE versus Control). K) Quantification of CircPTPN4 expression levels at different time points in Control and TLE mice (*n =* 5; *p <* 0.0001 at 12 h; *p =* 0.0002 at 24 h; *p =* 0.0008 at 48 h; and *p =* 0.0019 at 96 h post‐SE). Data are presented as means ± S.E.M. Statistical significance was assessed using an unpaired two‐tailed Student's *t*‐test.

In total, 11 232 circRNAs were detected by sequencing. Among them, the expression of 103 circRNAs in the cortex of TLE mice was markedly altered compared to Control mice, with 58 circRNAs upregulated and 45 downregulated (**Figure** **5**B). Heatmap and volcano plot visualizations highlight the top upregulated and downregulated circRNAs (Figure 5A,C). These changes in circRNA expression were further validated by quantitative RT‐PCR analyses. Our data showed that the expression of the top 20 dysregulated circRNAs, including 10 upregulated and 10 downregulated circRNAs, was consistent with the sequencing results. Notably, Circ_0 004696 was the most remarkably upregulated circRNA (Figure 5D,E).

Circ_0 004696 (circBase ID: mmu_circ_0000069), hereafter termed CircPTPN4 originates from exons 6, 7, and 8 (chr1:121661607‐121662499) of the protein tyrosine phosphatase non‐receptor type 4 (PTPN4) gene (Gene ID: 19 258; NM_01 9933), with a length of 219 nucleotides (Figure 5F). cDNA and genomic DNA (gDNA) were amplified using divergent and convergent primers, respectively. Divergent primers amplified CircPTPN4 only in cDNA but not in gDNA, confirming its circular nature (Figure 5G). Circular RNAs are homologous to their corresponding messenger RNAs (mRNAs) and are primarily generated through reverse splicing of pre‐mRNA precursors. The synthesis of CircRNAs may compete with mRNA production, suggesting a regulatory interaction between these RNA species.^[^ ^34^ ^]^ An increase in circRNA production could therefore result in a relative decrease in mRNA levels.^[^ ^35^ ^]^ As expected, CircPTPN4 expression was remarkably upregulated in the cortex of TLE mice, while mPTPN4 (the mRNA of PTPN4) was downregulated (Figure 5H). Consistently, TLE mice also displayed decreased PTPN4 protein levels (Figure 5I,J).

We further examined temporal expression profile of CircPTPN4 following SE. Time‐course analyses revealed dynamic CircPTPN4 expression, peaking at 24 h post‐SE and gradually declining thereafter (Figure 5K). Taken together, these findings suggest that CircPTPN4 is transiently upregulated during early epileptogenesis in TLE mice.

### 2.4 CircPTPN4 is Specifically Upregulated in Endothelial cells under Epileptic Condition

Given that seizure‐induced BBB damage is accompanied by increased CircPTPN4 expression, we sought to investigate whether there is a connection between elevated CircPTPN4 levels and BBB injury. First, we examined CircPTPN4 expression in BMECs under epileptic conditions. Fluorescence in situ hybridization (FISH) analyses confirmed the presence of CircPTPN4 in BMECs (Figure S5A, Supporting Information). Quantitative RT‐PCR analyses revealed significant upregulation of CircPTPN4 in BMECs co‐cultured with free Mg^2^⁺‐treated epileptic neurons (Mg^2^⁺ free N BMEC) compared to both Control neuron co‐cultured BMECs (Ctrl N BMEC) and Control BMECs (Ctrl BMEC) (Figure S5B, Supporting Information). In agreement with these in vitro findings, in vivo FISH localized CircPTPN4 to cerebral vascular endothelial cells (Figure S5C, Supporting Information), and quantitative RT‐PCR demonstrated increased cortical CircPTPN4 expression in TLE mice at 24 h post‐SE (Figure S5D, Supporting Information).

To determine the cellular specificity of increase CircPTPN4 expression, we examined CircPTPN4 expression in free Mg^2^⁺‐treated neurons. While FISH analyses confirmed the presence of CircPTPN4 in neurons (Figure S6A, Supporting Information), no significant difference was observed between epileptic and Control neurons (Figure S6B, Supporting Information), suggesting endothelial‐specific upregulation of CircPTPN4 expression under epileptic conditions. We further investigated whether CircPTPN4 upregulation was specific to epileptic conditions by subjecting BMECs to oxygen‐glucose deprivation (OGD) (Figure S6C, Supporting Information). Although OGD treatment significantly reduced Occludin expression (Figure S6D,F, Supporting Information) without affecting cell viability (Figure S6E, Supporting Information), it did not alter CircPTPN4 levels (Figure S6G, Supporting Information). These findings demonstrate that while OGD can disrupt tight junctions, this occurs independently of CircPTPN4 regulation, highlighting the epilepsy‐specific nature of CircPTPN4 upregulation. Taken together, these results demonstrate that CircPTPN4 is specifically upregulated in endothelial cells under epileptic conditions, suggesting a potential role in epilepsy‐associated BBB dysfunction.

### 2.5 Knockdown of CircPTPN4 Ameliorates BBB Damage and Decreases Spontaneous Seizure Frequency Following SE

**Figure 6:**
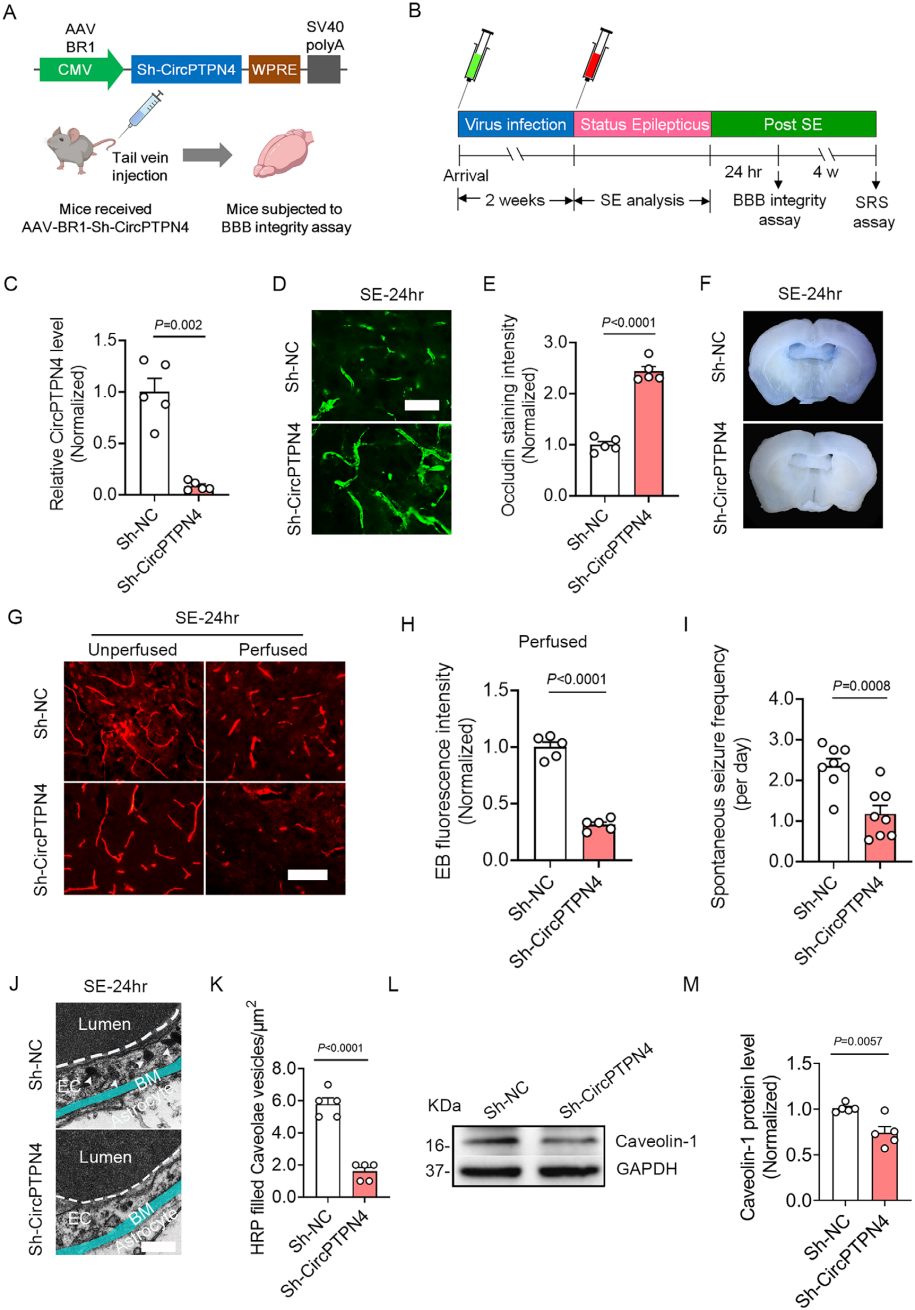
Knockdown of CircPTPN4 ameliorates BBB damage and reduces spontaneous seizure frequency following SE. A) Schematic diagram depicting AAV constructs for CircPTPN4‐targeting shRNA (Sh‐CircPTPN4), where the short hairpin RNA is fused with the brain endothelium‐targeting peptide NRGTEWD (BR1) and controlled by a CMV promoter. B) Experimental design schematic. 2 weeks after viral injection, mice underwent SE induction followed by BBB integrity and SRS frequency assays at 24 h and 4 weeks post‐SE, respectively. C) Quantitative RT‐PCR analyses validating CircPTPN4 knockdown efficiency in the cortex of Sh‐NC‐ and Sh‐CircPTPN4‐treated SE mice (*n =* 5, *p =* 0.002, Sh‐CircPTPN4 versus Sh‐NC). D) Representative images of Occludin immunostaining in the cortex of Sh‐NC‐ and Sh‐CircPTPN4‐treated SE mice. E) Bar graph quantifying the mean fluorescence intensity of Occludin staining (*n =* 5, *p <* 0.0001, Sh‐CircPTPN4 versus Sh‐NC). F) Coronal slices showing extravasated EB staining in the parenchyma of SE‐24 h mice treated with Sh‐NC or Sh‐CircPTPN4. G) EB fluorescence detected in the cortex of Sh‐CircPTPN4‐ and Sh‐NC‐treated mice 24 h after SE, before and after perfusion. H) Bar graph quantifying the mean fluorescence intensity of EB in the cortex of Sh‐CircPTPN4‐ and Sh‐NC‐treated perfused mice (*n =* 5, *p <* 0.0001, Sh‐CircPTPN4 versus Sh‐NC). I) Bar graph quantifying SRS frequency in Sh‐CircPTPN4‐ and Sh‐NC‐treated mice 4 weeks after SE (*n =* 8, *p =* 0.0008, Sh‐CircPTPN4 versus Sh‐NC). J) TEM examination of caveolae of HRP‐injected Sh‐NC‐ and Sh‐CircPTPN4‐treated SE mice (White arrowheads indicate HRP‐filled caveolae vesicles). K) Bar graph quantifying HRP filled caveolae vesicles counts (*n =* 5, *p <* 0.0001, Sh‐CircPTPN4 versus Sh‐NC). L) Western blot analyses showing Caveolin‐1 protein levels in the cortex of Sh‐NC‐ and Sh‐CircPTPN4‐treated SE mice. M) Bar graph quantifying Caveolin‐1 protein levels, expressed as the intensity ratio of Caveolin‐1 to GAPDH (*n =* 5, *p =* 0.0057, Sh‐CircPTPN4 versus Sh‐NC). Data are presented as means ± S.E.M. Statistical analyses was performed using an unpaired two‐tailed Student's *t*‐test (E, H, I, K, M), an unpaired two‐tailed Welch's *t*‐test (C). Scale bars = 25 µm in (D, G), 0.2 µm in (J).

To investigate the role of CircPTPN4 in BBB disruption following epilepsy, mice were administered a CircPTPN4‐targeting shRNA (Sh‐CircPTPN4) that includes a brain‐ endothelium targeting peptide (NRGTEWD, BR1)^[^ ^36^ ^]^ and a CMV promoter via tail vein injection (**Figure** **6**A). After 2 weeks of infection, Status Epilepticus (SE) was induced in the mice, followed by BBB integrity assays 24 h post‐SE and a spontaneous recurrent seizure (SRS) assay 4 weeks after SE (Figure 6B). CircPTPN4 knockdown efficiency was confirmed by significantly reduced CircPTPN4 mRNA levels in the cortex (Figure 6C), hippocampus (Figure S7A, Supporting Information), and striatum (Figure S7B, Supporting Information).

Immunofluorescence analyses 24 h post‐SE revealed markedly increased Occludin staining intensity in Sh‐CircPTPN4‐treated mice compared to Sh‐NC Controls within the cortex (Figure 6D,E), hippocampus (Figure S7C,D, Supporting Information), and striatum (Figure S7E,F, Supporting Information). EB extravasation assays demonstrated decreased EB leakage in the parenchyma and ventricular regions following CircPTPN4 knockdown (Figure 6F). Pre‐perfusion EB fluorescence intensity showed no significant difference between Sh‐CircPTPN4‐ and Sh‐NC‐treated groups across brain regions. However, post‐perfusion analyses indicated lower EB fluorescence intensity in the parenchyma of the cortex (Figure 6G,H), hippocampus (Figure S7G,H, Supporting Information), and striatum (Figure S7I,J, Supporting Information) in Sh‐CircPTPN4‐treated mice compared to Sh‐NC‐treated mice 24 h post‐SE. Furthermore, spontaneous seizure frequency assay revealed that knockdown of CircPTPN4 reduced spontaneous seizure frequency 4 weeks after SE (Figure 6I). TEM results demonstrated a marked reduction in HRP‐filled caveolae vesicles within endothelial cells of Sh‐CircPTPN4‐treated SE‐24 h mice compared to Sh‐NC Controls (Figure 6J,K), indicating diminished transcytotic activity. Furthermore, immunoblotting analyses demonstrated decreased expression of caveolin‐1 in endothelial cells of Sh‐CircPTPN4‐treated SE‐24 h mice compared to Sh‐NC Controls (Figure 6L,M).

To assess the effect of CircPTPN4 on BMEC tight junctions under epileptic conditions, BMECs were transduced with lentivirus carrying Sh‐CircPTPN4 and co‐cultured with free Mg^2^⁺‐treated neurons (Figure S8A, Supporting Information). Quantitative real‐time PCR confirmed the efficient knockdown of CircPTPN4 in BMECs (Figure S8B, Supporting Information). Immunofluorescence analyses revealed that Occludin staining intensity was remarkably increased in Sh‐CircPTPN4‐treated BMECs compared to Sh‐NC Controls under epileptic conditions, while CD31 staining remained unchanged (Figure S8C–E, Supporting Information). Additionally, permeability assay confirmed reduced permeability of Sh‐CircPTPN4‐treated BMECs compared to Sh‐NC Controls (Figure S8F, Supporting Information).

To further investigate the effect of CircPTPN4 on BMEC tight junctions under basal conditions, CircPTPN4 was overexpressed (OE‐CircPTPN4) or knocked down (Sh‐CircPTPN4) in primary BMECs (Figure S9A, Supporting Information). The efficiency of CircPTPN4 overexpression and knockdown was verified in Figure S9B, Supporting Information. Quantitative RT‐PCR analyses revealed that CircPTPN4 overexpression suppressed Occludin expression (Figure S9C, Supporting Information). Consistently, immunofluorescence analyses showed that Occludin staining intensity was markedly decreased in CircPTPN4‐overexpressing (OE‐CircPTPN4) BMECs compared to Controls (Figure S9D–F, Supporting Information). Furthermore, OE‐CircPTPN4‐treated BMECs exhibited increased permeability compared to both Sh‐CircPTPN4‐treated and Control BMECs (Figure S9G, Supporting Information).

Taken together, these results suggest that CircPTPN4 knockdown ameliorates BBB damage and reduces SRS following epilepsy, highlighting the potential role of CircPTPN4 in modulating BBB integrity during epileptogenesis.

### 2.6 CircPTPN4 Promotes ECE‐1 Expression by Sponging miR‐145a‐5p

CircRNAs have been recognized as competing endogenous RNAs (CeRNAs) that sponge microRNAs (miRNAs), thereby disinhibiting the expression of specific target genes in a competitive manner. To identify the potential miRNAs targeted by CircPTPN4, we analyzed CircInteractome databases and predicted five candidate miRNAs: miR‐127‐5p, miR‐1305, miR‐145a‐5p, miR‐186, and miR‐431. We then analyzed the expression patterns of these miRNAs in the cortex of TLE mice 24 h post‐SE. Among the five predicted miRNAs, only miR‐145a‐5p revealed a significant alteration in expression (Figure S10A, Supporting Information).

To validate the expression of miR‐145a‐5p in BMECs under epileptic conditions, BMECs were co‐cultured with Mg^2+^ free neurons prior to miR‐145a‐5p analyses (Figure S10B, Supporting Information). Given previous reports of neuronal miR‐145a‐5p expression,^[^ ^37^ ^]^ FISH assay confirmed the location of miR‐145a‐5p in neurons (Figure S10C, Supporting Information). Quantitative RT‐PCR results indicated no difference in miR‐145a‐5p expression between Mg^2^⁺‐ free‐treated neurons and Controls (Figure S10D, Supporting Information). However, miR‐145a‐5p expression was markedly downregulated in BMECs co‐cultured with Mg^2^⁺‐ free‐treated neurons versus Control BMECs or BMECs co‐cultured with untreated neurons (Figure S10E, Supporting Information), confirming specific BMECs downregulation of miR‐145a‐5p under epileptic conditions. In vivo FISH detected decreased expression of miR‐145a‐5p in endothelial cells (Figure S10F, Supporting Information), and quantitative RT‐PCR showed decreased expression of miR‐145a‐5p at 24 h post‐SE with recovery observed by day 7 (Figure S10G, Supporting Information).

**Figure 7:**
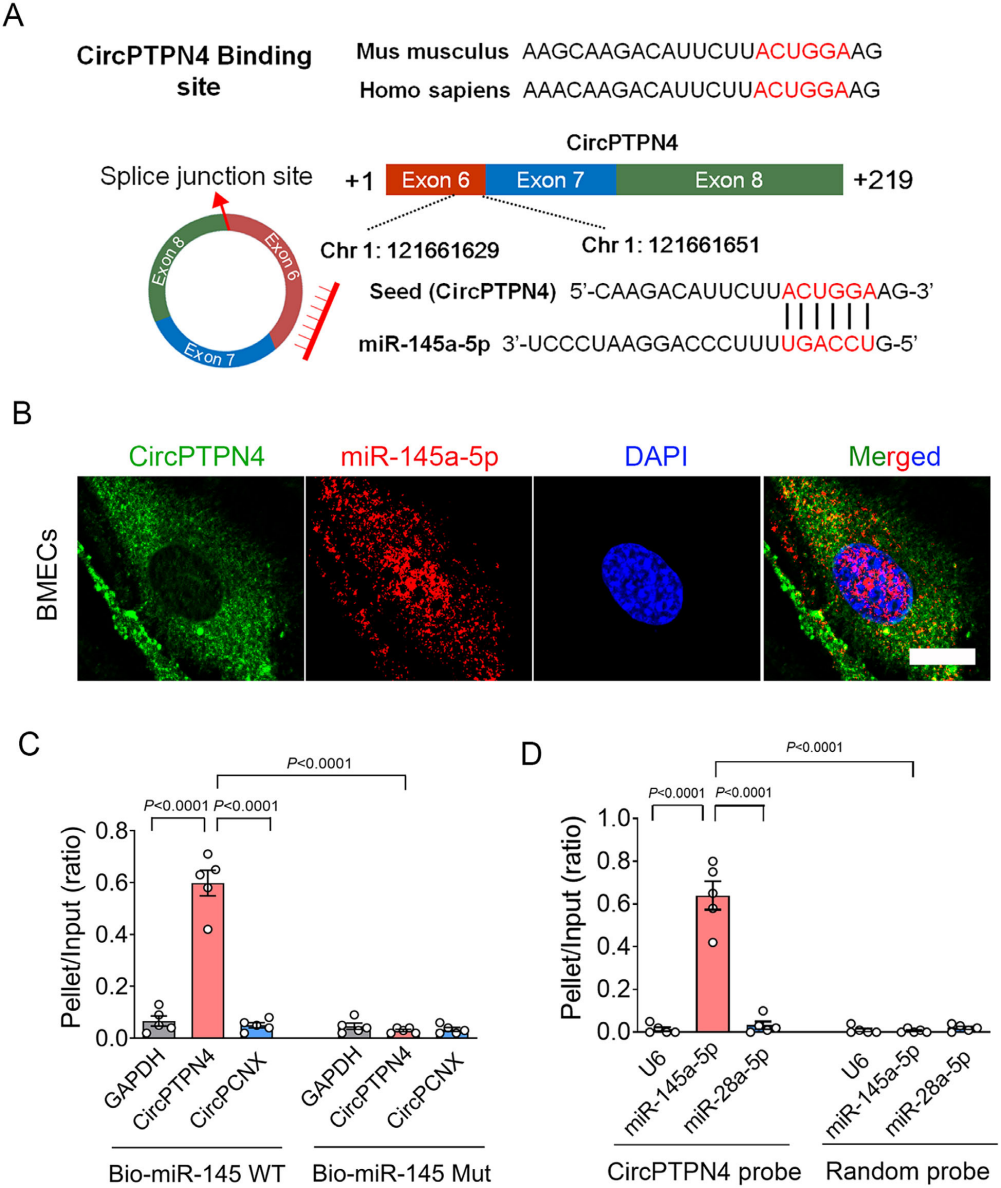
CircPTPN4 functions as a molecular sponge for miR‐145a‐5p. A) Schematic representation of the binding site in the CircPTPN4 seed region for miR‐145a‐5p. B) FISH assays showing the location of CircPTPN4 and miR‐145a‐5p in primary BMECs. C) Quantitative RT‐PCR analyses of CircPTPN4, GAPDH, and CircPCNX RNAs pulled down by biotinylated WT miR‐145a‐5p (Bio‐miR‐145 WT) and biotinylated mutant miR‐145a‐5p (Bio‐miR‐145 Mut), respectively (*n =* 5; *p <* 0.0001, Bio‐miR‐145 WT‐pulled down CircPTPN4 versus GAPDH; *p <* 0.0001, Bio‐miR‐145 WT‐pulled down CircPCNX versus CircPTPN4; *p <* 0.0001, Bio‐miR‐145 Mut‐pulled down CircPTPN4 versus Bio‐miR‐145 WT‐pulled down CircPTPN4). D) RT‐PCR analyses of U6, miR‐145a‐5p, and miR‐28a‐5p RNAs pulled down by CircPTPN4 and random probe, respectively (*n =* 5; *p <* 0.0001, CircPTPN4 probe‐pulled down miR‐145a‐5p versus U6; *p <* 0.0001, CircPTPN4 probe‐pulled down miR‐28a‐5p versus miR‐145a‐5p; *p <* 0.0001, CircPTPN4 probe‐pulled down miR‐145a‐5p versus random probe‐pulled down miR‐145a‐5p). Data are presented as means ± S.E.M. Statistical analysis was performed using two‐way ANOVA followed by Tukey's test. Scale bar = 10 µm.

The predicted binding site of the CircPTPN4 seed region (Exon6; chr1:121661629‐chr1:121 661 651) to miR‐145a‐5p is shown in **Figure** **7**A. FISH analyses confirmed cytoplasmic co‐localization of CircPTPN4 and miR‐145a‐5p in BMECs (Figure 7B), supporting a potential ceRNA interaction. Biotinylated miR‐145a‐5p probe pulldown assays demonstrated significant enrichment of CircPTPN4 in complexes captured by the wild‐type (WT), but not a mutant, miR‐145a‐5p probe (Figure 7C). CircPCNX was undetectable in the miR‐145a‐5p‐captured fraction, further supporting the specificity of this interaction. Reciprocally, biotinylated CircPTPN4 pulled down miR‐145a‐5p, but not miR‐28a‐5p, and a random probe failed to pull down miR‐145a‐5p (Figure 7D).

Next, we identified the potential target genes of miR‐145a‐5p by performing bioinformatics analyses using miRDB, Starbase, and TargetScan, which identified 195 target genes (Figure S12A, Supporting Information). KEGG pathway analyses indicated that these target genes were largely correlated with endocytosis, MAPK signaling, axon guidance, and tight junction regulation (Figure S12B, Supporting Information). When focusing on tight junction‐associated genes, we identified ECE‐1, a rate‐limiting enzyme responsible for endothelin‐1 (ET‐1) production, as a key target. TargetScan predicted a miR‐145a‐5p binding site in the ECE‐1 3′‐UTR (Chr4:137589927‐Chr4:137 589 932) (Figure S11A, Supporting Information). Luciferase reporter assays confirmed direct binding, as miR‐145a‐5p mimics suppressed luciferase activity driven by the WT, but not a mutant, to ECE‐1 3′ UTR (Figure S11B, Supporting Information).

Assessment of ECE‐1 expression under epileptic conditions revealed upregulated mRNA and protein levels in the TLE mouse cortex 24 h post‐SE, returning to baseline by day 7 (Figure S11C–E, Supporting Information). Immunofluorescence showed that ECE‐1 was primarily localized to the vasculature (co‐localized with CD31), with markedly increased staining intensity at 24 h post‐SE and decreased expression by day 7 post SE (Figure S12C–E, Supporting Information). In vitro, ECE‐1 expression was remarkably upregulated in BMECs co‐cultured with Mg^2^⁺ free‐treated neurons compared to Controls (Figure S11F–H, Supporting Information), with immunofluorescence confirming endothelial localization (CD31 co‐localization) and increased staining intensity (Figure S11I,J, Supporting Information).

**Figure 8:**
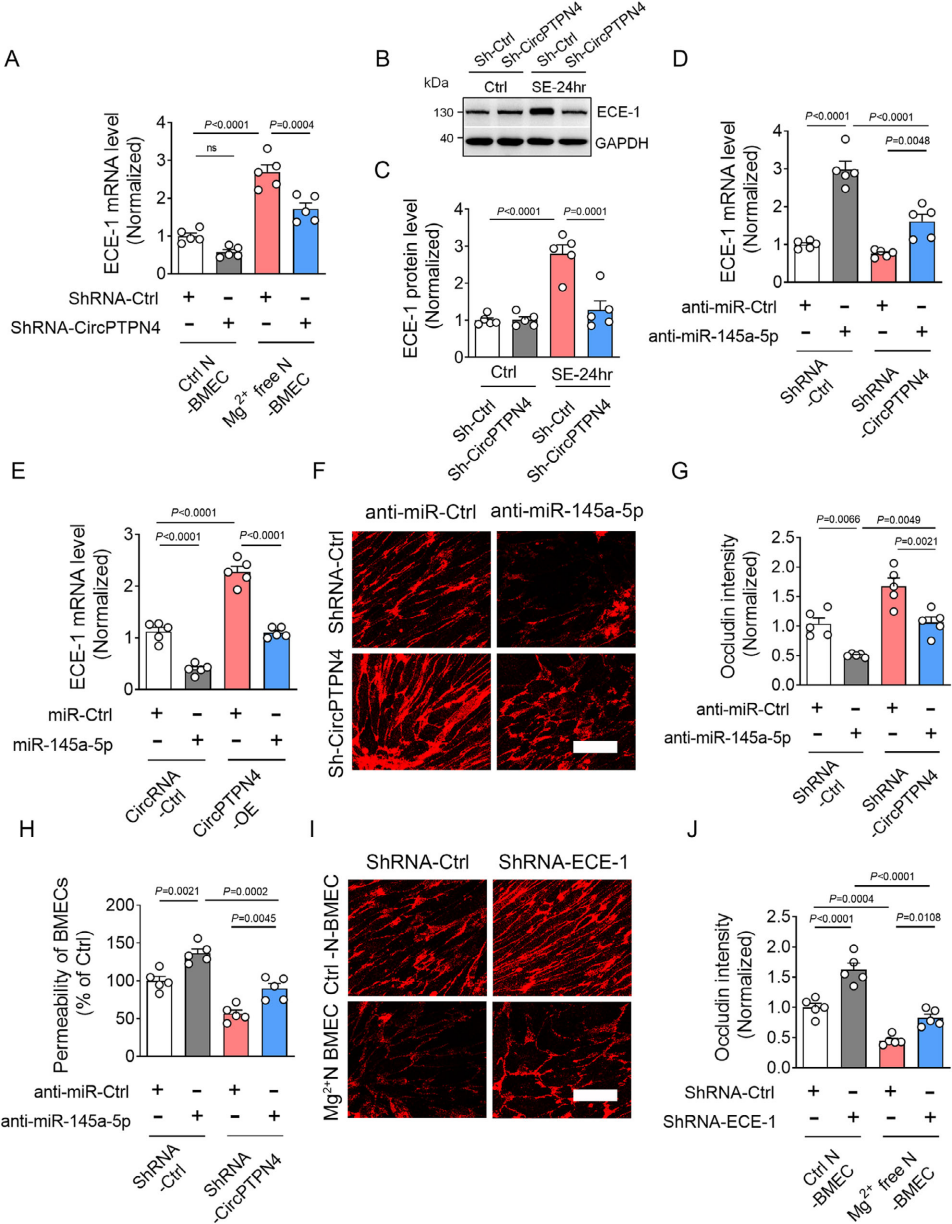
CircPTPN4 disrupts tight junction by upregulating ECE‐1 expression via miR‐145a‐5p sponging in BMECs. A) Quantitative RT‐PCR analyses of ECE‐1 mRNA levels in BMECs co‐cultured with Control neurons or Mg^2^⁺‐free neurons, and transduced with either Control‐ShRNA or CircPTPN4‐ShRNA lentivirus (*n =* 5; *p <* 0.0001, ShRNA‐Ctrl‐treated Mg^2^⁺‐free N BMECs versus ShRNA‐Ctrl‐treated Ctrl N BMECs; *p =* 0.0004, ShRNA‐CircPTPN4‐treated Mg^2^⁺‐free N BMECs versus ShRNA‐Ctrl‐treated Mg^2^⁺‐free N BMECs). B) Western blot analyses of ECE‐1 protein levels in the cortex of Control and SE‐24 h mice infected with Control‐ShRNA or CircPTPN4‐ShRNA lentivirus. C) Bar graph quantifying ECE‐1 protein levels as the intensity ratio of ECE‐1 to GAPDH (*n =* 5; *p <* 0.0001, ShRNA‐Ctrl‐treated SE‐24 h mice versus ShRNA‐Ctrl‐treated Ctrl mice; *p <* 0.0001, ShRNA‐CircPTPN4‐treated SE‐24 h mice versus ShRNA‐Ctrl‐treated SE‐24 h mice). D) Quantitative RT‐PCR analyses of ECE‐1 mRNA expression in Mg^2+^ free N BMECs transduced with ShRNA‐Ctrl or ShRNA‐CircPTPN4 lentivirus, and co‐treated with anti‐miR Control or miR‐145a‐5p inhibitors (*n =* 5; *p <* 0.0001, anti‐miR‐145a‐5p and ShRNA‐Ctrl BMECs versus Control anti‐miRNA and ShRNA‐Ctrl BMECs; *p =* 0.0048, anti‐miR‐145a‐5p and ShRNA‐CircPTPN4 BMECs versus Control anti‐miRNA and ShRNA‐CircPTPN4 BMECs; *p <* 0.0001, anti‐miR‐145a‐5p and ShRNA‐CircPTPN4 BMECs versus anti‐miR‐145a‐5p and ShRNA‐Ctrl BMECs). E) Quantitative RT‐PCR analyses of ECE‐1 mRNA expression in Mg^2+^ free N BMECs infected with OE‐Ctrl or OE‐CircPTPN4 lentivirus, and co‐treated with miR Control or miR‐145a‐5p mimics (*n =* 5; *p <* 0.0001, miR Control and OE‐Ctrl BMECs versus miR‐145a‐5p mimics and OE‐Ctrl BMECs; *p <* 0.0001, miR Control and OE‐Ctrl BMECs versus miR Control and OE‐CircPTPN4 BMECs; *p <* 0.0001, miR Control and OE‐CircPTPN4 BMECs versus miR‐145a‐5p mimics and OE‐CircPTPN4 BMECs). F) Representative immunofluorescence images of Occludin staining in Mg^2+^ free N BMECs infected with ShRNA‐Ctrl or ShRNA‐CircPTPN4 lentivirus, and co‐infected with anti‐miR Control or miR‐145a‐5p inhibitors. G) Bar graph showing quantification of Occludin staining intensity in Mg^2+^ free N BMECs infected with ShRNA‐Ctrl or ShRNA‐CircPTPN4 lentivirus, and co‐infected with anti‐miR Control or miR‐145a‐5p inhibitors (*n =* 5; *p =* 0.0066, anti‐miR‐145a‐5p and ShRNA‐Ctrl BMECs versus Control anti‐miRNA and ShRNA‐Ctrl BMECs; *p =* 0.0021, anti‐miR‐145a‐5p and ShRNA‐CircPTPN4 BMECs versus Control anti‐miRNA and ShRNA‐CircPTPN4 BMECs; *p =* 0.0049, anti‐miR‐145a‐5p and ShRNA‐CircPTPN4 BMECs versus anti‐miR‐145a‐5p and ShRNA‐Ctrl BMECs). H) Bar graph showing the permeability of Mg^2+^ free N BMECs transfected with ShRNA‐Ctrl or ShRNA‐CircPTPN4 lentivirus, and co‐treated with anti‐miR Control or miR‐145a‐5p inhibitors (*n =* 5; *p =* 0.0021, anti‐miR‐145a‐5p and ShRNA‐Ctrl BMECs versus Control anti‐miRNA and ShRNA‐Ctrl BMECs; *p =* 0.0045, anti‐miR‐145a‐5p and ShRNA‐CircPTPN4 BMECs versus Control anti‐miRNA and ShRNA‐CircPTPN4 BMECs; *p =* 0.0002, anti‐miR‐145a‐5p and ShRNA‐CircPTPN4 BMECs versus anti‐miR‐145a‐5p and ShRNA‐Ctrl BMECs). I) Representative immunofluorescence images of Occludin staining in Mg^2+^ free N BMECs co‐cultured with Control or Mg^2^⁺‐free neurons, and infected with Control‐ShRNA or ECE‐1‐ShRNA lentivirus. J) Bar graph showing quantification of Occludin staining in Ctrl N BMECs or Mg^2^⁺‐free N BMECs, and infected with Control‐ShRNA or ECE‐1‐ShRNA lentivirus (*n =* 5; *p <* 0.0001, ShRNA‐ECE‐1‐treated Ctrl N BMECs versus ShRNA‐Ctrl‐treated Ctrl N BMECs; *p =0*.0108, ShRNA‐ECE‐1‐treated Mg^2^⁺‐free N BMECs versus ShRNA‐Ctrl‐treated Mg^2^⁺‐free N BMECs; *p =* 0.0004, ShRNA‐Ctrl‐treated Mg^2^⁺‐free N BMECs versus ShRNA‐Ctrl‐treated Ctrl N BMECs; *p <* 0.0001, ShRNA‐ECE‐1‐treated Mg^2^⁺‐free N BMECs versus ShRNA‐ECE‐1‐treated Ctrl N BMECs). Values represent means ± S.E.M. Statistical analysis were performed using one‐way ANOVA followed by Tukey's test. Scale bar = 25 µm.

Next, to investigate the regulatory effect of miR‐145a‐5p on ECE‐1, BMECs were transfected with miR‐145a‐5p mimics or miR‐145a‐5p inhibitors. Transfection of BMECs with miR‐145a‐5p mimics downregulated ECE‐1 expression, while anti‐miR‐145a‐5p upregulated it, both in Control and Mg^2^⁺‐free‐treated conditions (Figure S11K,L, Supporting Information), confirming miR‐145a‐5p‐mediated inhibition of ECE‐1 expression. Furthermore, Transduction of CircPTPN4 shRNA lentivirus significantly attenuated the Mg^2^⁺‐free‐induced upregulation of ECE‐1 mRNA in BMECs (**Figure** **8**A). In vivo, CircPTPN4 shRNA AAV delivery inhibited seizure‐induced ECE‐1 protein upregulation in the cortex 24 h post‐SE (Figure 8B,C).

To determine if miR‐145a‐5p mediates CircPTPN4's regulation of ECE‐1, BMECs were co‐transduced with CircPTPN4 shRNA and miR‐145a‐5p inhibitors. CircPTPN4 knockdown attenuated the ECE‐1 upregulation induced by anti‐miR‐145a‐5p (Figure 8D). Conversely, overexpressing CircPTPN4 (OE‐CircPTPN4) increased ECE‐1, an effect suppressed by co‐treatment with miR‐145a‐5p mimics (Figure 8E), confirming miR‐145a‐5p's role in this regulatory pathway.

Taken together, these findings suggest that CircPTPN4 regulates ECE‐1 expression by binding to miR‐145a‐5p, highlighting a novel molecular pathway involved in BBB disruption following epilepsy.

### 2.7 CircPTPN4 Impairs Tight Junction by Upregulating ECE‐1 Expression via Sponging miR‐145a‐5p in BMECs

Having established the CircPTPN4/miR‐145a‐5p/ECE‐1 regulatory axis, we next investigated whether CircPTPN4 compromises tight junction integrity in BMECs via miR‐145a‐5p‐mediated ECE‐1 upregulation. To test this, BMECs co‐cultured with Mg^2^⁺‐free‐treated neurons were co‐transduced with CircPTPN4 shRNA and miR‐145a‐5p inhibitors. Quantitative RT‐PCR analyses revealed that CircPTPN4 knockdown significantly increased Occludin, claudin‐5, and ZO‐1 expression. Critically, this effect was abolished in BMECs co‐transduced with anti‐miR‐145a‐5p (Figure S13A–C, Supporting Information).

Consistently, immunofluorescence and permeability assays demonstrated CircPTPN4 shRNA transduction enhanced Occludin staining intensity and reduced permeability, while anti‐miR‐145a‐5p reversed these effects (Figure 8F–H). Furthermore, ECE‐1 knockdown rescued the downregulation of Occludin, claudin‐5, and ZO‐1 expression in BMECs under epileptic conditions (Figure S13D–F, Supporting Information). Immunofluorescence analyses confirmed that ECE‐1 silencing attenuated the reduction in Occludin staining intensity in Mg^2^⁺‐free ‐treated co‐cultures (Figure 8I,J).

Taken together, these results suggest that CircPTPN4 disrupts tight junction integrity by upregulating ECE‐1 expression via miR‐145a‐5p sequestering, thereby impairing BBB function.

### 2.8 Knockdown of ECE‐1 Ameliorates BBB Damage and Decreases Spontaneous Seizure Frequency Following SE

**Figure 9:**
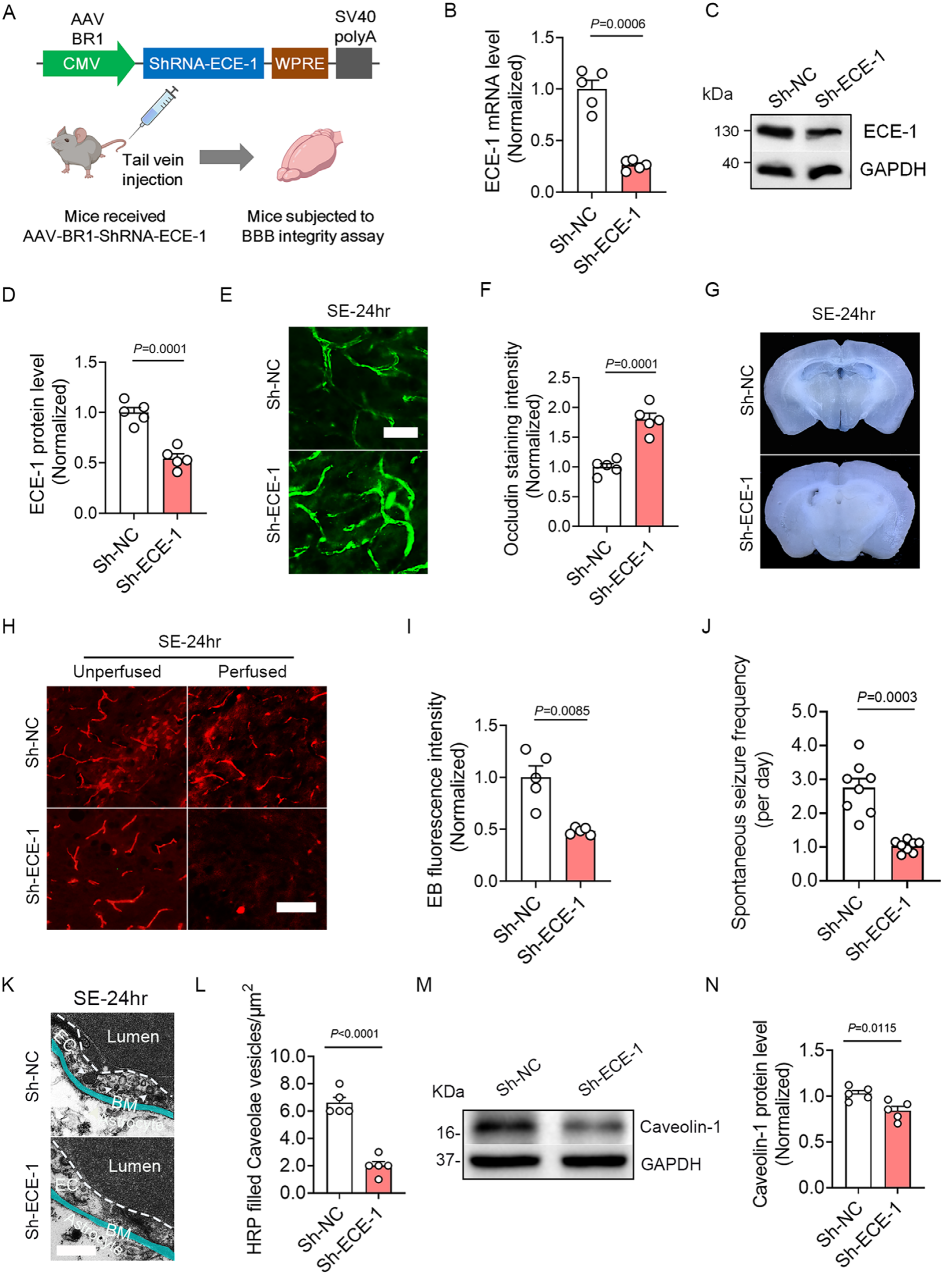
Knockdown of ECE‐1 ameliorates BBB damage in the cortex and reduces spontaneous seizure frequency following SE. A) Schematic representation of AAV constructs for endothelium ECE‐1‐targeting ShRNA (Sh‐ECE‐1). B) Quantitative RT‐PCR analyses of ECE‐1 mRNA levels in the cortex of Sh‐NC‐ and Sh‐ECE‐1‐treated SE‐24 h mice (*n =* 5, *p =* 0.0006, Sh‐ECE‐1 versus Sh‐NC group). C) Western blot analyses showing ECE‐1 protein levels in the cortex of Sh‐NC‐ and Sh‐ECE‐1‐treated SE‐24 h mice. D) Bar graph quantifying ECE‐1 protein levels, expressed as the intensity ratio of ECE‐1 to GAPDH (*n =* 5, *p =* 0.0001, Sh‐ECE‐1 versus Sh‐NC group). E) Representative images of Occludin immunostaining in the cortex of Sh‐NC‐ and Sh‐ECE‐1‐treated SE‐24 h mice. F) Bar graph showing quantification of mean fluorescence intensity of Occludin staining (*n =* 5, *p =* 0.0001, Sh‐ECE‐1 versus Sh‐NC group). G) Coronal slices showing extravasated EB staining in the parenchyma of SE‐24 h mice treated with Sh‐NC and Sh‐ECE‐1. H) EB fluorescence detected in the cortex of Sh‐ECE‐1‐ and Sh‐NC‐treated SE‐24 h mice before and after perfusion. I) Bar graph showing the quantification of the mean fluorescence intensity of EB in the cortex of Sh‐ECE‐1‐ and Sh‐NC‐treated perfused SE‐24 h mice (*n =* 5, *p =* 0.0085, Sh‐ECE‐1 versus Sh‐NC group). J) Bar graph showing the quantification of SRS in Sh‐ECE‐1‐ and Sh‐NC‐treated mice 4 weeks after SE (*n =* 8, *p =* 0.0003, Sh‐ECE‐1 versus Sh‐NC group). K) TEM examination of caveolae of HRP‐injected Sh‐NC‐ and Sh‐ECE‐1‐treated SE mice (White arrowheads indicate HRP‐filled caveolae vesicles). L) Bar graph quantifying HRP‐filled caveolae vesicles counts (*n =* 5, *p <* 0.0001, Sh‐ECE‐1 versus Sh‐NC). M) Western blot analyses showing Caveolin‐1 protein levels in the cortex of Sh‐NC‐ and Sh‐ECE‐1‐treated SE mice. N) Bar graph quantifying Caveolin‐1 protein levels, expressed as the intensity ratio of Caveolin‐1 to GAPDH (*n =* 5, *p =* 0.0115, Sh‐ECE‐1 versus Sh‐NC). Values represent means ± S.E.M. Statistical analysis were performed using an unpaired two‐tailed Student's *t*‐test (D, F, L, N) and unpaired two‐tailed Welch's *t*‐test (B, I, J). Scale bar = 25 µm in (E), 50 µm in (H) and 0.2 µm in (K).

To evaluate ECE‐1′s contribution to BBB damage following epilepsy, mice received tail vein injections of Sh‐ECE‐1 virus to specifically knock down ECE‐1 in cerebral endothelial cells (**Figure** **9**A). 2 weeks post‐injection, SE was induced, followed by BBB integrity assay 24 h later. ECE‐1 knockdown efficiency was confirmed by significantly reduced mRNA and protein levels in the cortex (Figure 9B–D), hippocampus (Figure S14A, Supporting Information), and striatum (Figure S14B, Supporting Information) of Sh‐ECE‐1‐treated mice versus Sh‐NC Controls.

Consistently, immunofluorescence analyses revealed significantly enhanced Occludin staining intensity in Sh‐ECE‐1 mice 24 h post‐SE in the cortex (Figure 9E,F), hippocampus (Figure S14C,D, Supporting Information), and striatum (Figure S14E,F, Supporting Information). EB extravasation assays further demonstrated that ECE‐1 knockdown attenuated parenchymal EB leakage (Figure 9G). Pre‐perfusion EB fluorescence intensity showed no intergroup differences across brain regions. However, post‐perfusion analyses revealed significantly lower EB fluorescence in the parenchyma of Sh‐ECE‐1 mice versus Controls in the cortex (Figure 9H,I), hippocampus (Figure S14G,H, Supporting Information), and striatum (Figure S14I,J, Supporting Information). Furthermore, ECE‐1 knockdown reduced the frequency of spontaneous seizures 4 weeks post‐SE (Figure 9J). TEM results showed that the HRP‐filled caveolae vesicles within brain endothelial cells of Sh‐ECE‐1‐treated SE‐24 h mice was markedly decreased compared to Sh‐NC‐treated Controls (Figure 9K,L), suggesting decreased transcytotic activity following ECE‐1 knockdown. Consistent with these findings, immunoblot analysis confirmed significant downregulation of caveolin‐1 expression in endothelial cells isolated from Sh‐ECE‐1‐treated SE‐24 h mice (Figure 9M,N).

Taken together, these results suggest that ECE‐1 knockdown ameliorates BBB damage and reduces spontaneous seizure frequency following SE.

### 2.9 ET‐1 Down Regulates the Expression of Tight Junction Proteins by Activating the p38/MAPK Pathway

**Figure 10:**
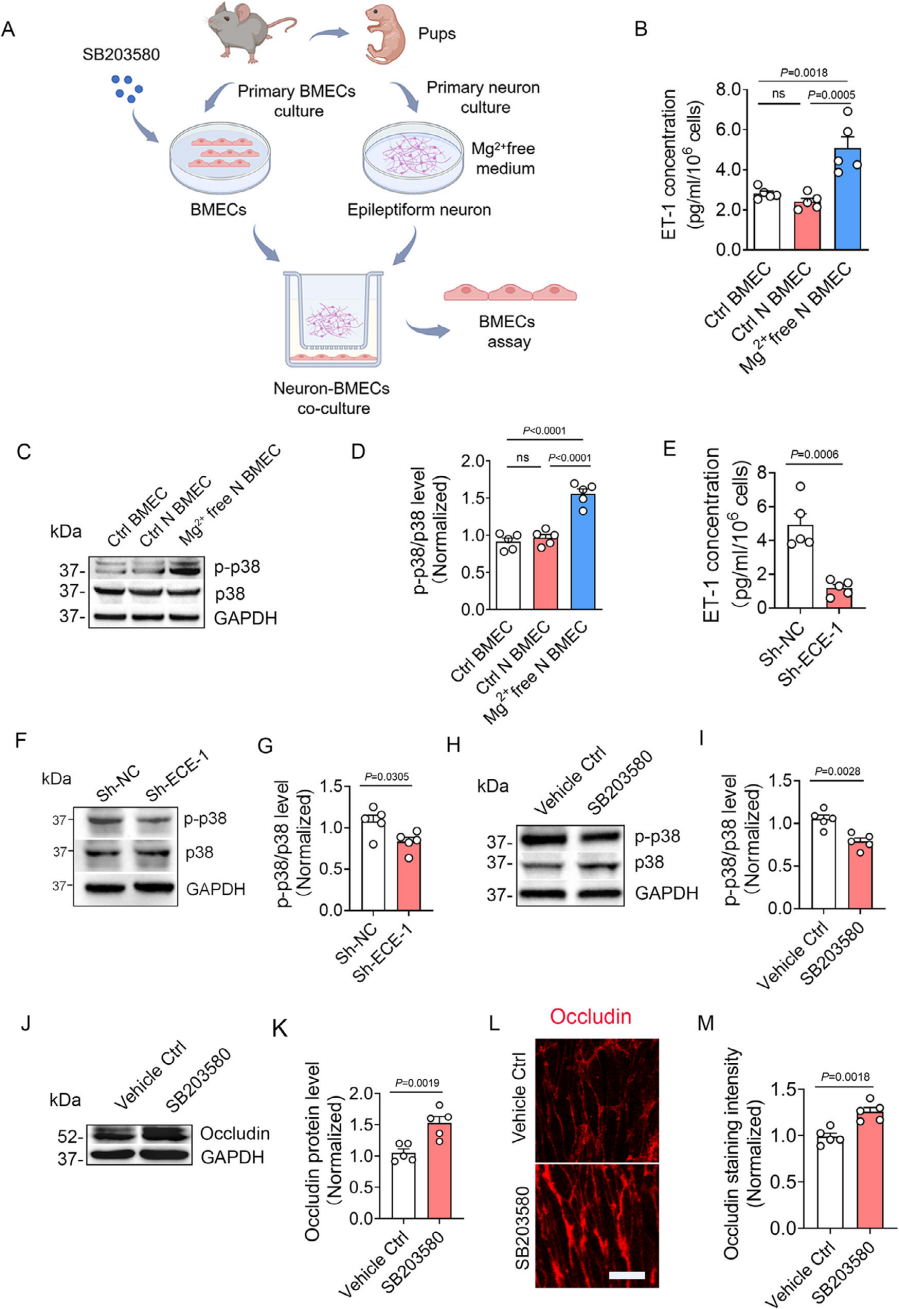
ET‐1 downregulates the expression of tight junction protein via p38/MAPK signaling pathway activation. A) Schematic diagram illustrating the BMECs‐epileptic neuron co‐culture system. Prior to co‐culture with free‐Mg^2+^‐treated epileptic neurons, BMECs were pretreated with the p38/MAPK inhibitor SB203580 for 1 h. SB203580 was maintained throughout the co‐culture period to ensure continuous pathway inhibition. B) Elisa analyses of ET‐1 levels in Ctrl BMEC, Ctrl N BMEC, and Mg^2^⁺ free N BMEC (*n =* 5; ns, Ctrl N BMEC versus Ctrl BMEC; *p =* 0.0005, Mg^2^⁺ free N BMEC versus Ctrl N BMEC; *p =* 0.0018, Mg^2^⁺ free N BMEC versus Ctrl BMEC). C) Western blot analyses showing p‐p38/p38 levels in Ctrl BMEC, Ctrl N BMEC, and Mg^2^⁺ free N BMEC. D) Bar graph quantifying p‐p38/p38 levels (*n =* 5; ns, Ctrl N BMEC versus Ctrl BMEC; *p <* 0.0001, Mg^2^⁺ free N BMEC versus Ctrl N BMEC; *p <* 0.0001, Mg^2^⁺ free N BMEC versus Ctrl BMEC). E) Elisa analyses of ET‐1 levels in Sh‐NC or Sh‐ECE‐1 treated Mg^2^⁺ free N BMECs (*n =* 5, *p =* 0.0006, Sh‐ECE‐1 versus Sh‐NC). F) Western blot analyses showing p‐p38/p38 levels in Sh‐NC or Sh‐ECE‐1 treated Mg^2^⁺ free N BMEC. G) Bar graph quantifying p‐p38/p38 levels (*n =* 5, *p =* 0.0305, Sh‐ECE‐1 versus Sh‐NC). H) Western blot analyses showing p‐p38/p38 levels in vehicle Control (PBS) and SB203580 treated Mg^2^⁺ free N BMECs. I) Bar graph quantifying p‐p38/p38 levels (*n =* 5, *p =* 0.0028, vehicle Control versus SB203580 treated Mg^2^⁺ free N BMECs). J) Western blot analyses showing Occludin protein levels in vehicle Control and SB203580 treated Mg^2^⁺ free N BMECs. K) Bar graph quantifying Occludin protein levels (*n =* 5, *p =* 0.0019, vehicle Control versus SB203580 treated Mg^2^⁺free N BMECs). (L) Representative images of Occludin immunostaining in vehicle Control and SB203580 treated Mg^2^⁺ free N BMECs. M) Bar graph showing quantification of mean fluorescence intensity of Occludin staining (*n =* 5, *p =* 0.0018, vehicle Control versus SB203580 treated Mg^2^⁺ free N BMECs). Data are presented as means ± S.E.M. Statistical analyses was performed using one‐way ANOVA followed by Tukey's test (B, D), unpaired two‐tailed Student's *t*‐test (E, G, I, K, M). Scale bar = 25 µm.

Following confirmation of ECE‐1′s detrimental effects on tight junctions, we attempt to delineate how ECE‐1 exactly regulates the expression of tight junction proteins in brain endothelial cells. ECE‐1 is the major enzyme involved in the synthesis of ET‐1^[^ ^38^ ^]^ ‐a peptide implicated in endothelial integrity disruption.^[^ ^39^, ^40^ ^]^ As expected, Elisa revealed increased ET‐1 level in medium from epileptic neuron‐*co*‐cultured‐BMECs (**Figure** **10**B), while ET‐2 and ET‐3 (also endothelial expressed^[^ ^41^, ^42^, ^43^ ^]^) remained unchanged (Figure S15A,B, Supporting Information). These results implicate ET‐1 as the mediator of ECE‐1‐induced tight junction disruption. Since ET‐1 modulates three major MAPK pathways, including extracellular signal‐related kinase 1/2 (ERK1/2), c‐Jun N‐terminal kinase (JNK), and p38 proteins,^[^ ^44^ ^]^ which directly regulate tight junctions in pathological contexts.^[^ ^45^ ^]^ We first assessed MAPK pathway activation in BMECs under epileptic conditions. Western Blotting detected no significant changes in JNK (Figure S15C,D, Supporting Information) or ERK1/2 phosphorylation (Figure S15E,F, Supporting Information). Notably, p38 phosphorylation was markedly increased (Figure 10C,D), indicating selective p38/MAPK pathway activation. To determine if ET‐1 drives p38 phosphorylation, ECE‐1 was knocked down in epileptic‐condition BMECs. Our data showed that ECE‐1 silencing significantly reduced ET‐1 level (Figure 10E) and suppressed p38 phosphorylation (Figure 10F,G), confirming ET‐1‐dependent p38 activation. Finally, to establish p38's role in tight junction suppression, BMECs were pretreated for 1 h with the p38 inhibitor adezmapimod (SB203580,10 µM) prior to and during 3 h ’ co‐culture with Mg^2+^‐free‐treated neurons (Figure 10A). Pharmacological inhibition attenuated p38 phosphorylation (Figure 10H,I) and rescued expression of tight junction proteins (Figure 10J–M). These results demonstrated that ET‐1 downregulates the expression of tight junction proteins via p38/MAPK pathway activation in brain endothelial cells.

### 2.10 Plasma CircPTPN4 Level is Correlated with BBB Injury at Early Stage of Epileptogenesis

**Figure 11:**
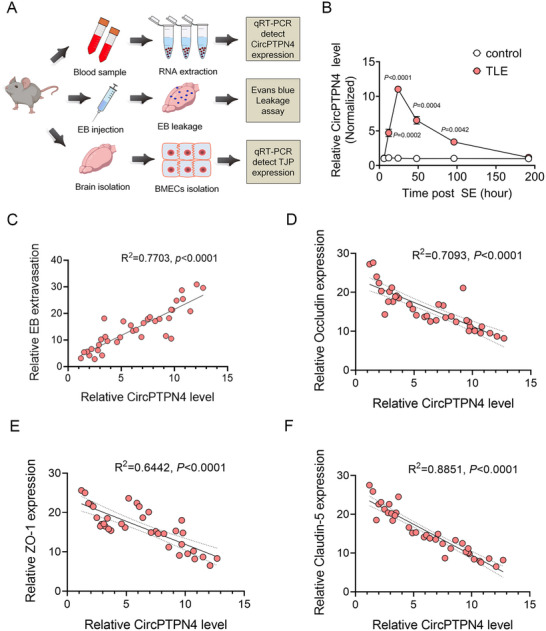
Correlational analyses of plasma CircPTPN4 and BBB injury in epileptic mice. A) Schematic representation of the workflow for correlating plasma CircPTPN4 levels with BBB injury in epileptic mice. B) Line graph depicting plasma CircPTPN4 levels in Control and epileptic mice at 6, 12, 24, 48, 96, and 192‐h post‐status epilepticus (SE) (*n =* 5; *p =* 0.0002 at 12 h; *p <* 0.0001 at 24 h; *p =* 0.0004 at 48 h; and *p =* 0.0042 at 96 h after SE). C) Correlation analysis between plasma CircPTPN4 levels and Evans blue (EB) extravasation in the brain parenchyma (*n =* 36, R^2^ = 0.7703, *p <* 0.0001). D–F) Correlation analysis of plasma CircPTPN4 levels and mRNA expression of tight junction proteins: Occludin (*n =* 36, R^2^ = 0.7093, *p <* 0.0001), ZO‐1 (*n =* 36, R^2^ = 0.6442, *p <* 0.0001), and claudin‐5 (*n =* 36, R^2^ = 0.8851, *p <* 0.0001) in TLE mice. Values are expressed as means ± S.E.M. Statistical significance was assessed using an unpaired two‐tailed Student's *t*‐test (B: 6, 12, and 192 h) and unpaired two‐tailed Welch's *t* test (B: 24, 48, and 96 h). Pearson's correlation coefficient was used to estimate correlations (C, D, E, F).

To investigate whether plasma CircPTPN4 correlates with BBB disruption during early epileptogenesis, we analyzed plasma CircPTPN4 expression, Evans blue extravasation, and tight junction protein expression in TLE mice (**Figure** **11**A). Our results showed that plasma CircPTPN4 level exhibited similar temporal dynamics to cortical expression (Figure 5K), peaking at 24 h post‐SE before gradual decline (Figure 11B). Critically, endothelial‐specific CircPTPN4 knockdown (Figure S16A,B, Supporting Information) significantly reduced plasma CircPTPN4 levels versus Controls at all post‐SE time points (Figure S16C–F, Supporting Information), confirming the endothelial origin of plasma CircPTPN4 in epileptic mice.

Furthermore, correlation analyses revealed a strong positive correlation between plasma CircPTPN4 level and parenchymal EB extravasation (Figure 11C) and a significant inverse correlation between plasma CircPTPN4 level and tight junction protein expression (Figure 11D–F). Taken together, these results suggest that plasma CircPTPN4 levels reflect BBB disruption severity during early epileptogenesis and represent a potential biomarker for BBB damage.

## 3 Discussion

CircRNAs are a newly discovered class of endogenous non‐coding RNAs that exhibit complex spatiotemporal expression patterns. Recently, a large number of highly expressed circRNAs have been identified in the human and mouse brain. The function of circRNAs is closely linked to their cellular localization. CircRNAs in the nucleus are involved in transcription, alternative splicing, and the regulation of chromatin loops.^[^ ^46^ ^]^ Upon translocation to the cytoplasm, circRNAs act as competing endogenous RNAs (ceRNAs), functioning as miRNA sponges. These molecules bind to miRNAs, thereby preventing their interaction with and repression of target messenger RNAs (mRNAs).^[^ ^47^ ^]^ Our study establishes that cytoplasmic CircPTPN4 acts as a ceRNA, sequestering miR‐145a‐5p to upregulate endothelin‐converting enzyme‐1 (ECE‐1). This elevates endothelin‐1 (ET‐1) production, which activates the p38/MAPK pathway to downregulate tight junction proteins (Occludin, claudin‐5, ZO‐1). This molecular cascade mediates blood‐brain barrier (BBB) disruption during early epileptogenesis and increases spontaneous recurrent seizure frequency in chronic epilepsy (Figure S17, Supporting Information).

The BBB regulates the transit of ions, molecules, and cells between the circulation and brain parenchyma.^[^ ^48^ ^]^ Murine studies indicate BBB leakage peaks 1‐2 days following epileptogenic insults, subsequently declining over time.^[^ ^49^, ^50^ ^]^ Consistent with prior findings, our data demonstrate maximal BBB damage in mice occurs 24 h post‐SE, a pattern observed uniformly across brain regions in TLE mice. Composed primarily of brain microvascular endothelial cells (BMECs) and tight junction proteins (TJPs; e.g., Occludin, claudin‐5, ZO‐1), the BBB exhibits detectable leakage in rodent SE models within the initial days, progressively resolving over subsequent weeks and correlating with spontaneous seizures onset.^[^ ^51^ ^]^ Similarly, our results show significant TJP disruption and increased BBB permeability at 24 h post‐SE, followed by recovery by 7 days. Although BBB damage is most acute during early epileptogenesis, its effects persist and may critically influence chronic epilepsy development.^[^ ^52^ ^]^ BBB disruption potentially contributes to epileptogenesis post‐SE via direct mechanisms (e.g., neuronal depolarization from potassium influx) and indirect mechanisms (e.g., glial activation, neuroinflammation, synaptogenesis),^[^ ^53^, ^54^ ^]^ which may mediate SRS chronically. Astrocytes, which regulate seizure‐associated neurotransmitters (e.g., glutamate, GABA), ions, water, pH, and glial transmitters release, thereby modulating synaptic transmission and neuronal excitability^[^ ^55^ ^]^ ‐become dysfunctional following BBB compromise,^[^ ^56^ ^]^ contributing to abnormal neuronal excitability. Increased BBB permeability during early epileptogenesis is linked to significant neuroinflammation and targeted neuronal degeneration,^[^ ^57^ ^]^ partly attribute to monocyte infiltration into the parenchyma. Infiltrating monocytes release cytokines, activate microglia, and generate pro‐inflammatory mediators,^[^ ^58^ ^]^ potentially leading to neuronal damage, hyperexcitability, and spontaneous seizures. Epileptic seizures trigger aberrant neuronal proliferation, including mossy fibers sprouting^[^ ^59^ ^]^ and the ectopic granule cell emergence in the hippocampal dentate gyrus.^[^ ^60^ ^]^ These cellular changes are believed to be closely associated with the progression of epilepsy. While nitric oxide (NO) is crucial for synaptic transmission and synaptic plasticity,^[^ ^61^ ^]^ elevated neuronal nitric oxide synthase (nNOS) expression and enhanced enzymatic activity post‐SE may contribute to abnormal neuronal proliferation^[^ ^62^ ^]^ and altered neuronal plasticity. Consequently, BBB disruption represents a significant long‐term factor in spontaneous seizure susceptibility. In vitro evidence indicates neurons during epileptic activity release excessive glutamate, damaging BMEC tight junctions and increasing BBB permeability, suggesting seizure‐driven glutamate directly compromises TJ integrity. Furthermore, seizure‐associated glutamate release upregulates matrix metalloproteinases (MMP‐2 and MMP‐9), which degrades the perivascular extracellular matrix and TJP components, thereby promoting BBB damage.^[^ ^63^, ^64^ ^]^

CircRNAs in endothelial cells have been shown to regulate the integrity of the BBB. For example, CircFoxO3 upregulation in endothelial cells attenuates BBB impairment via autophagy activation,^[^ ^65^ ^]^ while Circ_2858 promotes BBB disruption by sponging miR‐93‐5p to elevate VEGFA expression during Escherichia coli meningitis.^[^ ^66^ ^]^ In this study, we identify CircPTPN4 as a critical regulator of BBB integrity in a murine TLE model. FISH assay revealed epilepsy‐specific upregulation of CircPTPN4 in BMECs but not neurons. CircPTPN4 knockdown in endothelial cells attenuated early BBB impairment and reduced chronic‐phase spontaneous seizure frequency, indicating its dual role in mediating initial BBB damage and sustaining persistent proepileptogenic effects. To evaluate whether CircPTPN4 upregulation is epilepsy‐dependent, we assessed its expression in Control and oxygen‐glucose deprivation (OGD)‐treated BMECs. Notably, CircPTPN4 levels remained unchanged following OGD despite significant tight junction disruption, demonstrating that OGD‐induced tight junction disruption occurs independently of CircPTPN4 and confirming its specificity to epileptic pathogenesis.

MicroRNAs (miRNAs) serve as key post‐transcriptional regulators of gene expression by binding target sites within the untranslated regions (UTRs) of messenger RNAs. CircRNAs can function as competing endogenous RNAs (ceRNAs), sequestering miRNAs to promote target gene expression competitively. Bioinformatics analyses identified a binding site between CircPTPN4 and miR‐145a‐5p, which was experimentally validated via RNA pull‐down and FISH assays. Although miR‐145a‐5p is established as a tumor suppressor in oncology,^[^ ^67^ ^]^ emerging evidence implicates it in endothelial cell function and vascular regulation.^[^ ^68^ ^]^ Here we observed cytoplasmic co‐localization of CircPTPN4 and miR‐145a‐5p in BMEC. Under epileptic condition, miR‐145a‐5p expression was downregulated, exhibiting an inverse correlation with CircPTPN4 upregulation. These results indicate that CircPTPN4 mediates endothelial dysfunction and BBB impairment through a ceRNA mechanism by sponging miR‐145a‐5p.

Recent studies indicate that miR‐145a‐5p is expressed in neurons and modulates neural proliferation and synaptic plasticity.^[^ ^37^, ^69^ ^]^ Consistent with these findings, we observed neuronal localization of miR‐145a‐5p via co‐localization with the mature neuron marker NeuN. However, unlike in BMECs, miR‐145a‐5p expression in neurons remained stable under epileptic conditions. This cell‐type‐specific regulation suggests miR‐145a‐5p mediates cell‐autonomous effects within BMECs to modulate BBB integrity in epilepsy.

To identify miR‐145a‐5p target genes, we conducted bioinformatics analyses and pinpointed the ECE‐1 as a key candidate. Consistent with CircPTPN4 dynamics, ECE‐1 expression was upregulated at 24 h post‐SE and normalized by 7 days in the cortex of TLE mice. Furthermore, modulation experiments confirmed that miR‐145a‐5p mimic transfection downregulated ECE‐1, while miR‐145a‐5p inhibitors upregulated ECE‐1 in both Control and epileptic BMECs. Critically, CircPTPN4 knockdown via shRNA lentivirus attenuated anti‐miR‐145a‐5p‐induced ECE‐1 upregulation. Conversely, miR‐145a‐5p mimic transfection rescued ECE‐1 overexpression induced by CircPTPN4 ectopic expression. These data establish miR‐145a‐5p the function mediator through which CircPTPN4 regulates ECE‐1.

In this study, ECE‐1 knockdown improved BBB integrity in epileptic mice, indicating its critical role in BBB impairment. As the primary enzyme for endothelin‐1 (ET‐1) synthesis, ECE‐1 elevation drives BBB dysfunction through ET‐1 overproduction. Previous studies demonstrate ET‐1 induces tight junction disruption^[^ ^39^, ^40^ ^]^ and activates the mitogen‐activated protein kinase (MAPK) pathway,^[^ ^70^, ^71^, ^72^ ^]^ which directly regulate tight junction proteins expression.^[^ ^73^, ^74^ ^]^ We therefore propose that ET‐1 mediates tight junction downregulation via MAPK pathway activation in epileptic condition. Validation experiments revealed concomitant elevation of ET‐1 and phospho‐p38/MAPK in BMECs co‐cultured with epileptic neurons. ECE‐1 knockdown reduced both ET‐1 production and p38/MAPK phosphorylation. Critically, p38/MAPK inhibition significantly upregulated tight junction protein expression in epileptic neuron co‐cultured BMECs. These findings establish that ET‐1 increases BBB permeability by activating the p38/MAPK pathway to suppress tight junction protein expression.

Given the established role of BBB injury in epileptogenesis, early detection and restoration of BBB integrity represent critical therapeutic targets. Liquid biopsy has emerged as a significant non‐invasive diagnostic modality,^[^ ^75^, ^76^ ^]^ with peripheral blood serving as a primary biofluid source. Circular RNAs are endogenously synthesized and enter circulation via exosome transport and passive leakage during pathology.^[^ ^77^ ^]^ Their covalently closed circular structure confers exceptional RNase resistance, ensuring remarkable plasma stability.^[^ ^24^ ^]^ Plasma circRNAs currently serve as diagnostic biomarkers in oncology (e.g., circFARSA in lung cancer,^[^ ^78^ ^]^ hsa_circ_0000467 in gastric cancer^[^ ^79^ ^]^). In this study, plasma CircPTPN4 levels mirrored expression patterns in BMECs of epileptic mice, indicating its reflection of cerebral endothelial dynamics. Importantly, plasma CircPTPN4 positively correlated with early‐stage BBB injury severity. These findings position CircPTPN4 quantification as a promising non‐invasive approach for assessing BBB integrity in epilepsy patients.

In conclusion, our findings establish that CircPTPN4 compromises BBB integrity during early epileptogenesis by competitively binding miR‐145a‐5p to upregulate ECE‐1. Subsequent ECE‐1‐mediated ET‐1 synthesis activates the p38/MAPK pathway, downregulating tight junction protein expression and increasing vascular permeability. Crucially, CircPTPN4 knockdown attenuated tight junction disruption, reduced acute BBB damage, and decreased chronic spontaneous seizure frequency. These findings suggest that targeting the CircPTPN4/miR‐145a‐5p/ECE‐1 signaling pathway could be a promising strategy for developing novel therapeutics aimed at preserving BBB function and mitigating seizure activity in epilepsy.

## 4 Experimental Section

### Animals

Male C57BL/6 J mice (4–6 weeks old, weighing 19 ± 2 g) were obtained from the Nanjing Biomedical Research Institute of Nanjing University (NBRI, Nanjing, China). The mice were housed in an air‐conditioned room at 22 ± 1 °C with a 12‐h light/dark cycle (lights on from 7:00 A.M. to 7:00 P.M.). Food and tap water were provided ad libitum. All procedures were approved by the Animal Care and Use Committee of the Medical School of Southeast University (protocol number: 017 042 0003) and were conducted in accordance with the National Institutes of Health Guide for the Care and Use of Laboratory Animals. Every effort was made to minimize animal suffering and discomfort and to reduce the number of animals used. In experiments, mice of the same age, size, sex, and genetic background were randomly arranged into Control and treatment groups.

### Pilocarpine Induction of TLE

The temporal lobe epilepsy (TLE) model was induced as previously described,^[^ ^80^ ^]^ with some modifications. Mice were intraperitoneally (i.p.) administered scopolamine methyl bromide (Sigma, Cat. No. S8502, 2 mg kg^−1^) dissolved in saline 30 min prior to pilocarpine injection to block the peripheral effects of pilocarpine. Pilocarpine hydrochloride (TRC, Cat. No. P441500, 290 mg kg^−1^, i.p.) was then administered to induce status epilepticus (SE). Control mice were intraperitoneally (i.p.) administered twice of saline, with the same dosage and time interval with scopolamine methyl bromide and pilocarpine. The pilocarpine dosing regimen was determined based on the previous studies^[^ ^81^, ^82^, ^83^ ^]^ and the preliminary experiments to determine the dosage. These results showed that when mice were treated with pilocarpine at the dosage of 290 mg kg^−1^, with the mice exhibited typical behavior seizures (>4 based on Racine Scale), which is characterized by status epilepticus. Furthermore, the mortality rate of the mice treated at this dosage was as low as 20%. Seizure intensity was assessed using the Racine scale: Stage 1, mouth and facial movements; Stage 2, head nodding; Stage 3, forelimb clonus; Stage 4, seizures characterized by rearing; Stage 5, seizures characterized by rearing and falling. Animals exhibiting behavioral seizures with a Racine score > 4 more than once, along with EEG recording, were classified as status epilepticus. To terminate status epilepticus, mice received an intraperitoneal injection of diazepam (10 mg kg^−1^), while Control mice were administered an equivalent volume of saline.

### Magnetic Resonance Imaging Scan and Image Analyses

Magnetic resonance imaging (MRI) was performed using a 7.0‐Tesla horizontal scanner (Bruker, Ettlingen, Germany, PharmaScan) at Southeast University. Mice were initially anesthetized with isoflurane (2%–3% in a 70/30 N_2_/O_2_ mixture) and head‐fixed in an animal carrier using tooth and ear bars. Fixation and anesthesia were necessary to minimize movement artifacts. The breathing rate of the mice was maintained at 100–120 breaths per minute by adjusting the isoflurane concentration during image acquisition. T2‐weighted scans were performed at 24 h and 7 days after status epilepticus (SE), with Control mice used for comparison. T2‐weighted imaging (T2WI) parameters were as follows: TR = 30 000 ms, TE = 10.0 ms, FOV = 31 × 20 mm, matrix = 256 × 256, flip angle = 128°, slice thickness = 1 mm, slice spacing = 1 mm, and number of excitations (NEX) = 4. The acquired MRI data were exported in DICOM format using Paravision 6.0.1, and the DICOM images were converted to Neuroimaging Informatics Technology Initiative (NIfTI) format using MRIcron (dcm2nii tool). The strong bias field produced by the instrument's surface coil was corrected using AIDAmri. The edema area in mice after SE was identified based on the higher signal intensity in T2‐weighted images compared to Control mice.

### Quantitative Real‐Time PCR

Total mRNA and circRNA were extracted from brain microvascular endothelial cells (BMECs) or brain tissues using TRIzol reagent (Vazyme Biotech, Cat. No. R401‐01). Total RNA from plasma was isolated using the MolPure Blood RNA Kit (YEASEN, Cat. No. 19241ES50) according to the manufacturer's instructions. cDNA was synthesized using the HiScript III RT SuperMix for qPCR (+gDNA wiper) (Vazyme Biotech, Cat. No. R323‐01). RNA and cDNA concentrations were measured using a spectrophotometer (OD‐1000, Wuyi Technology, Nanjing, China). Quantitative real‐time PCR assays were performed using the AceQ qPCR SYBR Green Master Mix (Low ROX Premixed) (Vazyme Biotech, Cat. No. Q131‐02) on a quantitative real‐time PCR system (Applied Biosystems, Foster City, CA, USA). The PCR conditions included one cycle at 95 °C for 5 min, followed by 40 cycles at 95 °C for 10 s and 60 °C for 30 s. Relative mRNA expression was normalized to GAPDH mRNA levels in the same samples and calculated using the ΔΔCt method. For circRNA amplification, divergent and convergent primer sets were used. Total miRNA was isolated using the MiPure Cell/Tissue miRNA Kit (Vazyme Biotech, Cat. No. RC201) and reverse transcribed into cDNA using the miRNA first Strand cDNA Synthesis Kit (by stem‐loop) (Vazyme Biotech, Cat. No. MR101) according to the manufacturer's instructions. The reagents and conditions for miRNA real‐time PCR were identical to those used for circRNA analyses. miR‐145a‐5p qRT‐PCR primers were designed using Vazyme miRNA Design V1.01 software. miRNA levels were normalized to the expression of the small RNA U6. All primers used in this study are listed in Table S1, Supporting Information.

### Western Blot Assay

Brain tissues were homogenized in RIPA lysis buffer (Beyotime, Cat. No. P0013B) supplemented with a protease inhibitor cocktail (EDTA‐free, 100× in DMSO) (Med Chem Express, Cat. No. HY‐K0010) on ice. After centrifugation at 12 000 rpm for 25 min, the protein content in each supernatant was determined using Pierce BCA Protein Assay Kit (Pierce, Cat. No. 23 227) and quantified to the same concentration. Samples were boiled at 100 °C for 5 min in sodium dodecyl sulfate (SDS) loading buffer to denature the proteins. Proteins with varying molecular weights were separated using 12% SDS‐polyacrylamide gels (SDS‐PAGE) and transferred to nitrocellulose membranes (Merck Millipore, Cat. No. HATF00010) using a Bio‐Rad Mini Protean III wet transfer unit (Bio‐Rad, Hercules, CA, USA). Membranes were incubated with 5% nonfat milk in TBST (0.01% Tween‐20) for 1 h, followed by incubation with specific primary antibodies at 4 °C overnight. After three washes with 1× TBST to remove unbound antibodies, membranes were re‐incubated for 2 h with the appropriate HRP‐conjugated secondary antibodies. Signals were detected digitally using Chemistar High‐Sig ECL Western Blotting Substrate (Tanon, Cat. No. 180‐5001). The blot intensity was quantified using ImageJ software (NIH, version 1.8.0). The primary antibodies used for Western blotting are listed in Table S2, Supporting Information.

### Immunofluorescence

Brain tissues or cells were fixed in ice‐cold 4% paraformaldehyde solution overnight or for 30 min, respectively. After dehydration in 30% sucrose solution, brain tissues were cut into 35 µm‐thick slices. Fixed brain tissue slices or cells were permeabilized with 0.1% Triton X‐100 in PBS for 15 min, blocked with 5% BSA at room temperature for 1 h, and then incubated overnight at 4 °C with specific antibodies. The tissues or cells were washed three times with PBS for 15 min each and incubated with appropriate secondary antibodies (1:200, Yeasen Biotechnology) for 2 h at room temperature. The slides were rinsed and mounted. Images were captured using a laser confocal microscope (Nikon A1R HD25, Tokyo, Japan). Image processing and fluorescence quantification were performed using ImageJ software (NIH, version 1.8.0) by an investigator blinded to group assignments. The primary antibodies used for immunofluorescence are listed in Table S2, Supporting Information.

### BBB Integrity Evaluation with Tracer

For detection of EB leakage, mice were intravenously injected with 6 µL g^−1^ of body weight of 2% (wt/vol) Evans Blue (EB) (Sigma, Cat. No. E2129) and circulated for 6 h. For detection of dextran leakage, 10 mg kg^−1^ of 40 kDa Dextran (Sigma, Cat. No. FD40S) dissolved in saline was administered in mice and circulated for 10 min. In order to observe more clearly the accumulation of tracers in brain parenchyma, mice deeply anesthetized with isoflurane were underwent cardiac perfusion to remove dyes from the blood vessels. A total of 200 mL of saline was administered to ensure thorough removal of blood and tracers in vascular, till the limbs and liver looks paleness. The perfusion pressure was maintained at 6.0 mmHg, which was determined to be the optimal rate for achieving continuous outflow of the perfusate. Following this, 200 mL of 4% paraformaldehyde was perfused for tissue fixation. For detection of Sulfo‐NHS‐LC‐Biotin leakage, deeply anesthetized mice were perfused with 35 mL 5 mg mL^−1^ Sulfo‐NHS‐LC‐Biotin (Med Chem Express, Cat. No. HY‐D0799A) dissolved in PBS. The perfusion pressure was maintained at 2.0 mmHg. The brain tissue was then fixed with 4% paraformaldehyde overnight and dehydrated in a 30% sucrose solution. EB staining on the brain surface was captured, and the brain tissue was then cut into thick slices for visual observation of EB leakage in different brain regions. Brains were also cut into 35 µm slices for microscopic observation of EB autofluorescence (excitation wavelength: 633 nm; emission wavelength: 640–700 nm) or for further immunostaining. For Evans Blue quantification in the brain parenchyma, equal‐weight hemispheres of perfused mice were homogenized in 50% trichloroacetic acid (Aladdin, Cat. No. T104257). After centrifugation at 1000 g for 10 min, the pH of the supernatant was adjusted to neutral with 5 M NaOH. The absorbance of EB was measured at 620 nm using a microplate reader (Biotech, USA). Mice brain tissues were also dissected immediately following cervical dislocation, washed with PBS, and fixed with 4% paraformaldehyde supplemented with 0.025% glutaraldehyde (Adamas‐beta, Cat. No. 0 100 8335) to preserve intravascular EB or dextran. Additionally, 35 µm thick brain tissue slices were acquired for further observation. Images were captured using a laser confocal microscope (Nikon A1R HD25, Tokyo, Japan) with consistent settings, primarily including the following elements: The “Ch Series” feature was enabled during the imaging process to prevent color crossover. The three main parameters influencing signal strength are the detector's sensitivity (“HV”) set to 42, the “Offset” configured to 0, and the laser power set at 0.5. No changes were made to display and adjustment settings during imaging. Pictures of ROI positions in cortical regions were determined with similar vascular density. Furthermore, image acquisition regions across different experimental groups were kept as consistent as possible. A series of ROI mean values were normalized to the average of the Control group. Specifically, the mean fluorescence intensity value (µControl) for the Control group data is calculated first, and then all values (including those from the Control group) are compared to µControl. Image processing and fluorescence quantification were performed using ImageJ software (NIH, version 1.8.0) by an investigator blinded to group assignments.

### Transmission Electron Microscopy

TEM imaging was done as previous described with some modifications.^[^ ^9^ ^]^ Mice were intravenously injected with 0.5 mg g^−1^ of body weight of horseradish peroxidase (Sigma Aldrich, HRP, type II, Cat. No. P8250) dissolved in PBS. After 30 min of HRP circulation, 1 mm^3^ brain tissues were dissected and fixed by immersion in 0.01 M Phosphate Buffer Solution (2.5% glutaraldehyde, Pinuofei, PN0019) for 1 h at room temperature (RT) followed by fixation with 1% osmium acid in 0.1 M phosphate buffer solution (with pH 7.4) at room temperature in the dark for 2 h. Fixed tissues were washed overnight in 0.1 M sodium cacodylate buffer and then cut in 50 µm‐thick free floating sections using a vibrotome. Sections were incubated for 45 min at RT in 0.05 M Tris‐HCl pH 7.6 buffer, containing 5 mg/10 mL of 3‐3′ diaminobenzidine (DAB, Med Chem Express, Cat. No. HY‐W014212) with 0.01% hydrogen peroxide. The samples were prepared by Pinuofei Biological Co., Ltd (Wuhan, China). The steps are as follows: After rinsed with 0.1 M phosphate‐buffered saline (PBS, pH 7.4), tissues were then dehydrated with different concentration gradients of ethanol and 100% acetone. After embedding with acetone and 812 embedding agent, tissues were polymerized for 48 h and then ultra‐thin sections (80 nm) were prepared and the sections were collected using a 150‐mesh square Huaguan membrane copper mesh. The copper mesh was stained with a 2% acetic uranium saturated alcohol solution in the dark for 8 min, after washing, the copper mesh was then stained with a 2.6% citrate lead solution under carbon dioxide‐free conditions for 8 min. This work also prepared samples without HRP injection. The images were obtained using the transmission electron microscope (H‐7650C) of Southeast University. The magnification of the image is 4000X.

### Primary Brain Microvascular Endothelial Cells Culture

The neocortex was dissected from 4–6‐week‐old C57BL/6J mice and carefully remove large blood vessels by rolling it on filter paper. The brain tissue was then cut into 1 mm^3^ pieces using scissors in DMEM/F12 medium (Gibco, Cat. No. C11330500BT) and digested with a solution containing collagenase I (Gibco, Cat. No. 9001‐12‐1), 0.8% Trypsin‐EDTA (Beyotime, Cat. No. C0201), and 1% DNase I (Sigma, Cat. No. 10 104 159 001) for 1.5 h at 37 °C with gentle rotation. After digestion, the mixture was centrifuged at 1000 rpm for 5 min to remove the digestion fluid. The pellet was resuspended in 20% bovine serum albumin (BSA, Yuanye Bio‐Technology, Cat. No. BIO‐000001) and centrifuged at 1000 g for 20 min at 4 °C. Brain microvascular endothelial cells were collected from the bottom of the tube and resuspended in DMEM/F12 supplemented with 15% FBS (Gibco, Cat. No. A5669701), murine EGF (Pep Rotech, Cat. No. 315‐09, 0.005%), murine bFGF (Pep Rotech, Cat. No. 450‐33, 0.1%), vitamin C (Solar Bio, Cat. No. A8100, 0.01%), hydrocortisone (Solar Bio, Cat. No. G8450, 0.2%), heparin sodium (Solar Bio, Cat. No. H8060, 0.068%), and L‐glutamine (Beyotime, Cat. No. C0905, 1%). The cells were then plated on 0.1% gelatin (Solar Bio, Cat. No. G0040)‐coated 6‐well plates and cultured in a 37 °C, 5% CO_2_ incubator for ≈8 days for further experiments.

### Primary Neuron Culture and Establishment of Epileptiform Discharge Model

The hippocampus was dissected from postnatal day 0 (P0) newborn mice and cut into 1 mm^3^ pieces in pre‐cooled DMEM/F12 medium (Gibco, Cat. No. C11330500BT). The tissue was then digested with 0.125% trypsin (Gibco, Cat. No. 25 200 056) in a 37 °C incubator for 10 min. After digestion, the reaction was terminated by adding fetal bovine serum (FBS, Gibco, Cat. No. A5669701), and the mixture was centrifuged at 1000 rpm for 5 min. The pellet was resuspended in neurobasal medium (Gibco, Cat. No. 21 103 049) supplemented with B27 (Gibco, Cat. No. 17 504 044) and gently triturated using a pipette. The resulting suspension was filtered through a 40 µm nylon mesh and plated onto Poly‐D‐Lysine (PDL, Beyotime, Cat. No. C0312)‐coated 6‐well plates. The cells were then cultured in a 37 °C, 5% CO_2_ incubator for ≈7 days for subsequent experiments. To establish an in vitro neuronal epilepsy model, hippocampal neurons were cultured in a magnesium‐free extracellular solution (145 mM NaCl, 2.5 mM KCl, 10 mM HEPES, 2 mM CaCl_2_, 10 mM glucose, 0.002 mM glycine in distilled water, pH 7.2, adjusted to 325 mOsm with sucrose) at 37 °C for 3 h. Epileptiform activity was then assessed by whole‐cell current‐clamp recordings.

### Whole Cell Current‐Clamp Recording

Whole cell current‐clamp recording was performed as we described previously.^[^ ^84^ ^]^ Neurons were treated with magnesium‐free medium to induce seizure activity. The electrophysiological recordings were initiated by replacing the culture medium with a recording solution containing: 145 mM NaCl, 10 mM HEPES, 10 mM glucose, 2.5 mM KCl, 2 mM CaCl_2_, 0.002 mM glycine, 1 mM MgCl_2_, pH 7.3, and osmolality adjusted to 325 mOsm with sucrose. Neurons were then continuously monitored in whole‐cell current‐clamp mode using a Multiclamp 700B patch clamp amplifier and an Axon Digidata 1550 A/D converter (Molecular Devices). The recording chamber was perfused continuously with the recording medium at a flow rate of 0.5 mL min^−1^. All experiments were conducted at room temperature. To assess neuronal firing patterns, 1000 ms depolarizing current steps, starting at 10 pA and increasing in 30 pA increments, were applied from a holding potential of ‐70 mV. Action potentials were elicited with 5 ms depolarizing current pulses. The number of action potentials was quantified using the threshold search function in Clampfit (Molecular Devices, version 10.6).

### Oxygen Glucose Deprivation and Cell Viability Assay

BMECs cultured on 96‐well plates were allowed to reach 80% confluence. For the oxygen‐glucose deprivation (OGD) assay, BMECs were placed in glucose‐free, deoxygenated DMEM (Gibco, Cat. No. 11 966 025) and exposed to an oxygen‐free environment (95% N_2_, 5% CO_2_) for 5 h. Cells cultured under normal conditions served as Controls. Cell viability was assessed using the CCK‐8 kit (Beyotime, Cat. No. C0037) according to the manufacturer's instructions. Briefly, 20 µL of CCK‐8 solution was added to the BMECs, followed by a 2‐h incubation. The absorbance was measured at 450 nm using a microplate reader (Biotech, USA).

### Sodium Fluorescein Permeability Assay

BMECs were cultured in the upper chamber of a Transwell insert with a pore size of 0.4 µm. The medium in both the upper and lower chambers was maintained at the same level, ensuring contact between the two. A complete monolayer cell barrier was formed to assess permeability. After exposure to virus or co‐culture with epileptiform neurons, the medium in the upper chamber was replaced with the same medium as in the lower chamber, with the addition of 10 µg mL^−1^ Na‐F (Sigma, Cat. No. F6377). After incubating at 37 °C for 60 min, the fluorescence intensity in the lower chamber was measured at 488 nm using a microplate reader (Biotech, USA).

### Circular RNA Library Construction and Sequencing

Total RNA was isolated and purified from the cortex of three Control male mice (6 weeks old) and three epileptic male mice (6 weeks old) using TRIzol reagent (Vazyme Biotech, Cat. No. R401‐01). Ribosomal RNA was removed using the Illumina Ribo‐Zero Gold Kit. RNA purity was assessed with a Kaio spectrophotometer (Kaio, Beijing). RNA integrity and concentration were evaluated using the Agilent 2100 RNA Nano 6000 Assay Kit (Agilent Technologies, Cat. No. 5067‐1511). Long non‐coding RNA (lncRNA) libraries were constructed using 3 µg of total RNA from each sample. Different index tags were selected to build the library, following the instructions provided in the NEB Next Ultra Directional RNA Library Prep Kit for Illumina (New England BioLabs, Cat. No. E7760). The library was sequenced on an Illumina platform (PE150) by Annoroad Gene Technology (Beijing) Co., LTD.

### Circular RNA Data Analyses

The raw data were filtered to generate high‐quality clean reads by removing those with contaminated junctions, low‐quality reads (where the Phred Quality Score ≤ 19 for more than 15% of the total bases), reads with an N ratio greater than 5%, and those that matched ribosomal RNA. Circular RNAs (circRNAs) were identified using CIRI. The sequences were initially split using the BWA‐MEM algorithm, and the results were compared to identify paired chiastic clipping and paired‐end mapping sites, as well as GT‐AG splicing signals. Sequences with junction sites were re‐matched for circRNA identification using a dynamic programming algorithm to ensure reliability. The expression levels of circRNAs were quantified using the Spliced Reads per Billion Mapping normalization method. Differential expression analyses of circRNAs were performed using DESeq2. circRNAs with Log2 fold change ≥ 1.5 or ≤ −1.5 and *p <* 0.05 were considered markedly differentially expressed. The numbers of up‐regulated and down‐regulated circRNAs were then determined. Additionally, Gene Ontology terms and Kyoto Encyclopedia of Genes and Genomes pathways associated with the differentially expressed circRNA genes were identified.

### Biotinylated RNA Pull‐Down Assay

BMECs cultured on 100 mm × 20 mm Style Dishes (Corning, Cat. No. 430 167) to 80% confluence were used for the CircPTPN4 and miR‐145a‐5p pull‐down assays. For CircPTPN4 to miR‐145a‐5p affinity isolation, BMECs were washed with 1× PBS and lysed with 1 mL of lysis buffer (Beyotime, Cat. No. P0658S) per dish on ice for 10 min. For miR‐145a‐5p to CircPTPN4 affinity isolation, BMECs were transfected with biotinylated wild‐type miR‐145a‐5p (Bio‐miR‐145‐WT) or biotinylated mutant miR‐145a‐5p (Bio‐miR‐145‐MUT) at a final concentration of 100 nM for 48 h. After three washes with ice‐cold PBS, the cells were lysed with lysis buffer. The lysates were centrifuged at 12 000 rpm for 15 min. A total of 50 µL of supernatant from each sample was prepared as input. Biotinylated CircPTPN4 probe (100 nM, RiboBio) was dissolved in binding buffer (0.5 M NaCl, 20 mM Tris‐HCl, pH 7.5, 1 mM EDTA), and the probe was incubated with streptavidin magnetic beads (Beyotime, Cat. No. P0658S) with rotation at 4 °C for 2 h to form the probe‐magnetic bead complex. This complex was then incubated with BMECs supernatant at 4 °C for 4 h with rotation to capture CircPTPN4‐binding miRNAs. For miR‐145a‐5p to CircPTPN4 isolation, lysates of BMECs transfected with biotinylated miRNAs were directly incubated with streptavidin magnetic beads at 4 °C for 4 h with rotation to capture miR‐145a‐5p‐binding circRNAs. The beads were washed three times with wash buffer, and the bound RNA complex was eluted with elution buffer for subsequent quantitative RT‐PCR analyses. The biotinylated wild‐type and mutant miR‐145a‐5p were synthesized by Gene Pharma (Shanghai, China). Their sequences are provided in Table S3, Supporting Information. The primers for GAPDH, CircPTPN4, CircPCNX, U6, miR‐145a‐5p, and miR‐28a‐5p are listed in Table S1, Supporting Information.

### Elisa

BMECs cultured on 96‐well plates were allowed to reach 80% confluence. After coculturing with normal neurons or epileptiform neurons for 3 h, the supernatant was collected and centrifuged at 4 °C for 20 min to remove impurities and cell debris. The ET‐1 content in each supernatant was determined using Enzyme‐Linked Immunosorbent Assay Kit Mouse ET‐1 (Endothelin 1) ELISA Kit (Elabscience, Cat. No. E‐EL‐M2730). The ET‐2 content in each supernatant was determined using Mouse Endothelin 2 (EDN2) ELISA Kit (Jianglai, Cat. No. JL20426). The ET‐3 content in each supernatant was determined using Mouse Endothelin 3 (EDN3) ELISA Kit (Wuxi Donglin Technology Development Co., LTD, Cat. No. DL‐EDN3‐Mu). Briefly, 100 µL each dilution of standard, blank and sample was added into the appropriate wells. Cover the plate with the sealer provided in the kit. Incubate for 90 min at 37 °C. Decant the liquid from each well and immediately add 100 µL of Biotinylated Detection Ab working solution to each well. Cover the plate with a new sealer and incubate for 1 h at 37 °C. Wash three times with wash buffer and pat it dry with clean absorbent paper. Add 100 µL of HRP Conjugate working solution to each well. Cover the plate with a new sealer and incubate for 30 min at 37 °C. After washing five times with wash buffer and patting it dry against clean absorbent paper, 90 µL of Substrate Reagent was added to each well. The plate was covered with a new sealer and incubated for about 15 min at 37 °C. 50 µL of Stop Solution was added to each well and the optical density (OD value) of each well was immediately determined with a microplate reader set to 450 nm. Plot a four‐parameter logistic curve on a log‐log axis, with standard concentration on the x‐axis and OD values on the y‐axis.

### Fluorescence In Situ Hybridization in Combination with Immunostaining

The FISH assay was performed using the Fluorescence in Situ Hybridization Kit (Beyotime, Cat. No. R0306M) following the manufacturer's protocol. Briefly, fixed brain slices or cells cultured on coverslips were permeabilized with 0.3% Triton X‐100 and incubated with proteinase K (20 µg mL^−1^) to expose the epitope for improved binding between the CircPTPN4 probe and miR‐145a‐5p. The samples were then incubated in 0.5 M HCl for 5 min to neutralize basic proteins, followed by acetylation with Acetylation Solution for 10 min. Next, the samples were prehybridized with a hybridization solution for 1 h at 50 °C. To analyze CircPTPN4 expression in BMECs or neurons, the samples were incubated overnight in a hybridization solution containing biotin‐labeled CircPTPN4 probes (50 nM, RiboBio). The following day, the samples were treated with Alexa Fluor 647‐streptavidin (1:200, Invitrogen, Cat. No. S3235), CD31 antibody (1:100, Santa Cruz, Cat. No. S18916), or anti‐NeuN antibody (1:200, Abcam, Cat. No. ab177487). For investigating the binding of CircPTPN4 to miR‐145a‐5p, the samples were incubated overnight with biotin‐labeled CircPTPN4 probes (50 nM, RiboBio) or digoxigenin‐labeled miR‐145a‐5p probes (50 nM, RiboBio). The next day, the samples were treated with Alexa Fluor 488‐streptavidin (1:200, Invitrogen) or anti‐digoxigenin‐poly‐POD antibody (1:200, Roche Diagnostics, Cat. No. 11 207 733 910). After three washes with PBS, miR‐145a‐5p signals were amplified using TSA Cyanine (YEASEN, Cat. No. 60406ES) for 10 min at room temperature. For miR‐145a‐5p expression analyses in neurons, the samples were incubated overnight with digoxigenin‐labeled miR‐145a‐5p probes, followed by treatment with anti‐digoxigenin‐poly‐POD antibody (1:200, Roche Diagnostics, Cat. No. 11 207 733 910) or anti‐NeuN antibody (1:200, Abcam, Cat. No. ab177487) overnight. The miR‐145a‐5p signals were then amplified with TSA Fluorescein (YEASEN, Cat. No. 60405ES) for 10 min at room temperature. The slides were rinsed, mounted, and images were acquired using a laser confocal microscope (Nikon A1R HD25, Tokyo, Japan). Image processing and fluorescence quantification were performed with ImageJ software (NIH, version 1.8.0) by an investigator blinded to the group assignments. The probe sequences are provided in Table S3, Supporting Information.

### Luciferase Assay

The pMIR‐REPORT‐ECE‐1‐3′UTR vector or pMIR‐REPORT‐ECE‐1‐Mutant vector (RiboBio, Guangzhou, China) was constructed using the ECE‐1 3′UTR sequences or mutant sequences, with a constitutive Renilla expression plasmid (Promega) included as an internal Control for transfection efficiency. BMECs were transfected with these plasmids and a miR‐145a‐5p mimic (RiboBio, Guangzhou, China) according to the manufacturer's instructions, with a miRNA Control used as the negative Control. Luciferase activity was measured 24 h ’ post‐transfection using the Dual Luciferase Reporter Gene Assay Kit (YEASEN, 11402ES60) following the manufacturer's protocol. The ECE‐1 3′UTR sequence was obtained from the NCBI database.

### Adeno‐Associated Virus Injection

AAV‐BR1‐ShRNA‐negative Control (Sh‐NC), AAV‐BR1‐ShRNA‐CircPTPN4 (Sh‐CircPTPN4), AAV‐BR1‐ShRNA‐ECE‐1 (Sh‐ECE‐1) were constructed and packaged by Han Bio (Shanghai, China). The ShRNA sequences and CircPTPN4 sequence used in the design process are provided in Tables S4 and S5, Supporting Information. Virus were injected via tail vein to infect cerebrovascular endothelial cells (5 × 10^12^ v. g per mouse). Mice were immobilized with intravenous visual mouse tail injection fixator (YLS‐Q9G, Jinan), and their tails were swabbed with an alcohol cotton ball to dilate the blood vessels. The virus was injected about one‐third of the way from the tip of the tail, and pressure was applied with a cotton ball for 1 min to Control bleeding. Given the minimal damage, no pain medication was administered. The mice then recovered for 2 weeks.

### Lentivirus Vector Construction

The pLO5‐circPTPN4 lentivirus vector (OE‐CircPTPN4) and the pLO5‐ negative Control lentivirus vector (OE‐NC) were constructed by Geneseed (Guangzhou, China). The exons 6, 7, and 8 of the PTPN4 gene were precisely inserted into the pLO5‐ciR lentivirus vector, while a scrambled sequence was employed to create the Control lentivirus vector. The circPTPN4 sequence is listed in Table S5, Supporting Information.

### Cell Transfection

BMECs were cultured on 12‐well plates and experiments were performed when BMECs reaching 70% confluence. For circPTPN4 overexpression, the BMECs were transduced with 1 µg of the OE‐circPTPN4 lentivirus and 1.5 µL of Lipofectamine 2000 (ThermoFisher, 11 668 030) in 500 µL of DMEM for a duration of 48 h. For circPTPN4 knockdown, hU6‐ShRNA‐circPTPN4 lentivirus vector (Sh‐CircPTPN4) was constructed by Genechem (Shanghai, China). BMECs were transduced with Sh‐CircPTPN4 (1.25 × 109TU/500ul) and Polybrene (Beyotime, C0351) in 500 µL of DMEM for 3 days. For miR‐145a‐5p intervention in vitro, BMECs were treated with miR‐145a‐5p mimics or miR‐145a‐5p inhibitors for 2 days, according to the manufacturer's instructions. The miR‐145a‐5p mimics (HY‐R00282), miRNA mimics negative Control (HY‐R04602), miR‐145a‐5p inhibitor (HY‐RI00282), and miRNA inhibitor negative Control (HY‐RI04602) were purchased from Med Chem Express (MCE). The sequences of the miR‐145a‐5p mimic and inhibitor are provided in Table S6, Supporting Information.

### Statistical Analyses

All results are expressed as means ± standard error of the mean (S.E.M.) in the figure legends. At least five independent samples were used in each group and each sample was repeated three times to ensure the reliability. The same brain regions from different mice were considered independent samples. Since the Control group serves as the benchmark in the experiment to assess the relative changes of other groups, this work first calculated the mean value (µControl) of the Control group's data during the data processing phase. Subsequently, all data (including that of the Control group) is compared to µControl so that the Control group data centers ≈1. This adjustment eliminates baseline differences between experiments, allowing the relative changes of the other groups to be expressed with 1 as the reference point. The statistics are evaluated utilizing the “evaluation of outliers” function of GraphPad. The “ROUTE” method can identify one or more outliers, and the parameter Q is set to 1%. In this article, no data were excluded when performing the statistical analyses. Statistical analysis was performed using GraphPad Prism 8.4.2 software. Comparisons between two groups were performed using an unpaired two‐tailed Student's *t*‐test (for homogeneity of variance) or an unpaired two‐tailed Welch's *t*‐test (for heterogeneity of variance). One‐way or two‐way analyses of variance (ANOVA) with Tukey's post hoc test was used for multiple group comparisons. For comparisons between three groups with the heterogeneity of variance, Brown‐Forsythe and Welch's ANOVA was used, followed by Dunnett's T3 post hoc test. The Shapiro–Wilk test was used to assess the normality of each group's distribution. Pearson's correlation coefficient was used to evaluate correlations. Differences were considered statistically significant for *p* < 0.05.

## Conflict of Interest

The authors declare no conflict of interest.

## Author Contributions

X.Z. and J.Y. conceptualized the project. J.Y. designed and performed most of the experiments. Y.H., F.W., X.P., and H.Q. performed or helped in the interpretation and design of some key experiments. Y.Y., C.Z., and L.Z. investigated the project background. J.Y., Y.H., L.Z., X.L., K.X., and C.Z. were involved in the data visualization process. A.Z., C.C., Y.Z., C.Z., and G.G. supervised the study. J.Y. wrote the original draft. X.Z. edited the manuscript.

## Supporting information



Supporting Information

Supplemental Data

## Data Availability

The data that support the findings of this study are available from the corresponding author upon reasonable request. The raw sequencing data was deposited in the NCBI Gene Expression Omnibus (GEO) repository under accession number GSE273352. The dataset is publicly accessible through this permanent link: https://www.ncbi.nlm.nih.gov/geo/query/acc.cgi?acc=GSE273352.
