# Supplementary material for: Circular RNA PTPN4 Contributes to Blood‐Brain Barrier Disruption during Early Epileptogenesis
Source: Adv Sci (Weinh). 2025 Dec 14;13(12):e02250. doi: 10.1002/advs.202502250 (PMC12948252; doi:10.1002/advs.202502250)
Supplement: Supplementary file 1 — Supporting Information [file ADVS-13-e02250-s001.docx]

Supporting Information

Circular RNA PTPN4 Contributes to Blood-Brain Barrier Disruption During Early Epileptogenesis

*Jiurong Yang^1^, Yang Hu^1^, Feiyu Wang^1^, Xintao Peng^1,2^, Honggang Qi^1^, Yuanyuan Yao^1^, Canyu Zhang^1^, Lijie Zhou^1^, Xuemei Liang^1^, Kang Xu^1^, Cong Zhang^1^, Aifeng Zhang^3^, Chen Chen^4^, Yu Zeng^5^, Chenchen Zhang^6^, Guangming Gan^6,7^ and Xinjian Zhu*********^1^*

**
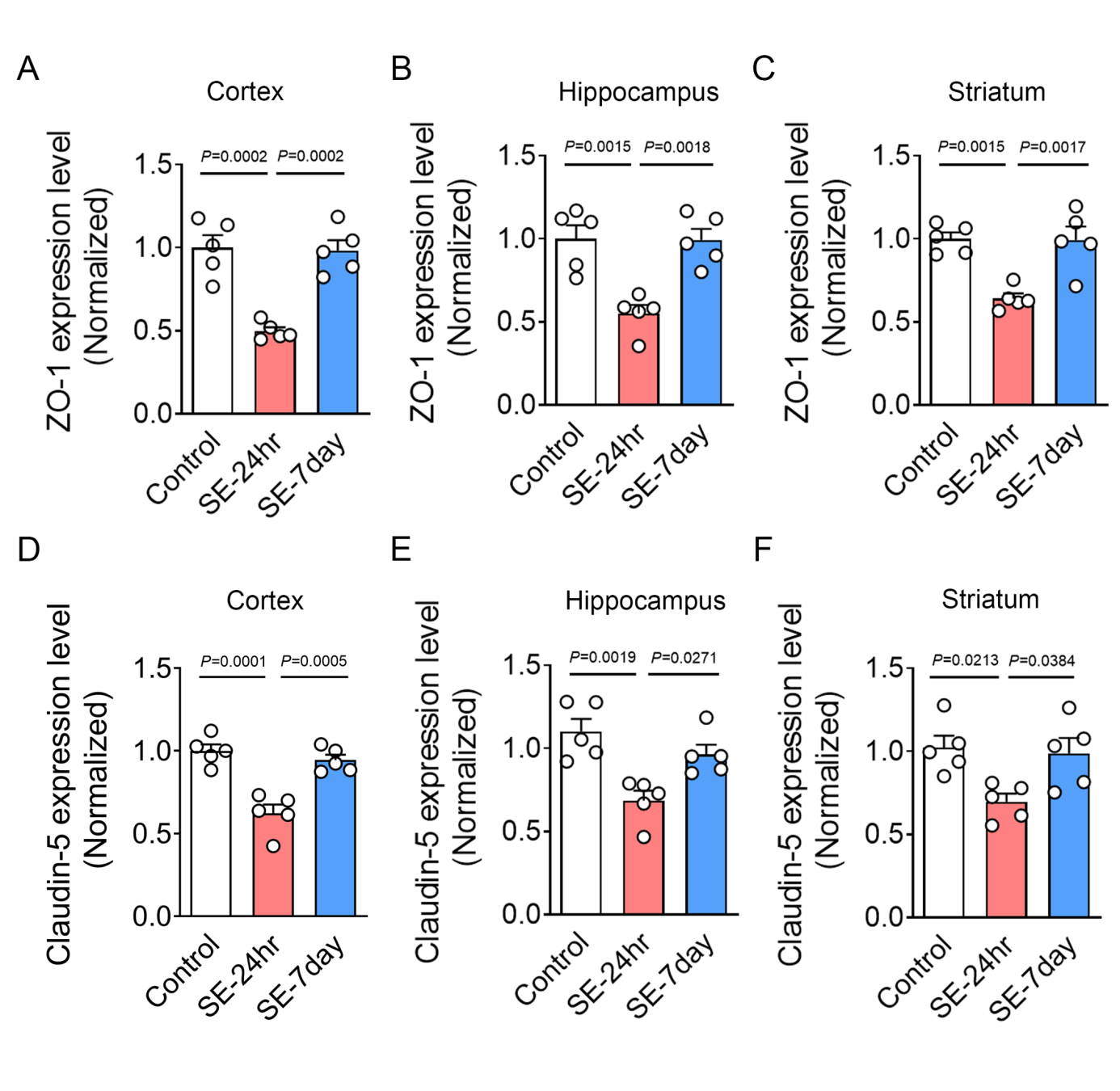
**

**Figure S1.** Regional downregulation of tight junction proteins ZO-1 and Claudin-5 during early epileptogenesis. A-C) Quantative RT-PCR analyses of ZO-1 expression in the cortex (n=5; p=0.0002, SE-24h vs Control; p=0.0002, SE-7day vs SE-24h), hippocampus (n=5; p=0.0015, SE-24h vs Control; p=0.0018, SE-7day vs SE-24h), and striatum (n=5; p=0.0015, SE-24h vs Control; p=0.0017, SE-7day vs SE-24h) at 24 hours and 7days after SE. D-F) Quantative RT-PCR analyses of claudin-5 expression in the cortex (n=5; p=0.0001, SE-24h vs Control; p=0.0005, SE-7day vs SE-24h), hippocampus (n=5; p=0.0019, SE-24h vs Control; p=0.0217, SE-7day vs SE-24h), and striatum (n=5; p=0.0213, SE-24h vs Control; p=0.0384, SE-7day vs SE-24h) at 24 hours and 7days after SE. Data are presented as means ± S.E.M. Statistical significance was determined by one-way ANOVA followed by Tukey’s post hoc test.

**
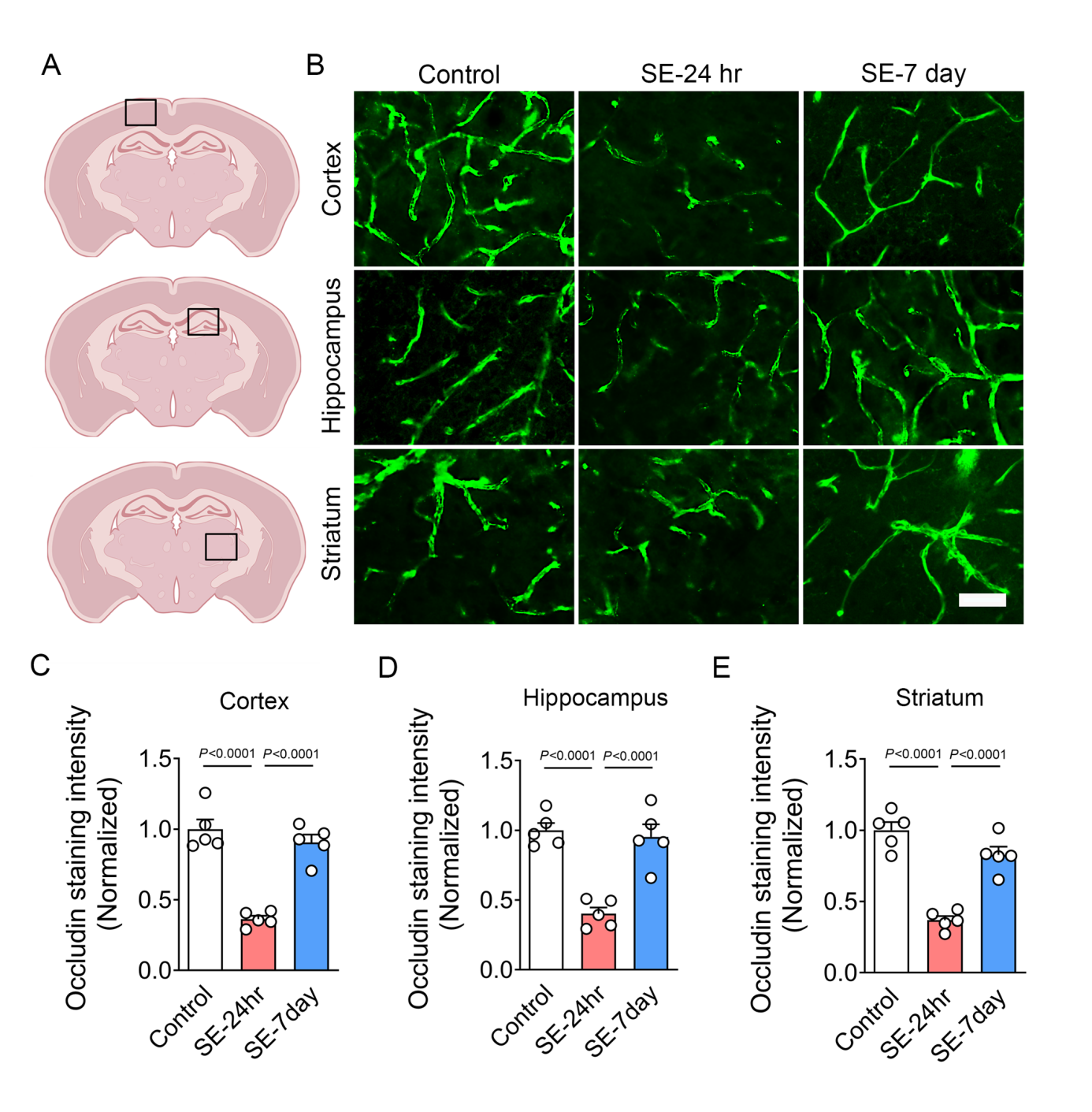
**

**Figure S2.** Regional downregulation of tight junction protein Occludin during early epileptogenesis. A) Schematic coronal section showing analyzed brain regions (boxed: cortex, hippocampus, striatum). B) Representative images of Occludin immunofluorescence in the cortex, hippocampus, and striatum of Control, SE-24h, and SE-7day mice. C-E) Bar graph displaying the quantification of mean Occludin fluorescence intensity in the cortex (n=5; p<0.0001, SE-24h vs Control; p<0.0001, SE-7day vs SE-24h), hippocampus (n=5; p<0.0001, SE-24h vs Control; p<0.0001, SE-7day vs SE-24h), and striatum (n=5; p<0.0001, SE-24h vs Control; p<0.0001, SE-7day vs SE-24h). Data are presented as means ± S.E.M. Statistical analyses were performed using one-way ANOVA followed by Tukey’s post hoc test. Scale bar=25 μm.

**
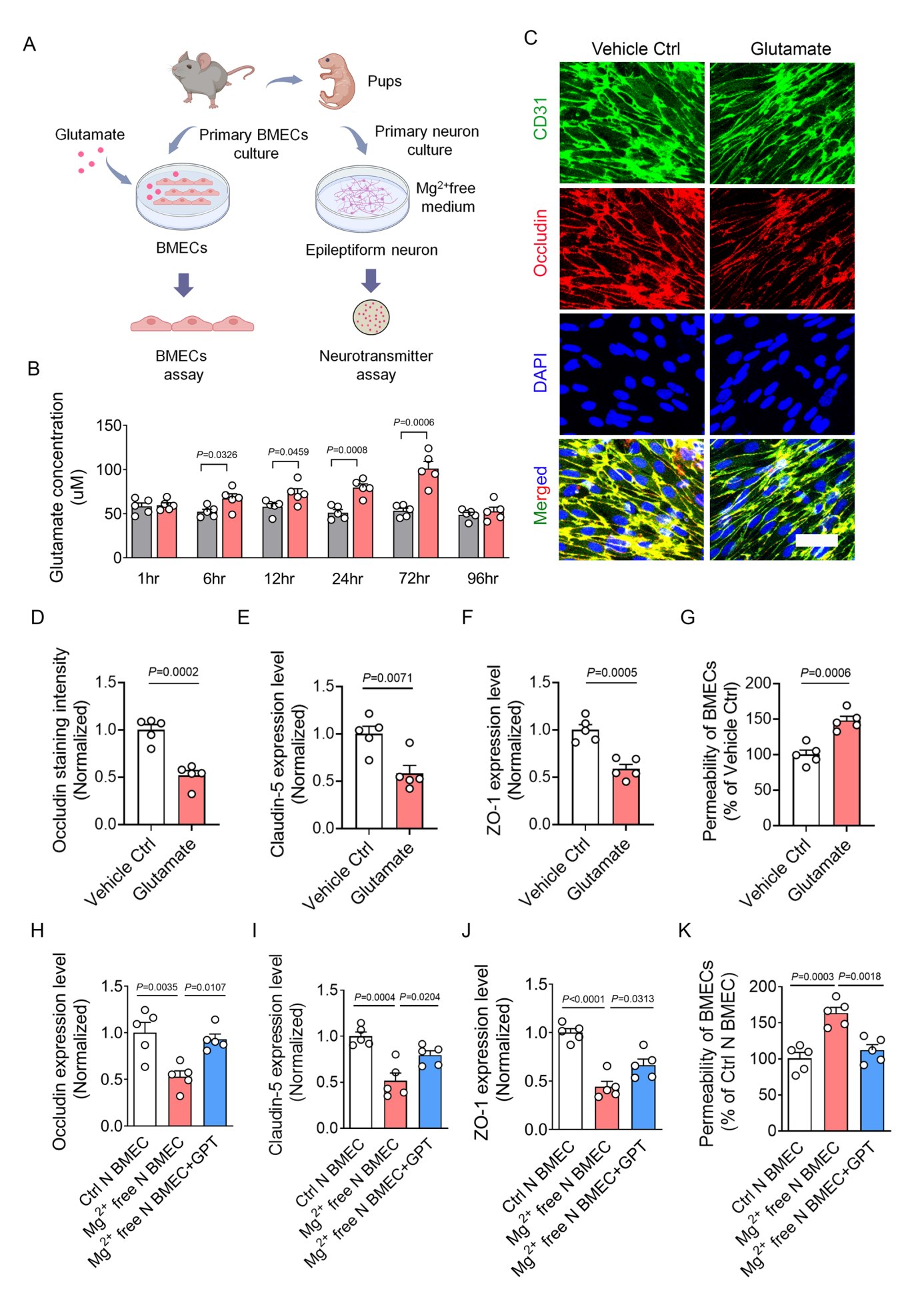
**

**Figure S3.** Glutamate-dependent disruption of endothelial tight junctions in epileptic conditions. A) Schematic representation of tight junction detection in primary BMECs following glutamate exposure and the neurotransmitter release assay in free Mg²⁺-induced epileptiform neuronal cultures. B) Glutamate release measured at various time points in free Mg²⁺-induced epileptiform neuronal culture medium (n=5; p=0.0326 at 6 h, p=0.0459 at 12 h, p=0.0008 at 24 h, and p=0.0006 at 72 h). C) Representative images of CD31/Occludin co-immunostaining in vehicle Control and glutamate-treated BMECs. D) Quantification of the mean fluorescence intensity of Occludin staining in vehicle Control and glutamate-treated BMECs (n=5, p=0.0002, Glutamate vs vehicle Control). E, F) Quantative RT-PCR analyses of claudin-5 (n=5, p=0.0071, Glutamate vs vehicle Control) and ZO-1 (n=5, p=0.0005, Glutamate vs vehicle Control) expression in Control versus glutamate-treated BMECs. G) Permeability assay using Na-F assay in vehicle Control versus glutamate-treated BMECs (n=5, p=0.0006, Glutamate vs vehicle Control). H-J) Quantative RT-PCR analyses of Occludin (n=5; p=0.0035, Mg²⁺-free neuron co-cultured BMEC vs Control; p=0.0107, Mg²⁺-free neuron co-cultured BMEC + GPT vs Mg²⁺-free neuron co-cultured BMEC), claudin-5 (n=5; p=0.0004, Mg²⁺-free neuron co-cultured BMEC vs Control; p=0.0204, Mg²⁺-free neuron co-cultured BMEC + GPT vs Mg²⁺-free neuron co-cultured BMEC), and ZO-1 expression (n=5; p<0.0001, Mg²⁺-free neuron co-cultured BMEC vs Control; p=0.0313, Mg²⁺-free neuron co-cultured BMEC + GPT vs Mg²⁺-free neuron co-cultured BMEC). (K) Permeability analyses using Na-F assay in Control (Ctrl N BMEC), Mg²⁺-free neuron co-cultured BMEC (Mg²⁺-free N BMEC), and Mg²⁺-free neuron co-cultured BMEC treated with GPT (Mg²⁺-free N BMEC + GPT) (n=5; p=0.0003, Mg²⁺-free N BMEC vs Ctrl N BMEC; p=0.0018, Mg²⁺-free N BMEC + GPT vs Mg²⁺-free N BMEC). Values are presented as means ± S.E.M. Statistical analysis include unpaired two-tailed Student’s t-test (B, D, E, F, G), one-way ANOVA followed by Tukey’s post hoc test (H, I, J, K). Scale bar=25 μm.

**
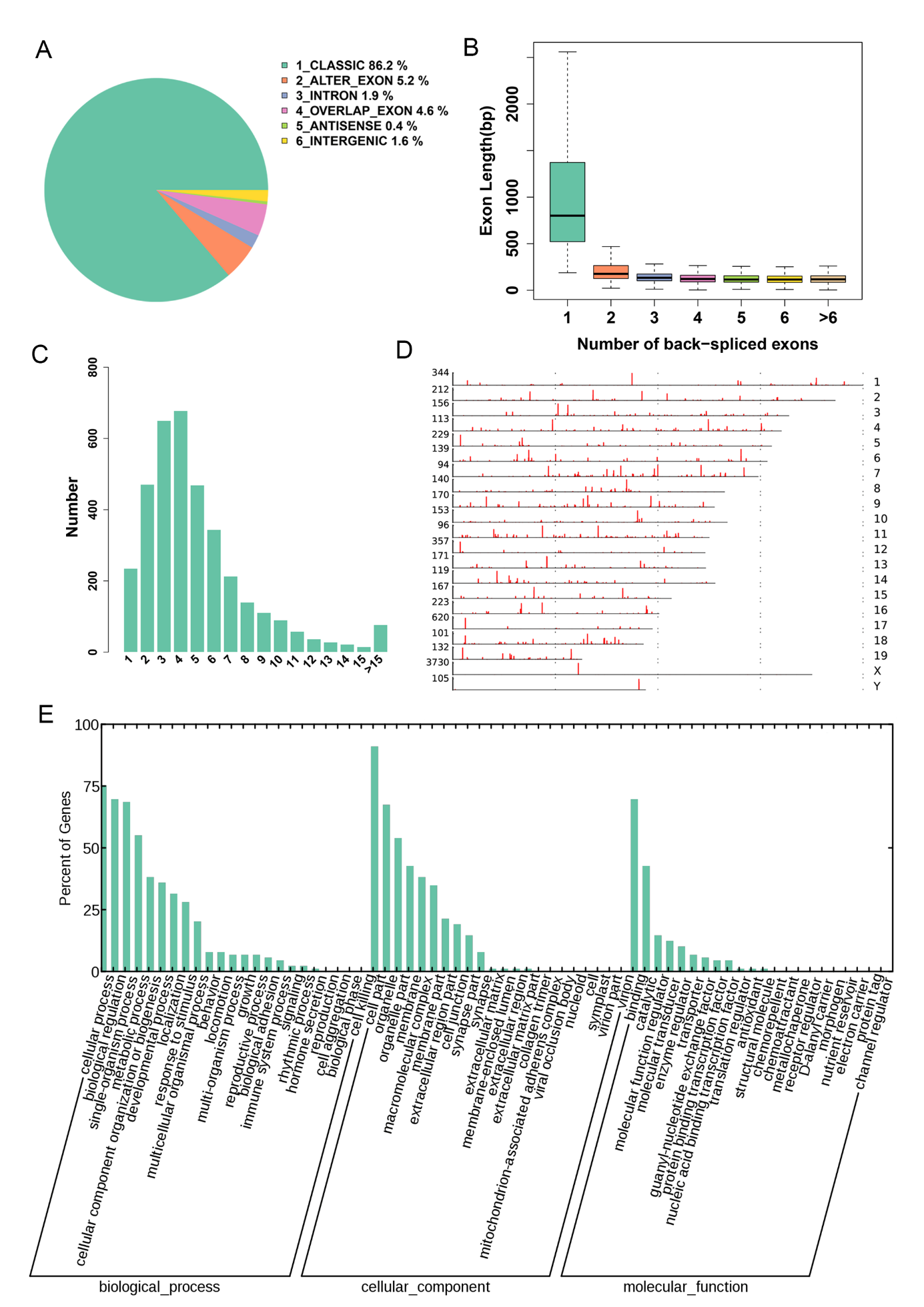
**

**Figure S4.** Bioinformatics analyses of cortical circRNAs detected by microarray in TLE mice. A) Pie chart illustrating the proportion of different types of circular RNAs identified in the microarray. B) Box-and-whisker plot depicting the exon lengths of single- and multiple-exon-derived circRNAs. C) Bar graph showing the number of circular RNAs with varying numbers of exons. D) Distribution of the start and end positions of circular RNAs across different chromosomes. E) Gene Ontology (GO) analyses of genes derived from circRNAs.

**
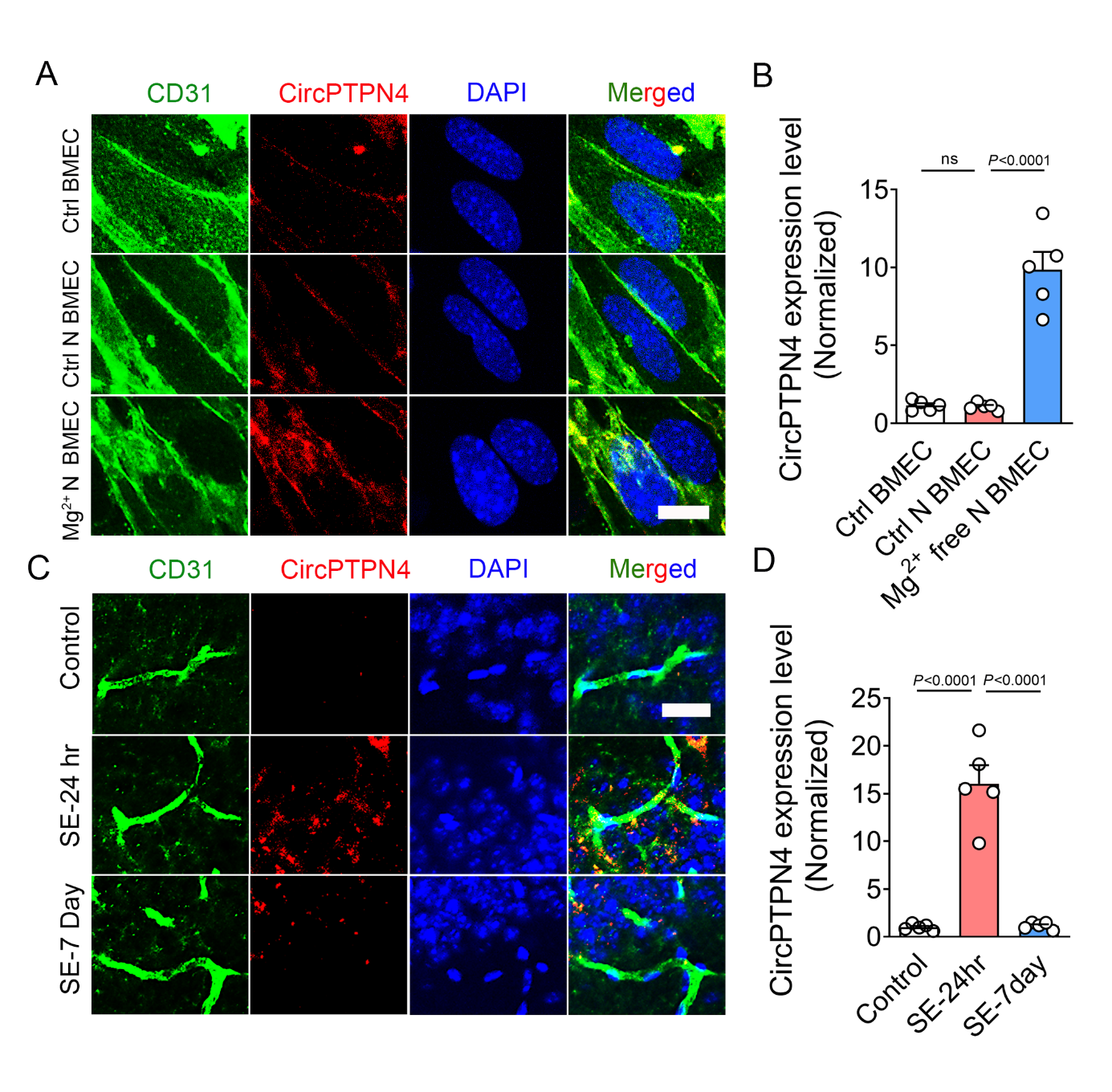
**

**Figure S5.** CircPTPN4 location and expression in endothelial cells *in vitro* and *in vivo* under epileptic conditions. A) Representative images showing the location of CircPTPN4 in Ctrl BMEC, Ctrl N BMEC, and Mg²⁺ free N BMEC. BMECs were stained with CD31. B) Quantative RT-PCR analyses of CircPTPN4 expression in Ctrl BMECs, Ctrl N BMEC and Mg²⁺ free N BMECs (n=5; ns, Ctrl N BMEC vs Ctrl BMEC; p<0.0001, Mg²⁺ free N BMEC vs Ctrl N BMEC). C) Representative images showing the location of CircPTPN4 in endothelial cells of Control, SE-24h, and SE-7day mice. Endothelial cells were stained with CD31. D) Quantative RT-PCR analyses of CircPTPN4 expression in the cortex of Control, SE-24h, and SE-7day mice (n=5; p<0.0001, SE-24h vs Control; p<0.0001, SE-7day vs SE-24h). Data are presented as means ± S.E.M. Statistical analyses was performed using one-way ANOVA followed by Tukey’s post hoc test. Scale bar=10 μm in (A) and 25 μm in (C).

**
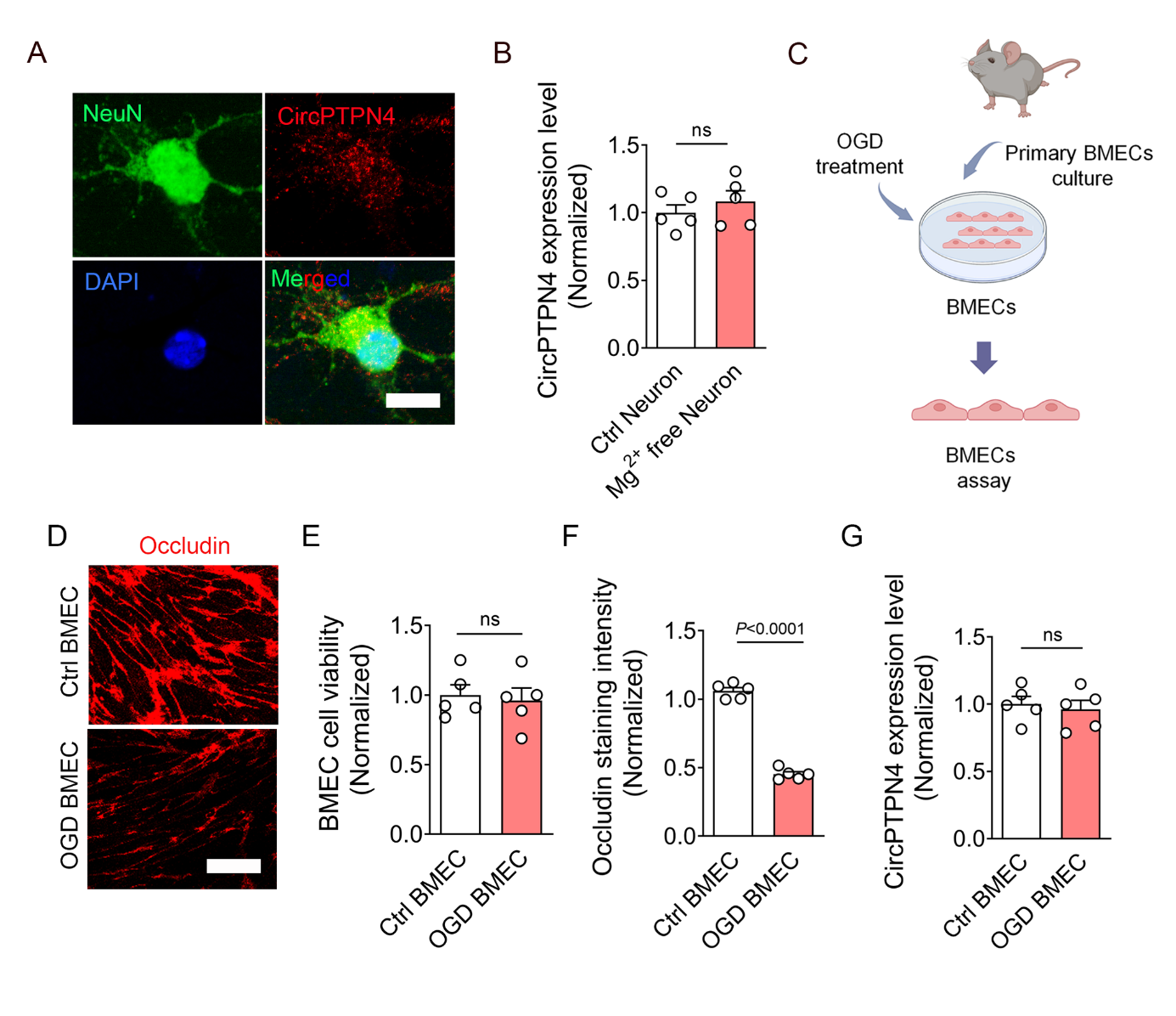
**

**Figure S6.** CircPTPN4 is specifically upregulated in BMECs under epileptic conditions. A) Representative images showing the location of CircPTPN4 in neurons. Neurons were stained with NeuN. B) Quantative RT-PCR analyses of CircPTPN4 expression in Control and Mg²⁺-free neurons (n=5; ns; Ctrl neuron vs Mg^2+^ free neuron). C) Schematic diagram illustrating that primary BMECs were exposed to OGD treatment, followed by a tight junction assay. D) Representative images of Occludin immunostaining in Control and OGD-treated BMECs. E) Bar graph showing the results of a cell viability assessment of Control and OGD-treated BMECs (n=5, ns, Ctrl BMEC vs OGD BMEC). F) Bar graph quantifying the mean fluorescence intensity of Occludin in Control and OGD-treated BMECs (n=5, p<0.0001, OGD BMEC vs Ctrl BMEC). G) Quantative RT-PCR analyses of CircPTPN4 levels in Control and OGD-treated BMECs (n=5, ns, OGD BMEC vs Ctrl BMEC). Values are expressed as means ± S.E.M. Statistical analyses were performed using unpaired two-tailed Student’s t-test. Scale bar = 10 µm (A) and 25 µm (D).

**
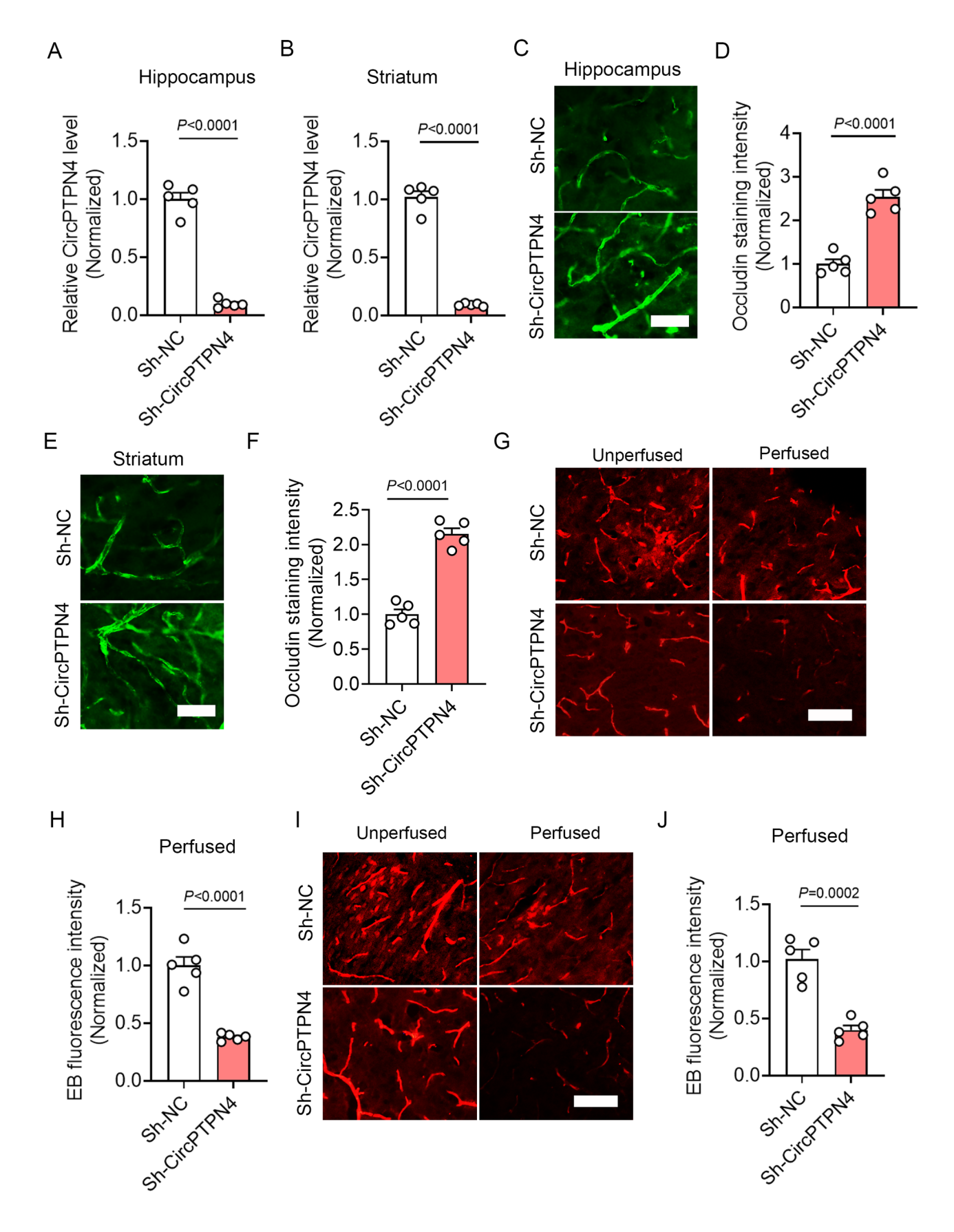
**

**Figure S7.** Knockdown of CircPTPN4 ameliorates BBB damage in the hippocampus and striatum. A, B) Quantative RT-PCR analyses confirming the efficiency of CircPTPN4 knockdown in the hippocampus (n=5, p<0.0001, Sh-CircPTPN4 vs Sh-NC) and striatum (n=5, p<0.0001, Sh-CircPTPN4 vs Sh-NC) of Sh-NC- and Sh-CircPTPN4-treated SE-24h mice. C) Representative images of Occludin immunostaining in the hippocampus of Sh-NC- and Sh-CircPTPN4-treated SE-24h mice. D) Bar graph quantifying the mean fluorescence intensity of Occludin staining in the hippocampus (n=5, p<0.0001, Sh-CircPTPN4 vs Sh-NC). E) Representative images of Occludin immunostaining in the striatum of Sh-NC- and Sh-CircPTPN4-treated SE-24h mice. F) Bar graph quantifying the mean fluorescence intensity of Occludin staining in the striatum (n=5, p<0.0001, Sh-CircPTPN4 vs Sh-NC). G) EB fluorescence detected in the hippocampus of Sh-CircPTPN4- and Sh-NC-treated mice before and after perfusion. H) Bar graph quantifying the mean fluorescence intensity of EB in the hippocampus of Sh-CircPTPN4- and Sh-NC-treated mice after perfusion (n=5, p<0.0001, Sh-CircPTPN4 vs Sh-NC). I) EB fluorescence detected in the striatum of Sh-CircPTPN4- and Sh-NC-treated mice before and after perfusion. J) Bar graph quantifying the mean fluorescence intensity of EB in the striatum of Sh-CircPTPN4- and Sh-NC-treated mice after perfusion (n=5, p=0.0002, Sh-CircPTPN4 vs Sh-NC). Values are expressed as means ± S.E.M. Statistical analysis were performed using unpaired two-tailed Student’s t-test. Scale bar =25µm.

**
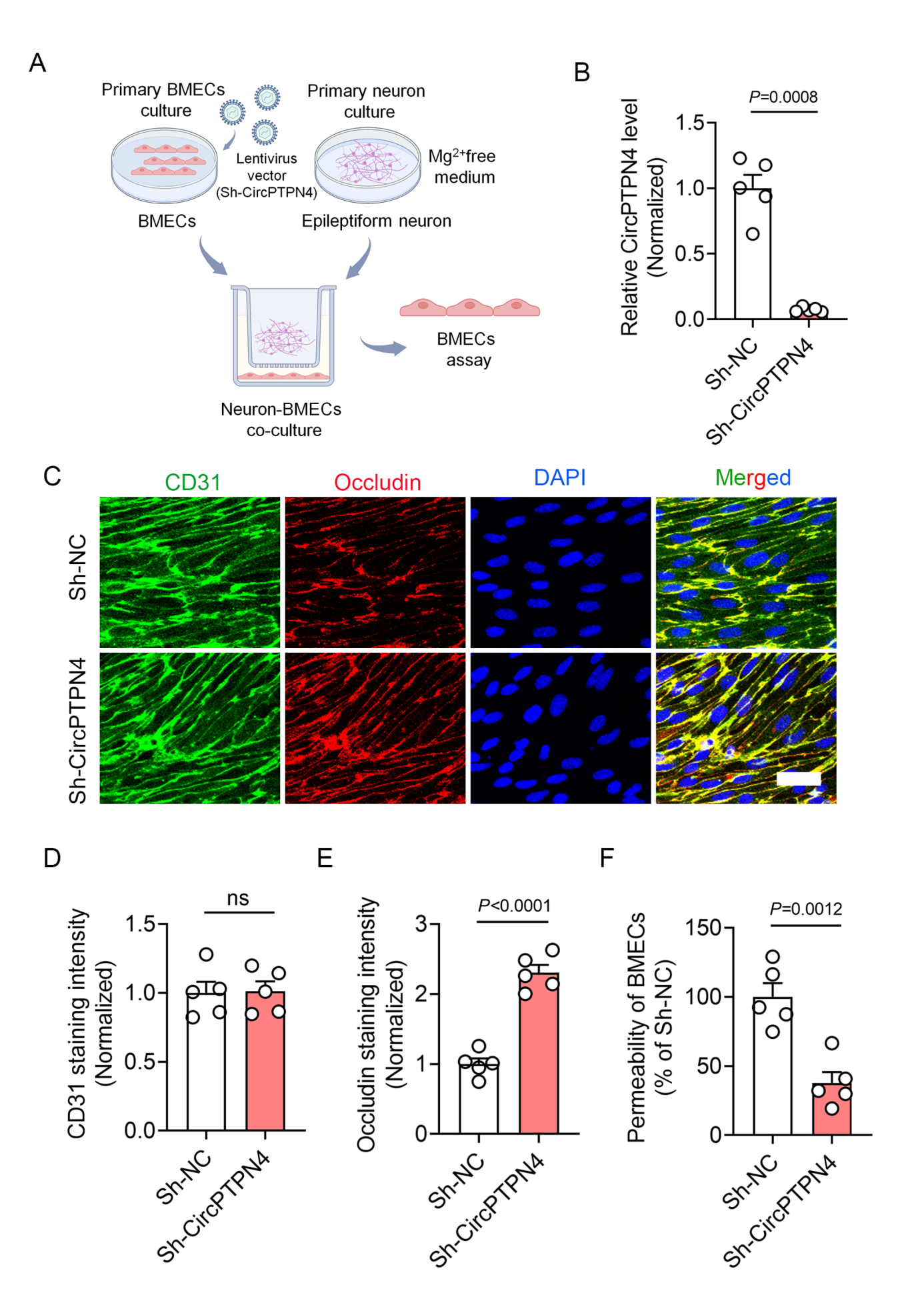
**

**Figure S8.** Knockdown of CircPTPN4 ameliorates epilepsy-induced BMEC tight junction damage. A) Schematic diagram illustrating the co-culture of Sh-CircPTPN4-treated BMECs with free Mg²⁺-induced epileptiform neurons, followed by tight junction assay. B) Quantative RT-PCR analyses validating CircPTPN4 levels in Sh-NC- and Sh-CircPTPN4-treated BMECs co-cultured with epileptiform neurons (n=5, p=0.0008, Sh-CircPTPN4 vs Sh-NC). C) Representative images of CD31/Occludin immunostaining in Sh-NC- and Sh-CircPTPN4-treated BMECs co-cultured with epileptiform neurons. D, E) Bar graph quantifying the mean fluorescence intensity of CD31 (n=5, ns, Sh-CircPTPN4 vs Sh-NC) and Occludin staining (n=5; p<0.0001, Sh-CircPTPN4 vs Sh-NC). F) Bar graph showing the permeability of BMECs in Sh-NC- and Sh-CircPTPN4-treated BMECs co-cultured with epileptiform neurons (n=5; p=0.0012, Sh-CircPTPN4 vs Sh-NC). Data are presented as means ± S.E.M. Statistical analyses was performed using an unpaired two-tailed Student’s t-test (D, E, F) and an unpaired two-tailed Welch’s t-test (B). Scale bar=25 μm.

**
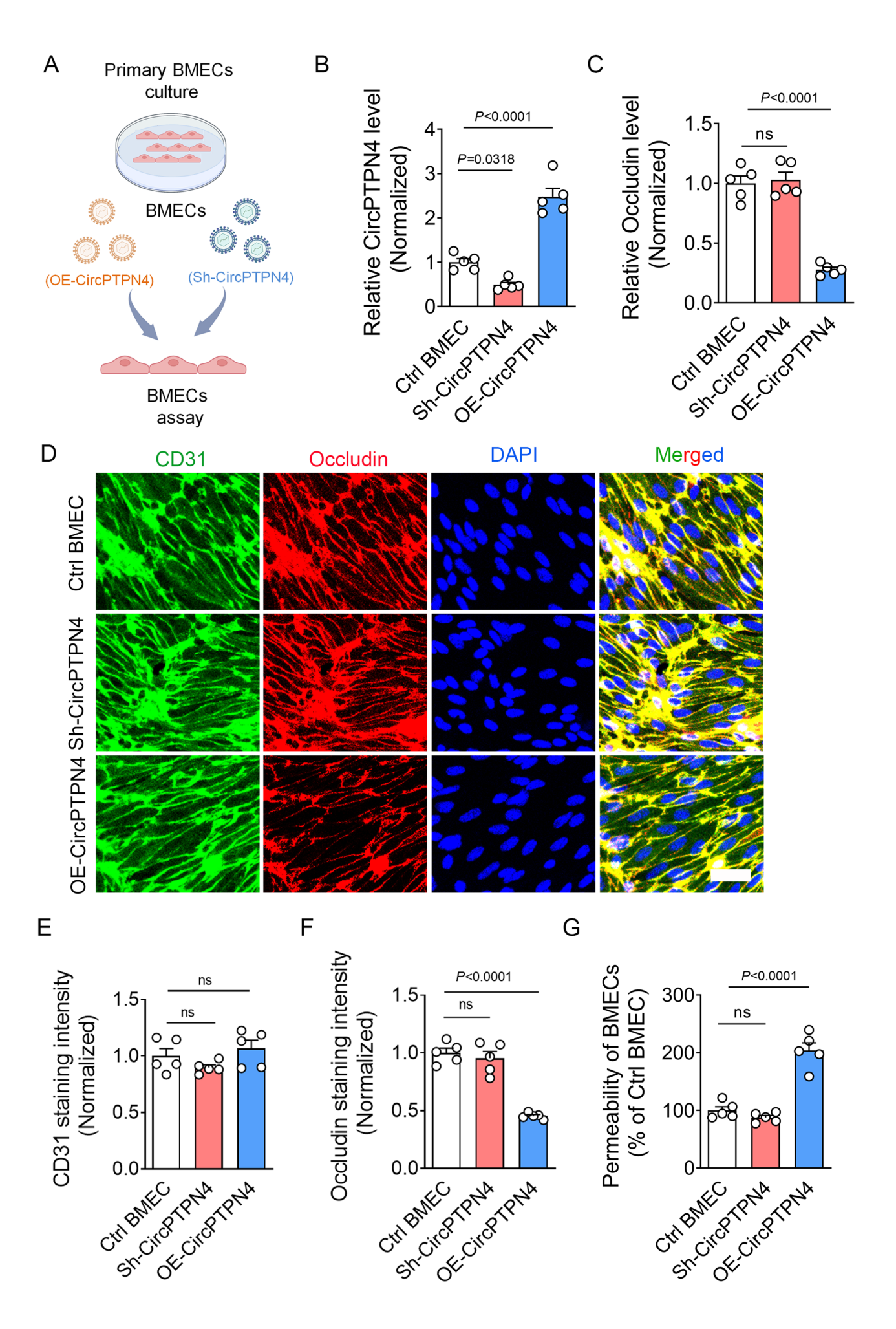
**

**Figure S9.** Effect of CircPTPN4 on tight junctions of normal BMECs. A) Schematic of the experimental design: Primary BMECs were infected with lentivirus carrying either OE-CircPTPN4 or Sh-CircPTPN4, followed by a tight junction assay. B) Quantative RT-PCR analyses of CircPTPN4 overexpression (OE-CircPTPN4) and knockdown (Sh-CircPTPN4) efficiency (n=5; p=0.0318, Sh-CircPTPN4 vs Control; p<0.0001, OE-CircPTPN4 vs Control). C) Quantative RT-PCR analyses of Occludin in Control, Sh-CircPTPN4, and OE-CircPTPN4-treated BMECs (n=5; ns, Sh-CircPTPN4 vs Control; p<0.0001, OE-CircPTPN4 vs Control). D) Representative images of CD31/Occludin co-immunostaining in Control, Sh-CircPTPN4, and OE-CircPTPN4-treated BMECs. E, F) Quantification of the mean fluorescence intensity of CD31 (n=5; ns, Sh-CircPTPN4 vs Control; ns, OE-CircPTPN4 vs Control) and Occludin (n=5; ns, Sh-CircPTPN4 vs Control; p<0.0001, OE-CircPTPN4 vs Control) in Control, Sh-CircPTPN4-, and OE-CircPTPN4-treated BMECs. G) Bar graph showing the permeability of BMECs, as measured by the Na-F assay, in Control, Sh-CircPTPN4-, and OE-CircPTPN4-treated BMECs (n=5; ns, Sh-CircPTPN4 vs Control; p<0.0001, OE-CircPTPN4 vs Control). Data are presented as means ± S.E.M. Statistical analysis were performed using one-way ANOVA followed by Tukey's post hoc test. Scale bar=25 µm.

**
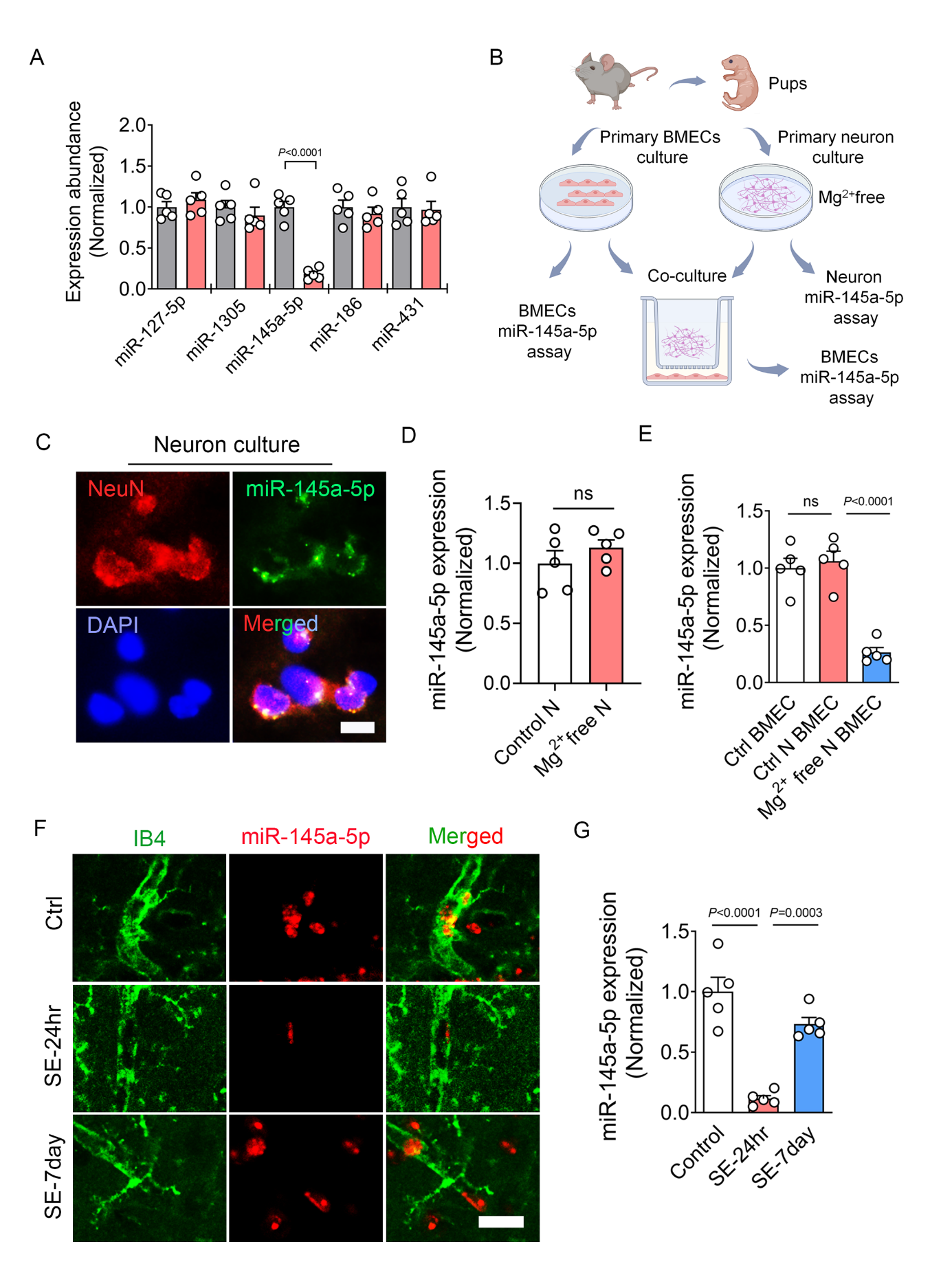
**

**Figure S10.** Cell-type-specific regulation of miR-145a-5p in vascular endothelia under epileptic condition. A) Quantative RT-PCR analyses confirmed significant changes in miR-145a-5p expression among the top five predicted CircPTPN4-targeted miRNAs at 24 hours post-SE. B) Schematic diagram illustrating the co-culture of BMECs with free Mg²⁺-induced epileptiform neurons, followed by miR-145a-5p assay. Samples of Ctrl BMECs, Ctrl/Mg^2+^ free Neurons and Ctrl N/Mg^2+^ free N BMECs were collected for miR-145a-5p assay. C) Representative images showing miR-145a-5p location in neurons. Neurons were stained with NeuN. D) Quantative RT-PCR analyses of miR-145a-5p expression in Control and free Mg²⁺-treated neurons (n=5, ns, Mg²⁺ free N vs Control N). E) Quantative RT-PCR analyses of miR-145a-5p expression in Ctrl BMEC, Ctrl N BMEC, and Mg²⁺ free N BMEC (n=5; ns, Ctrl N BMEC vs Ctrl BMEC; p<0.0001, Mg²⁺ free N BMEC vs Ctrl N BMEC). F) Representative images showing miR-145a-5p expression in endothelial cells of Control, SE-24h, and SE-7day mice. Endothelial cells were marked with IB4. G) Quantative RT-PCR analyses of miR-145a-5p expression in the cortex of Control, SE-24h, and SE-7day mice (n=5; p<0.0001, SE-24h vs Control; p=0.0003, SE-7day vs SE-24h). Values are presented as means ± S.E.M. Statistical analysis were performed using one-way ANOVA followed by Tukey’s post hoc test (E, G) and unpaired two-tailed student’s t-test (A, D). Scale bar = 10µm.

**
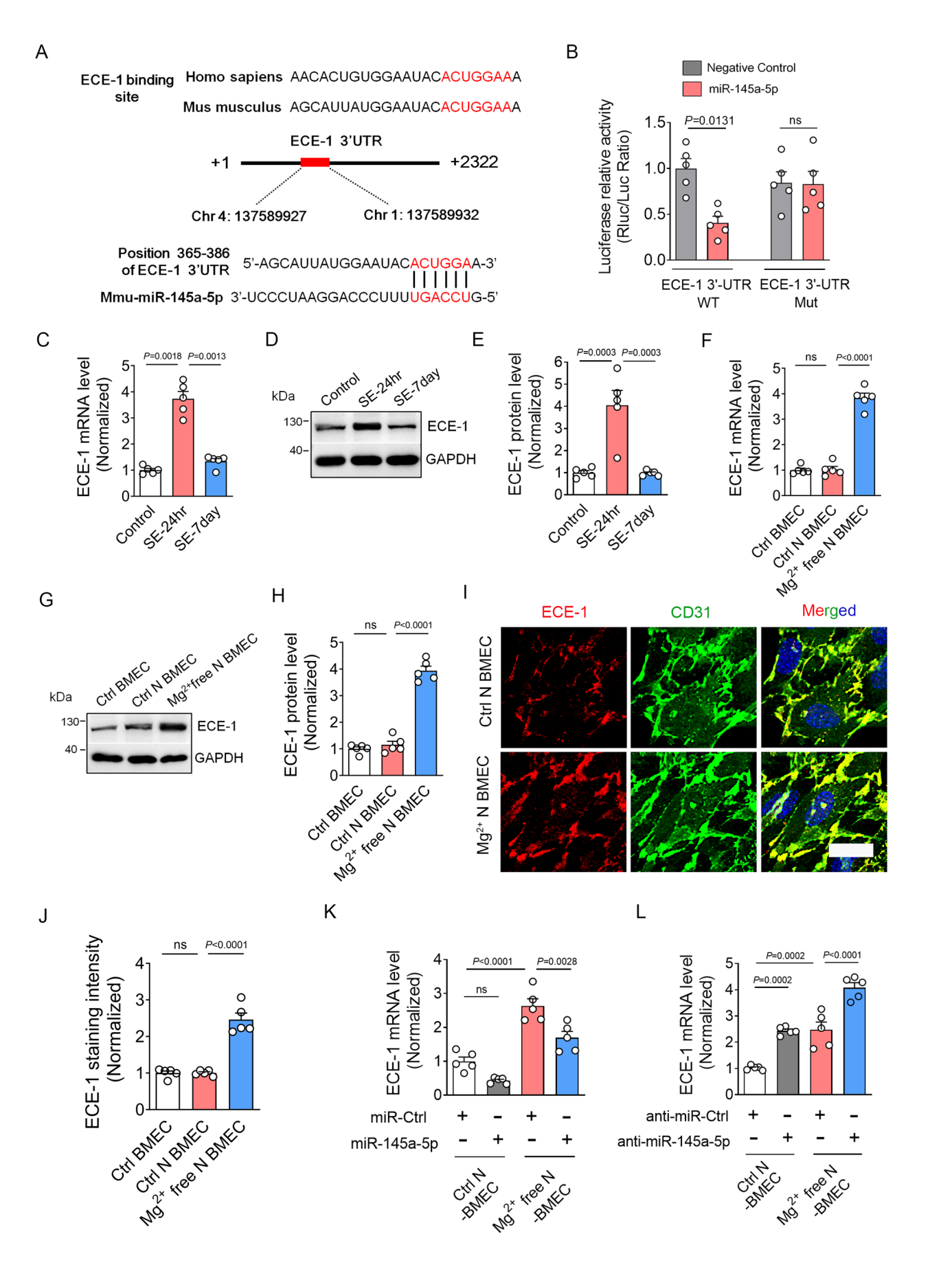
**

**Figure S11.** MiR-145a-5p targets ECE-1 and regulates its expression. A) Schematic diagram showing the binding sites between ECE-1 mRNA and miR-145a-5p. B) Luciferase activity analyses of wild-type (WT) and 3’-UTR mutant constructs of ECE-1 mRNA (n=5, p=0.0131, ECE-1 3’UTR WT Negative Control vs ECE-1 3’UTR WT miR-145a-5p). C) Quantative RT-PCR analyses of ECE-1 mRNA expression in the cortex of Control, SE-24h, and SE-7day mice (n=5; p=0.0018, SE-24h vs Control group; p=0.0013, SE-7day vs SE-24h group). D) Western blot analyses showing the protein levels of ECE-1 in the cortex of Control, SE-24h, and SE-7day mice. E) Bar graph showing the quantification of ECE-1 protein levels, represented as the intensity ratio of ECE-1 to GAPDH (n=5; p=0.0003, SE-24h vs Control group; p=0.0003, SE-7day vs SE-24h group). F) Quantative RT-PCR analyses of ECE-1 mRNA expression in Control BMECs, Control neuron co-cultured BMECs, and Mg²⁺-free neuron co-cultured BMECs (n=5; ns, Ctrl N BMEC vs Ctrl BMEC; p<0.0001, Mg²⁺-free N BMEC vs Ctrl N BMEC). G) Western blot analyses showing the protein levels of ECE-1 in Control BMECs, Control neuron co-cultured BMECs, and Mg²⁺-free neuron co-cultured BMECs. H) Bar graph showing the quantification of ECE-1 protein levels, represented as the intensity ratio of ECE-1 to GAPDH (n=5; ns, Ctrl N BMEC vs Ctrl BMEC; p<0.0001, Mg²⁺-free N BMEC vs Ctrl N BMEC). I) Representative images of CD31/ECE-1 co-immunostaining in Control neuron co-cultured BMECs and Mg²⁺-free neuron co-cultured BMECs. J) Bar graph showing the quantification of ECE-1 immunostaining in Control BMECs, Control neuron co-cultured BMECs, and Mg²⁺-free neuron co-cultured BMECs (n=5; ns, Ctrl N BMEC vs Ctrl BMEC; p<0.0001, Mg²⁺-free N BMEC vs Ctrl N BMEC). K) Quantative RT-PCR analyses of ECE-1 mRNA expression in Control neuron co-cultured and Mg²⁺-free neuron co-cultured BMECs transfected with Control or miR-145a-5p mimics (n=5; ns, mimic miR-145a-5p-treated Ctrl N BMEC vs Control miRNA-treated Ctrl N BMEC; p=0.0028, mimic miR-145a-5p-treated Mg²⁺-free N BMEC vs Control miRNA-treated Mg²⁺-free N BMEC; p<0.0001, Control miRNA-treated Mg²⁺-free N BMEC vs Control miRNA-treated Ctrl N BMEC). L) Quantative RT-PCR analyses of ECE-1 mRNA expression in Control neuron co-cultured and Mg²⁺-free neuron co-cultured BMECs transfected with Control or miR-145a-5p inhibitors (n=5; p=0.0002, anti-miR-145a-5p-treated Ctrl N BMEC vs Control anti-miRNA-treated Ctrl N BMEC; p=0.0002, anti-miR-145a-5p-treated Mg²⁺-free N BMEC vs Control anti-miRNA-treated Mg²⁺-free N BMEC; p<0.0001, Control anti-miRNA-treated Mg²⁺-free N BMEC vs Control anti-miRNA-treated Ctrl N BMEC). Data are expressed as means ± S.E.M. Statistical analyses was performed using two-way ANOVA followed by Tukey’s test (B), one-way ANOVA followed by Tukey’s test (E, F, H, J, K, L), Brown-Forsythe and Welch's ANOVA with Dunnett T3 post hoc test (C). Scale bar=10 μm.

**
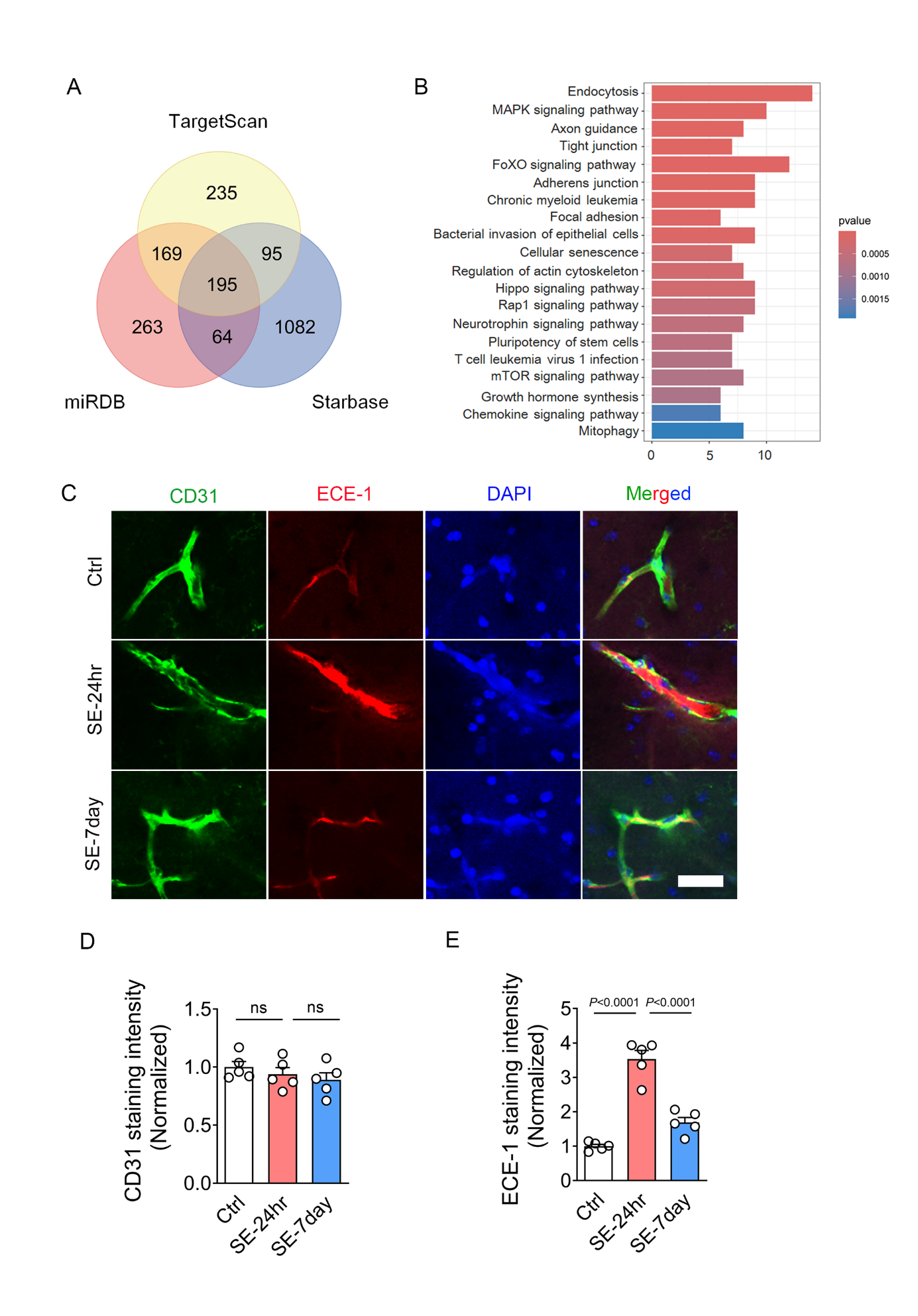
**

**Figure S12.** Expression of miR-145a-5p target gene ECE-1 in cortical vascular endothelial cells. A) A Venn diagram displaying 195 intersecting target genes predicted by the TargetScan, miRDB, and Starbase databases. B) Enrichment analyses of the KEGG pathway. Statistical significance was defined as an adjusted p-value<0.05. C) Representative images of CD31/ECE-1 co-immunostaining in the cortex of Control, SE-24h, and SE-7day mice. D, E) Bar graphs depicting the quantification of mean fluorescence intensity for CD31 (n = 5; ns, SE-24h vs Control; ns, SE-7day vs SE-24h) and ECE-1 (n=5; p<0.0001, SE-24h vs Control; p<0.0001, SE-7day vs SE-24h). Data are presented as means ± S.E.M., with statistical analyses performed using one-way ANOVA followed by Tukey’s post hoc test. Scale bar = 25 μm.

**
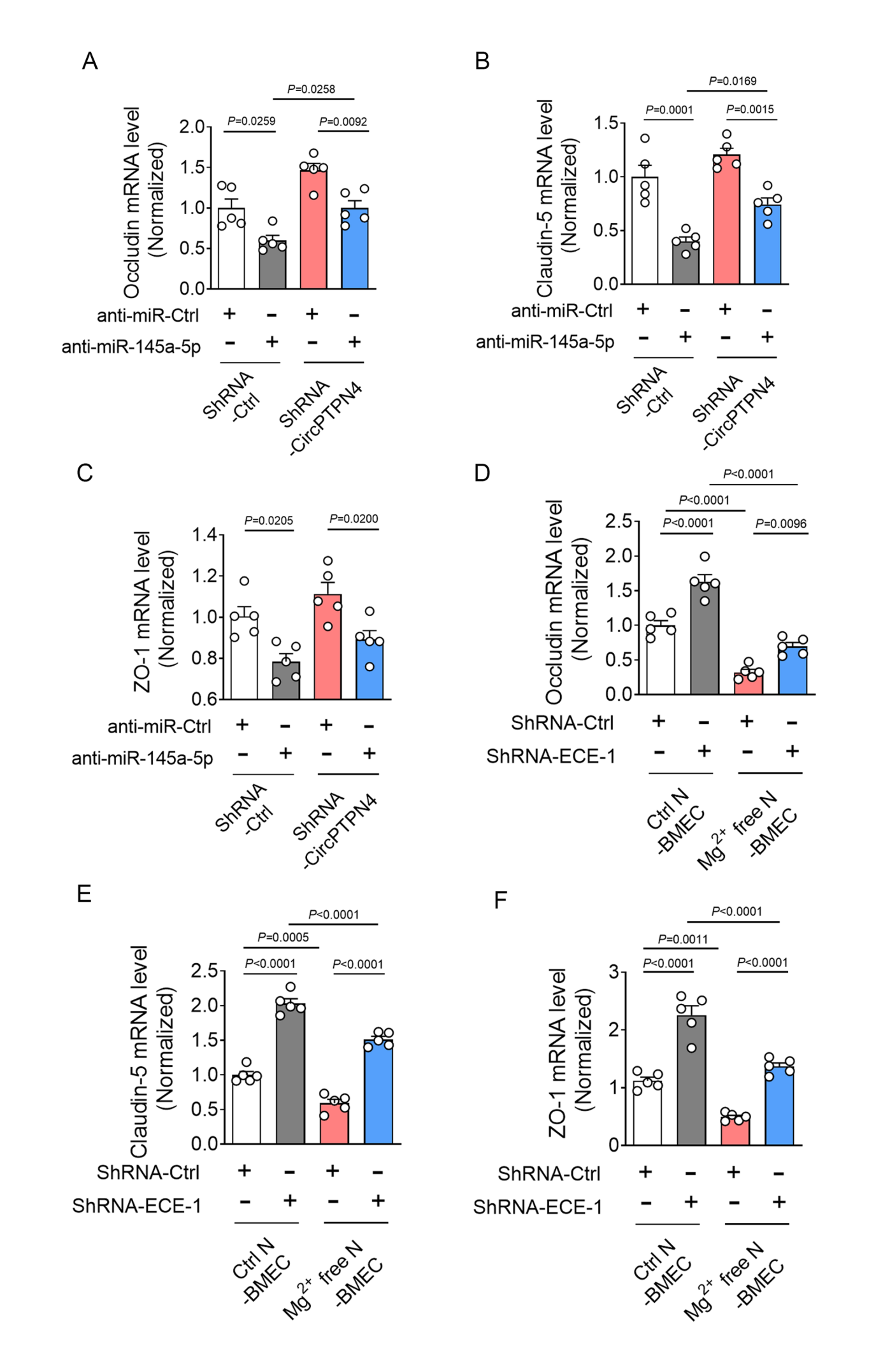
**

**Figure S13.** CircPTPN4 regulates the expression of tight junction proteins Occludin, claudin-5, and ZO-1 via miR-145a-5p. A-C) Quantative RT-PCR analyses of the expression levels of tight junction proteins Occludin (n=5; p=0.0259, anti-miR-145a-5p and ShRNA-Ctrl-treated BMECs vs Control anti-miRNA and ShRNA-Ctrl-treated BMECs; p=0.0092, anti-miR-145a-5p and ShRNA-CircPTPN4-treated BMECs vs Control anti-miRNA and ShRNA-CircPTPN4-treated BMECs; p=0.0258, anti-miR-145a-5p and ShRNA-CircPTPN4-treated BMECs vs anti-miR-145a-5p and ShRNA-Ctrl-treated BMECs), claudin-5 (n=5; p=0.0001, anti-miR-145a-5p and ShRNA-Ctrl-treated BMECs vs Control anti-miRNA and ShRNA-Ctrl-treated BMECs; p=0.0015, anti-miR-145a-5p and ShRNA-CircPTPN4-treated BMECs vs Control anti-miRNA and ShRNA-CircPTPN4-treated BMECs; p=0.0169, anti-miR-145a-5p and ShRNA-CircPTPN4-treated BMECs vs anti-miR-145a-5p and ShRNA-Ctrl-treated BMECs), and ZO-1 (n=5; p=0.0205, anti-miR-145a-5p and ShRNA-Ctrl-treated BMECs vs Control anti-miRNA and ShRNA-Ctrl-treated BMECs; p=0.0200, anti-miR-145a-5p and ShRNA-CircPTPN4-treated BMECs vs Control anti-miRNA and ShRNA-CircPTPN4-treated BMECs) in ShRNA-Ctrl- and ShRNA-CircPTPN4-lentivirus-treated BMECs, which were co-treated with either Control anti-miRNA or anti-miR-145a-5p inhibitors. D-F) Quantative RT-PCR analyses of tight junction proteins Occludin (n=5; p<0.0001, ShRNA-ECE-1-transduced Ctrl N BMECs vs ShRNA-Ctrl-transduced Ctrl N BMECs; p=0.0096, ShRNA-ECE-1-transduced Mg^2+^-free N BMECs vs ShRNA-Ctrl-transduced Mg^2+^-free N BMECs; p<0.0001, ShRNA-Ctrl-transduced Mg^2+^-free N BMECs vs ShRNA-Ctrl-transduced Ctrl N BMECs; p<0.0001, ShRNA-ECE-1-transduced Mg^2+^-free N BMECs vs ShRNA-ECE-1-transduced Ctrl N BMECs), claudin-5 (n=5; p<0.0001, ShRNA-ECE-1-transduced Ctrl N BMECs vs ShRNA-Ctrl-transduced Ctrl N BMECs; p<0.0001, ShRNA-ECE-1-transduced Mg^2+^-free N BMECs vs ShRNA-Ctrl-transduced Mg^2+^-free N BMECs; p=0.0005, ShRNA-Ctrl-transduced Mg^2+^-free N BMECs vs ShRNA-Ctrl-transduced Ctrl N BMECs; p<0.0001, ShRNA-ECE-1-transduced Mg^2+^-free N BMECs vs ShRNA-ECE-1-transduced Ctrl N BMECs), and ZO-1 (n=5; p<0.0001, ShRNA-ECE-1-transduced Ctrl N BMECs vs ShRNA-Ctrl-transduced Ctrl N BMECs; p<0.0001, ShRNA-ECE-1-transduced Mg^2+^-free N BMECs vs ShRNA-Ctrl-treated Mg^2+^-free N BMECs; p=0.0011, ShRNA-Ctrl-transduced Mg^2+^-free N BMECs vs ShRNA-Ctrl-transduced Ctrl N BMECs; p<0.0001, ShRNA-ECE-1-transduced Mg^2+^-free N BMECs vs ShRNA-ECE-1-transduced Ctrl N BMECs) in Control neuron and Mg^2+^-free neuron co-cultured BMECs transduced with Control-ShRNA or ECE-1-ShRNA lentivirus. Values are expressed as means ± S.E.M. Statistical analyses were performed using one-way ANOVA followed by Tukey’s test.

**
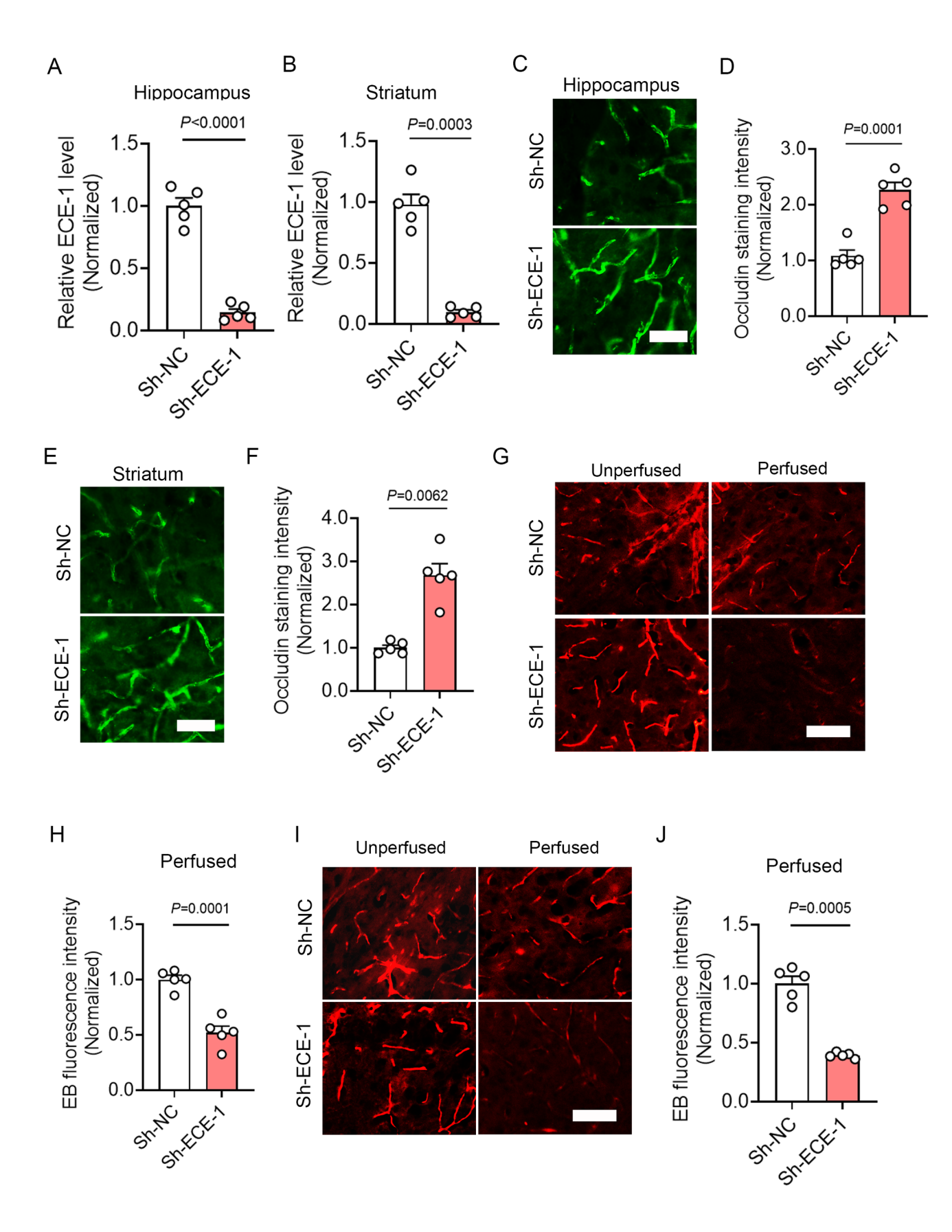
**

**Figure S14.** Knockdown of ECE-1 mitigates BBB damage in the hippocampus and striatum following SE. A, B) Quantative RT-PCR analyses of ECE-1 mRNA levels in the hippocampus (n=5, p<0.0001, Sh-ECE-1 vs Sh-NC) and striatum (n=5, p=0.0003, Sh-ECE-1 vs Sh-NC) of Sh-NC- and Sh-ECE-1-treated SE-24h mice. C) Representative images of Occludin immunostaining in the hippocampus of Sh-NC- and Sh-ECE-1-treated SE-24h mice. D) Bar graph showing the quantification of the mean fluorescence intensity of Occludin staining (n=5, p=0.0001, Sh-ECE-1 vs Sh-NC). E) Representative images of Occludin immunostaining in the striatum of Sh-NC- and Sh-ECE-1-treated SE-24h mice. F) Bar graph showing the quantification of the mean fluorescence intensity of Occludin staining (n=5, p=0.0062, Sh-ECE-1 vs Sh-NC). G) Evans blue (EB) fluorescence detected in the hippocampus of Sh-ECE-1- and Sh-NC-treated mice before and after perfusion. H) Bar graph showing the quantification of the mean fluorescence intensity of EB in the hippocampus of Sh-ECE-1- and Sh-NC-treated perfused mice (n=5, p=0.0001, Sh-ECE-1 vs Sh-NC). I) EB fluorescence detected in the striatum of Sh-ECE-1- and Sh-NC-treated mice before and after perfusion. J) Bar graph showing the quantification of the mean fluorescence intensity of EB in the striatum of Sh-ECE-1- and Sh-NC-treated perfused mice (n=5, p=0.0005, Sh-ECE-1 vs Sh-NC). Values are presented as means ± S.E.M. Statistical analyses was performed using an unpaired two-tailed Student’s t-test (A, D, F, H) and an unpaired two-tailed Welch’s t-test (B, J). Scale bar=25 µm.


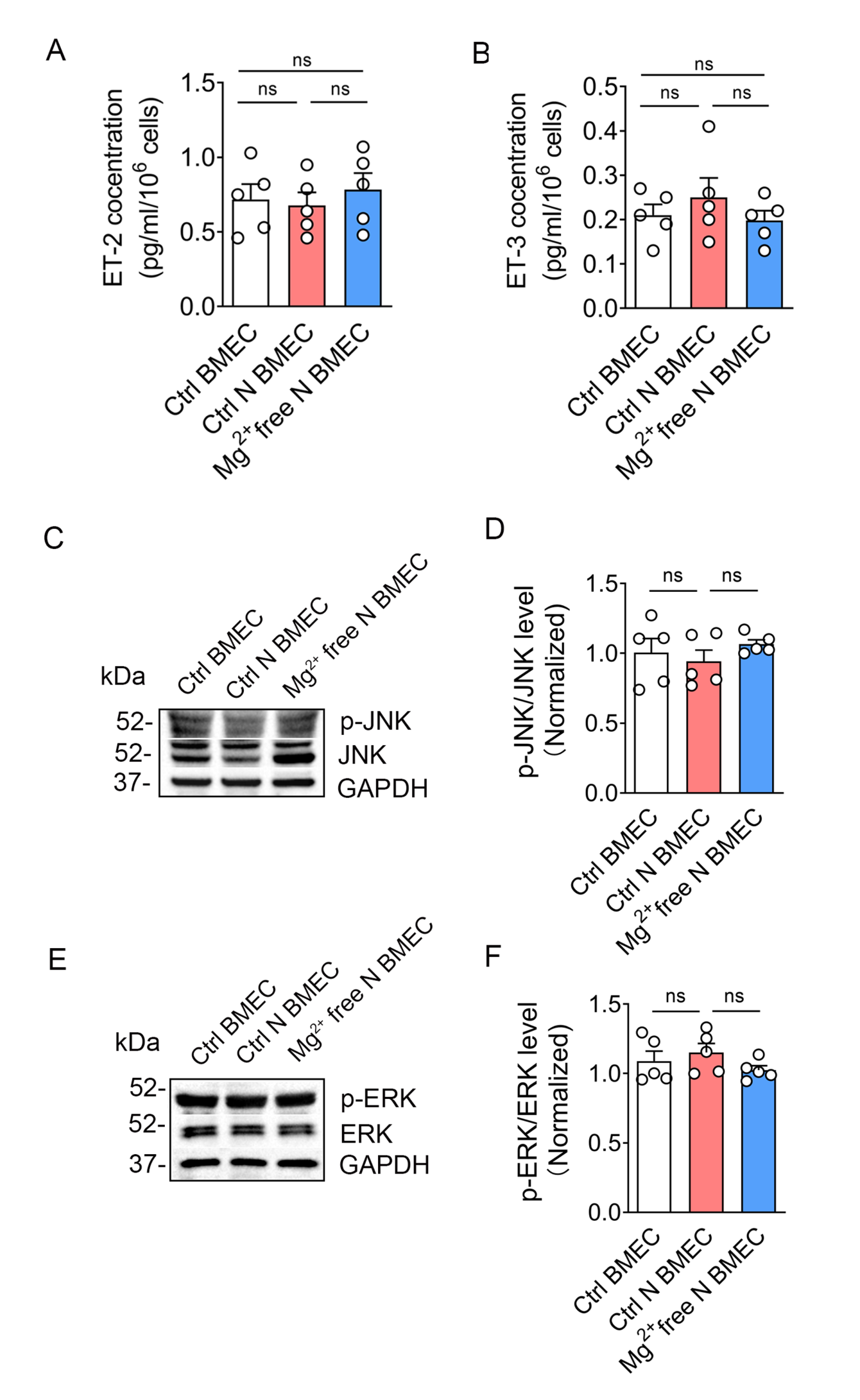


**Figure S15.** Endothelin level and MAPK pathway activation in BMECs under epileptic condition. A) Elisa analyses of ET-2 levels in Ctrl BMEC, Ctrl N BMEC, and Mg²⁺ free N BMEC (n=5; ns, Ctrl N BMEC vs Ctrl BMEC; ns, Mg²⁺ free N BMEC vs Ctrl N BMEC; ns, Mg²⁺ free N BMEC vs Ctrl BMEC). B) Elisa analyses of ET-3 levels in Ctrl BMEC, Ctrl N BMEC, and Mg²⁺ free N BMEC (n=5; ns, Ctrl N BMEC vs Ctrl BMEC; ns, Mg²⁺ free N BMEC vs Ctrl N BMEC; ns, Mg²⁺ free N BMEC vs Ctrl BMEC). C) Western blot analyses showing p-JNK/JNK levels in in Ctrl BMEC, Ctrl N BMEC, and Mg²⁺ free N BMEC. D) Bar graph quantifying p-JNK/JNK levels (n=5; ns, Ctrl N BMEC vs Ctrl BMEC; ns, Mg²⁺ free N BMEC vs Ctrl N BMEC). E) Western blot analyses showing p-ERK/ERK levels in Ctrl BMEC, Ctrl N BMEC, and Mg²⁺ free N BMEC. F) Bar graph quantifying p-ERK/ERK levels (n=5; ns, Ctrl N BMEC vs Ctrl BMEC; ns, Mg²⁺ free N BMEC vs Ctrl N BMEC). Data are presented as means ± S.E.M. Statistical analyses was performed using one-way ANOVA followed by Tukey’s post hoc test.


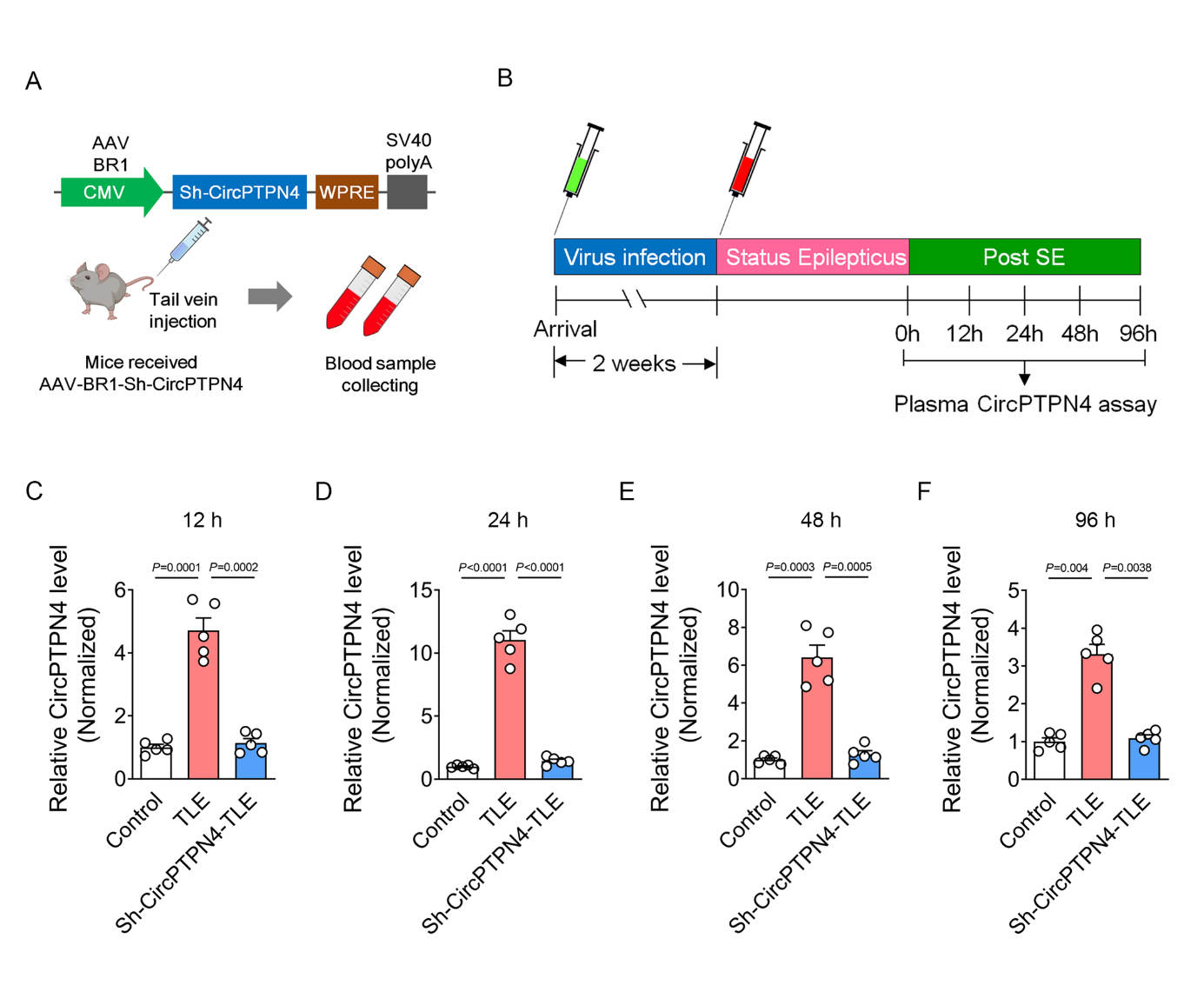


**Figure S16.** Plasma CircPTPN4 was derived from brain endothelial CircPTPN4. A) Schematic diagram depicting AAV constructs for CircPTPN4-targeting shRNA (Sh-CircPTPN4). The virus is administered by tail vein injection. Blood samples were collected for further experiment. B) Experimental design schematic. Two weeks after viral injection, mice underwent SE induction followed by quantative RT-PCR analyses of plasma CircPTPN4 at 0, 12, 24, 48 and 96 hours post-SE, respectively. C-F) Quantative RT-PCR analyses validating CircPTPN4 levels in Control, TLE and Sh-CircPTPN4-TLE mice at 12h (n=5; p=0.0001, SE-12h vs Control; p=0.0002, SE-12h vs Sh-CircPTPN4-SE-12h), 24h (n=5; p<0.0001, SE-24h vs Control; p<0.0001, SE-24h vs Sh-CircPTPN4-SE-24h), 48h (n=5; p=0.0003, SE-48h vs Control; p=0.0005, SE-48h vs Sh-CircPTPN4-SE-48h) and 96h (n=5; p=0.004, SE-96h vs Control; p=0.0038, SE-96h vs Sh-CircPTPN4-SE-96h) post SE. Data are presented as means ± S.E.M. Statistical analyses was performed using one-way ANOVA followed by Tukey’s post hoc test.

**
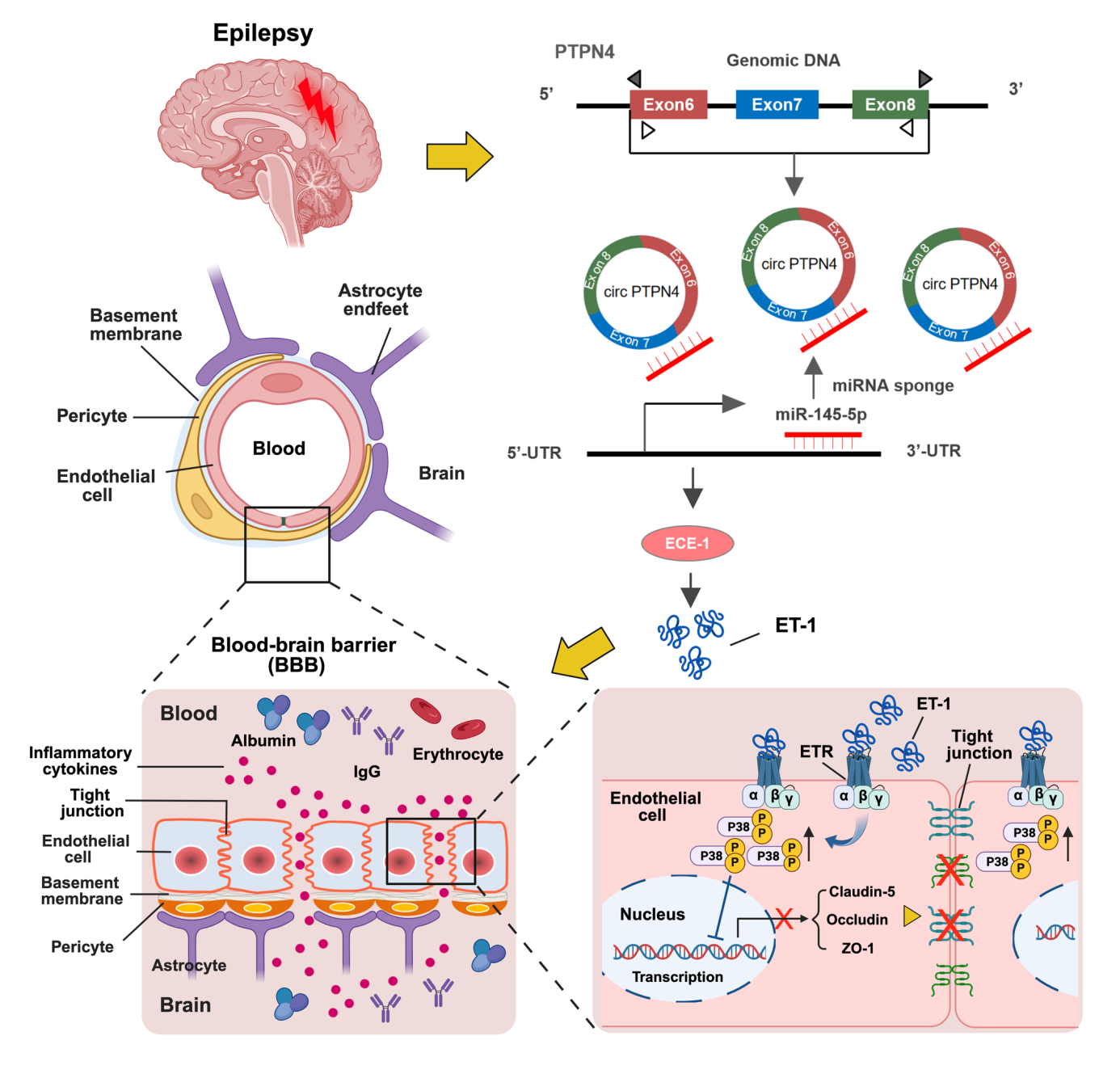
**

**Figure S17.** Proposed mechanism of CircPTPN4-mediated BBB damage during early epileptogenesis. Epileptogenic conditions induce back-splicing of exons 6, 7, and 8 of the PTPN4 gene to generate CircPTPN4. CircPTPN4 facilitates Endothelin Converting Enzyme-1 (ECE-1) expression by competitively sequestering miR-145a-5p. Elevated ECE-1 catalyzes increased production of Endothelin-1 (ET-1). Subsequent ET-1 upregulation activates the p38/MAPK signaling pathway, leading to downregulation of tight junction protein expression in brain microvascular endothelial cells (BMECs). This impairs BBB integrity, increasing permeability and facilitating inflammatory cytokine infiltration into the brain parenchyma, ultimately potentiating seizure susceptibility. The graphical abstract was created with permission from BioRender.

**Supplementary Tables**

**Table S1. Primers used for quantitative RT-PCR analyses**

| Name | Sequence 5’—3’ |
| --- | --- |
| ECE-1 | Forward: TCTCCGAGGGCGATGTGTA  Reverse: CTTCTCCACCGAGGTCCGA |
| claudin-5 | Forward: GCAAGGTGTATGAATCTGTGCT  Reverse: GTCAAGGTAACAAAGAGTGCCA |
| Occludin | Forward: TTGAAAGTCCACCTCCTTACAGA  Reverse: CCGGATAAAAAGAGTACGCTGG |
| ZO-1 | Forward: GCCGCTAAGAGCACAGCAA  Reverse: TCCCCACTCTGAAAATGAGGA |
| GAPDH | Forward: TCTTCCCCTTTTAATGGTCAGTGTAC  Reverse: TCTTCCCCTTTTAATGGTCAGTGTAC |
| has_circPTPN4  convergent primer | Forward: GTCCTTCTAATACTGCTGCCCTTT  Reverse: TGACTGATCGTAGTCTCCAAGTTCA |
| mmu_circPTPN4 convergent primer | Forward: CCTTGTAACACTGCTGCCCTTT  Reverse: TTCTGACTGATTGTAGTCTCCAAGTTC |
| has_ CircPTPN4 divergent primer-1 | Forward: CACAGAACTTGTCAGGCTACCTC  Reverse: ATGAAGCTAAAAGGGCAGCA |
| has_ CircPTPN4 divergent primer-2 | Forward: GAACTTGTCAGGCTACCTCTCAG  Reverse: ATGAAGCTAAAAGGGCAGCA |
| has_ CircPTPN4 divergent primer-3 | Forward: GTCACAGAACTTGTCAGGCTACC  Reverse: ATGAAGCTAAAAGGGCAGCA |
| has_ CircPTPN4 divergent primer-4 | Forward: CGATCAGTCAGAGAACTTCTCA  Reverse: ATGAAGCTAAAAGGGCAGCA |
| mmu_ CircPTPN4 divergent primer-1 | Forward: CTTGTAACACTGCTGCCCTT  Reverse: TGCTGCTGATGTAACTTTGCA |
| mmu_ CircPTPN4 divergent primer-2 | Forward: TCCTGTCCTTGTAACACTGCT  Reverse: GCTGCTGATGTAACTTTGCAATT |
| CircPCNX | Forward: CACTTTTGGCCCTGTTGAT  Reverse: TCTTCTGTTGGGCCGTAAGT |
| miR-145a-5p | Forward: CGGTCCAGTTTTCCCAGGA  Reverse: AGTGCAGGGTCCGAGGTATT |
| miR-28a-5p | Forward: CGCGAAGGAGCTCACAGTCT  Reverse: AGTGCAGGGTCCGAGGTATT |
| U6 | Forward: GCTTCGGCAGCACATATACTAAAAT  Reverse: CGCTTCACGAATTTGCGTGTCAT |

**Table S2. Antibody information**

| Antibody | Vendor | Catalog Number | Concentration |
| --- | --- | --- | --- |
| GAPDH Monoclonal antibody | Proteintech Technology | 60004-1-Ig | 1:2000 (WB) |
| Anti-NeuN antibody | Abcam | ab177487 | 1:200 (IF) |
| PECAM-1(CD31) | Santa Cruz | Sc-18916 | 1:100 (IF) |
| Occludin Monoclonal antibody | Invitrogen | OC-3F10 | 1:200 (IF)  1:1500 (WB) |
| Anti-claudin-5 antibody | Abcam | Ab15106 | 1:1500 (WB) |
| ZO-1 Rabbit polyclonal antibody | Proteintech | 21773 | 1:1500 (WB) |
| Isolectin B4 | Sigma | L2140 | 1:50 (IF) |
| ECE-1 antibody | Santa Cruz | SC-376017 | 1:200 (IF)  1:1500 (WB) |
| PTPN4 Polyclonal Antibody | ThermoFisher | PA5-110241 | 1:1000 (WB) |
| Albumin Polyclonal antibody | Proteintech Technology | 16475-1-AP | 1:200 (IF) |
| Rabbit IgG Isotype Control Recombinant Antibody | Proteintech Technology | 98136-1-RR | 1:200 (IF) |
| Caveolin-1 Monoclonal antibody | Proteintech Technology | 66067-1-Ig | 1:200 (IF)  1:2000 (WB) |
| JNK Monoclonal antibody | Proteintech Technology | 66210-1-Ig | 1:3000 (WB) |
| Phospho-JNK(Tyr185)Recombinant antibody | Proteintech Technology | 80024-1-RR | 1:1500 (WB) |
| ERK1/2 Polyclonal antibody | Proteintech Technology | 11257-1-AP | 1:2000 (WB) |
| Phospho-ERK1/2 (Thr202/Tyr204) Polyclonal antibody | Proteintech Technology | 28733-1-AP | 1:1000 (WB) |
| p38 MAPK Monoclonal antibody | Proteintech Technology | 66234-1-Ig | 1:2000 (WB) |
| Phospho-p38 MAPK(Thr180/Tyr182)Polyclona antibody | Proteintech Technology | 28796-1-AP | 1:1000 (WB) |

**Table S3. Probe information**

| Name | Vendor | Sequence5’-3’ | Concentration |
| --- | --- | --- | --- |
| biotinylated CircPTPN4 probe (pull-down) | RiboBio | AAACATCATTTGCTGTTCAGTC | 100nM |
| biotinylated random probe (pull-down) | RiboBio | GCACATGTCATTATGACAT | 100nM |
| biotinylated WT miR-145-5p (pull-down) | Gene Pharma | GUCCAGUUUUCCCAGGAAUCCCUUU | 100 nM |
| biotinylated Mutant miR-145-5p (pull-down) | Gene Pharma | GUUCGAGUAUGCACAAGCAAUCCGA | 100 nM |
| biotinylated CircPTPN4 probe (FISH) | RiboBio | AAACATCATTTGCTGTTCAGTC | 50nM |
| digoxigenin-labeled miR-145a-5p probes(FISH) | RiboBio | TCCCAGGAATCCCT | 50nM |

**Table S4. shRNA vector sequence**

| Name | Sequence5’-3’ |
| --- | --- |
| Has_CircPTPN4 shRNA1 | Forward: GATCCGAAGAAGGATGTTGCAAACCACTTCAAGAGAGTGGTTTGCAACATCCTTCTTTTTTG  Reverse: AATTCAAAAAAGAAGAAGGATGTTGCAAACCACTTCAAGAGAGTGGTTTGCAACATCCTTCG |
| Has_CircPTPN4 shRNA2 | Forward: GATCCGATGCTTGAAGACGACATCCAATTCAAGAGATTGGATGTCGTCTTCAAGCATTTTTTG  Reverse: AATTCAAAAAAGATGCTTGAAGACGACATCCAATTCAAGAGATTGGATGTCGTCTTCAAGCAG |
| Has_CircPTPN4 shRNA3 | Forward: GATCCGAAGTGGTCTGTCCAAACGGAATTCAAGAGATTCCGTTTGGACAGACCACTTTTTTG  Reverse: AATTCAAAAAAGAAGTGGTCTGTCCAAACGGAATTCAAGAGATTCCGTTTGGACAGACCACG |
| Has_CircPTPN4 shRNA4 | Forward: GATCCGAATGTCTCTCACAACTAGCTTTTCAAGAGAAAGCTAGTTGTGAGAGACATTTTTTG  Reverse: AATTCAAAAAAGAATGTCTCTCACAACTAGCTTTTCAAGAGAAAGCTAGTTGTGAGAGACAG |
| mmu_CircPTPN4 shRNA1 | Forward: GATCCGAAGAATGTTGCAAACGACCTCTTCAAGAGAGAGGTCGTTTGCAACATTCTTTTTTG  Reverse: AATTCAAAAAAGAAGAATGTTGCAAACGACCTCTTCAAGAGAGAGGTCGTTTGCAACATTCG |
| mmu_CircPTPN4 shRNA2 | Forward: GATCCGTTTGTTAACTCTGAAAGCCTGTTCAAGAGACAGGCTTTCAGAGTTAACAAATTTTTTG  Reverse: AATTCAAAAAAGTTTGTTAACTCTGAAAGCCTGTTCAAGAGACAGGCTTTCAGAGTTAACAAAG |
| mmu_CircPTPN4 shRNA3 | Forward: GATCCGTATGATGTTCTACACACGCTTTTCAAGAGAAAGCGTGTGTAGAACATCATATTTTTTG  Reverse: AATTCAAAAAAGTATGATGTTCTACACACGCTTTTCAAGAGAAAGCGTGTGTAGAACATCATAG |
| mmu_CircPTPN4 shRNA4 | Forward: GATCCGAATCTGAGTAAGTTGACGGCTTTCAAGAGAAGCCGTCAACTTACTCAGATTTTTTG  Reverse: AATTCAAAAAAGAATCTGAGTAAGTTGACGGCTTTCAAGAGAAGCCGTCAACTTACTCAGAG |
| ECE1 shRNA1 | Forward: GATCCGGTGGTGTTGGTGGTACTTCTTTCAAGAGAAGAAGTACCACCAACACCACCTTTTTTG  Reverse: AATTCAAAAAAGGTGGTGTTGGTGGTACTTCTTTCAAGAGAAGAAGTACCACCAACACCACCG |
| ECE1 shRNA2 | Forward: GATCCGCTCAGGGCCAAACCTCTAATTTCAAGAGAATTAGAGGTTTGGCCCTGAGCTTTTTTG  Reverse: AATTCAAAAAAGCTCAGGGCCAAACCTCTAATTTCAAGAGAATTAGAGGTTTGGCCCTGAGCG |
| ECE1 shRNA3 | Forward: GATCCGCTTGCCCTCGAGAGACTATTTTCAAGAGAAATAGTCTCTCGAGGGCAAGCTTTTTTG  Reverse: AATTCAAAAAAGCTTGCCCTCGAGAGACTATTTTCAAGAGAAATAGTCTCTCGAGGGCAAGCG |
| ECE1 shRNA4 | Forward: GATCCGCTGGACAAAGTGTTTAATGATTCAAGAGATCATTAAACACTTTGTCCAGCTTTTTTG  Reverse: AATTCAAAAAAGCTGGACAAAGTGTTTAATGATTCAAGAGATCATTAAACACTTTGTCCAGCG |

**Table S5. CircPTPN4 sequence**

| Name | Sequence 5’—3’ |
| --- | --- |
| Has_CircPTPN4 | GTACCAGTATTTTTTGCAAATTAAACAAGACATTCTTACTGGAAGATTACCCTGTCCTTCTAATACTGCTGCCCTTTTAGCTTCATTTGCTGTTCAGTCTGAACTTGGAGACTACGATCAGTCAGAGAACTTGTCAGGCTACCTCTCAGATTATTCTTTCATTCCTAATCAACCTCAAGATTTTGAAAAAGAAATTGCAAAATTACATCAGCAACACAT |
|  |  |
| Mmu_CircPTPN4 | GTATCAGTACTTTTTGCAAATTAAGCAAGACATTCTTACTGGAAGATTATCCTGTCCTTGTAACACTGCTGCCCTTTTAGCATCATTTGCTGTTCAGTCTGAACTTGGAGACTACAATCAGTCAGAAAACTTGGCAGGCTACCTCTCAGATTATTCTTTCATTCCTAATCAACCTCAAGATTTTGAGAAAGAAATTGCAAAGTTACATCAGCAGCACGT |

**Table S6. miR-145a-5p mimics and inhibitors sequence**

| Name | Sequence5’-3’ |
| --- | --- |
| miR-145a-5p | GUCCAGUUUUCCCAGGAAUCCCU |
| Mimics Negative Control | AACUCAGUUCGAGUCACAGTT |
| miR-145a-5p mimics | GUCCAGUUUUCCCAGGAAUCCCUGGAUUCCUGGGAAAACUGGACUU |
| Inhibitor Negative Control | UAGUGGAUCGAGUGUGGAAUT |
| miR-145a-5p inhibitor | AGGGAUUCCUGGGAAAACUGGAC |
